# A revision of the Neotropical genus *Coptoborus* Hopkins (Coleoptera, Curculionidae, Scolytinae, Xyleborini)

**DOI:** 10.3897/zookeys.144.62246

**Published:** 2021-06-16

**Authors:** Sarah M. Smith, Anthony I. Cognato

**Affiliations:** 1 Department of Entomology, Michigan State University, 288 Farm Lane, East Lansing, Michigan 48824, USA Michigan State University East Lansing United States of America

**Keywords:** Ambrosia beetles, cacao, Neotropical, *
Theoborus
*

## Abstract

The Neotropical xyleborine ambrosia beetle genus *Coptoborus* Hopkins is reviewed. The following 40 *Coptoborus* species are described: *C. amplissimus***sp. nov.** (Peru), *C. asperatus***sp. nov.** (Ecuador), *C. barbicauda***sp. nov.** (French Guiana), *C. bettysmithae***sp. nov.** (Ecuador), *C. brevicauda***sp. nov.** (Ecuador), *C. brigman***sp. nov.** (Ecuador), *C. busoror***sp. nov.** (Ecuador), *C. capillisoror***sp. nov.** (Brazil), *C. chica***sp. nov.** (Suriname), *C. crassisororcula***sp. nov.** (Peru), *C. doliolum***sp. nov.** (Ecuador), *C. erwini***sp. nov.** (Ecuador), *C. furiosa***sp. nov.** (Ecuador), *C. galacatosae***sp. nov.** (Ecuador), *C. hansen***sp. nov.** (Brazil), *C. incomptus***sp. nov.** (Peru), *C. janeway***sp. nov.** (Peru), *C. katniss***sp. nov.** (Ecuador), *C. leeloo***sp. nov.** (Ecuador), *C. leia***sp. nov.** (Ecuador, Suriname), *C. leporinus***sp. nov.** (Peru), *C. martinezae***sp. nov.** (Ecuador), *C. murinus***sp. nov.** (Ecuador), *C. newt***sp. nov.** (Peru), *C. osbornae***sp. nov.** (Ecuador), *C. panosus***sp. nov.** (French Guiana), *C. papillicauda***sp. nov.** (Suriname), *C. pilisoror***sp. nov.** (Ecuador), *C. ripley***sp. nov.** (Ecuador), *C. sagitticauda***sp. nov.** (Guyana), *C. sarahconnor***sp. nov.** (Brazil), *C. scully***sp. nov.** (Ecuador), *C. sicula***sp. nov.** (Ecuador), *C. sororcula***sp. nov.** (Peru), *C. starbuck***sp. nov.** (Ecuador), *C. trinity***sp. nov.** (Brazil), *C. uhura***sp. nov.** (Peru), *C. vasquez***sp. nov.** (Panama), *C. vrataski***sp. nov.** (Brazil), and *C. yar***sp. nov.** (Ecuador). Seventeen new combinations are given: *Coptoborus amazonicus* (Petrov, 2020) **comb. nov.**, *C. atlanticus* (Bright & Torres, 2006) **comb. nov.**, *C. bellus* Bright & Torres, 2006 **comb. nov.**, *C. coartatus* (Sampson, 1921) **comb. nov.**, *C. crinitulus* (Wood, 1974) **comb. nov.**, *C. exilis* (Schedl, 1934) **comb. nov.**, *C. incultus* (Wood, 1975) **comb. nov.**, *C. magnus* (Petrov, 2020) **comb. nov.**, *C. micarius* (Wood, 1974) **comb. nov.**, *C. obtusicornis* (Schedl, 1976) **comb. nov.**, *C. paurus* (Wood, 2007) **comb. nov.**, *C. pristis* (Wood, 1974) **comb. nov.**, *C. pseudotenuis* (Schedl, 1936) **comb. nov.**, *C. puertoricensis* (Bright & Torres, 2006) **comb. nov.**, *C. ricini* (Eggers, 1932) **comb. nov.**, *C. semicostatus* (Schedl, 1948) **comb. nov.**, *C. tristiculus* (Wood, 1975) **comb. nov.**, and *C. villosulus* (Blandford, 1898) **comb. nov.** Two new synonyms are proposed: *Coptoborus* Hopkins, 1915 (= *Theoborus* Hopkins, 1915 **syn. nov.**) and *Coptoborus villosulus* (Blandford, 1898) (= *Theoborus theobromae* Hopkins, 1915 **syn. nov.**). *Xyleborus neosphenos* Schedl, 1976 **comb. res.** is removed from *Coptoborus*. The revised genus now contains 77 species and a key to their identification is provided.

## Introduction

The diversity of Neotropical scolytine beetles is largely undescribed. Estimates of the Ecuadorian and Peruvian faunas suggest that the fauna is ~3–4 times greater than currently known (Smith et al. 2017; Dole et al. 2021) and recent taxonomic reviews have revealed several new genera and new species (e.g. Dole and Cognato 2007; Petrov and Mandelshtam 2009; Smith and Cognato 2010; Petrov and Mandelshtam 2010; Petrov 2014; Smith 2017; Atkinson 2018; Cognato 2018; Petrov and Mandelshtam 2018; Bright 2019; Atkinson 2020; Jordal and Smith 2020; Pérez Silva et al. 2020). The Xyleborini ambrosia beetles have a worldwide diversity of at least 1200 species but have received limited attention in the Neotropics, the region where most of the scolytine diversity lies (Hulcr et al. 2015). In the last major reviews of the Central and South American xyleborine faunas, ~175 species have been recorded and more are likely to be discovered (Wood 1982, 2007; Smith et al. 2017). Indeed, the Amazonian canopy is a source of untapped diversity which may yield an additional 40–80% as compared to the currently known fauna (Dole et al. 2021). Diversification into different habitats and the highly inbred nature of xyleborines may explain the radiation of endemic genera and species that occurred after the colonization of the Americas in the past 15 million years (Cognato et al. 2011; Jordal and Cognato 2012; Gohli et al. 2016). Of these ambrosia beetle genera, *Coptoborus* Hopkins, 1915 and *Theoborus* Hopkins, 1915 are similar in morphology, closely related and comprise ~30 species (Wood 2007; Cognato et al. 2011). *Theoborus coartatus* (Sampson, 1921), *T. theobromae* Hopkins, 1915, *T. villosulus* (Blandford, 1898), *Coptoborus tolimanus* (Eggers, 1928), and *C. vespatorius* (Schedl, 1931) have long been recognized as pests of cacao (Terra 1987) and a newly emerging pest, *Coptoborus ochromactonus* Smith & Cognato, 2014 (Stilwel et al. 2014) threatens balsa wood production (Stilwel et al. 2014; Castro et al. 2019; Martínez et al. 2020), but otherwise, the remaining species are assumed benign. An accumulation of recently collected specimens and museum loans which represents hand collected material from dispersed Neotropical localities and specimens from the Ecuadorian Amazonian canopy, presents an opportunity to re-examine the generic limits of *Coptoborus* and *Theoborus* and to contribute to the knowledge of the alpha diversity of xyleborines.

### Taxonomic history

*Coptoborus* was described for three species, *Coptoborus emarginatus* Hopkins, 1915, the type of the genus, and two additional species, *C. palmeri* Hopkins, 1915 and *C. terminaliae* Hopkins, 1915. *Coptoborus emarginatus* was described from Guatemala while the other species were described from Indonesia and the Philippines, respectively. Hopkins’s concept of *Coptoborus* was primarily based on antennal characters, specifically the “five jointed funicle (including pedicle) with the segment 1 [pedicle] large and broad, segment 5 [segment 4] much broader than segment 2 [segment 1]; club short, broader than long, sides subequally rounded, anterior face with two sinuate sutures, posterior face with one broadly procurved subapical suture; anterior margin of pronotum fairly rugose; eyes large, elliptical, emarginate”. Wood (1980) attempted to form a meaningful classification of the Xyleborini and placed the Paleotropical genus *Streptocranus* Schedl, 1939 in synonymy with *Coptoborus*. Later, Wood (1982) recognized *Coptoborus* as a subgenus of *Xyleborus*. This action led to homonymy with *Xyleborus emarginatus* Eichhoff, 1878 and the type of *Coptoborus*, *C. emarginatus* (Hopkins, 1915) and the latter was given the permanent replacement name *Xyleborus vespatorius* (Schedl 1931). Wood (1986) considered *Coptoborus* a valid genus and retained *Streptocranus* as a junior synonym. Additional species were incorporated into *Coptoborus* from both the Neotropics and Paleotropics (Wood and Bright 1992). Wood (2007) diagnosed *Coptoborus* based on “protibia with posterior face flat, metatibia almost never with more than 7 socketed teeth”, “posterior third of elytra attenuate or acuminate, narrowly rounded behind, suture often emarginate, 1 or more interstriae sometimes armed by small denticles, and posterior face of the club with two sutures.” Wood recognized two sutures on the posterior face of the antennal club instead of one subapical suture which is the most notable difference to Hopkins’s concept. The posterior face of the *C. vespatorius* club has two sutures, but the second is faint and not as prominent as the first. Wood (2007) also described several additional species from the Neotropics. Hulcr et al. (2007) reviewed generic characters in the Xyleborini and found that *Streptocranus* was not congeneric with *Coptoborus* and removed it from synonymy. Hulcr (2010) recognized that both *C. palmeri* and *C. teminaliae* belonged in *Debus* Hulcr & Cognato, 2010 as synonyms of *Xyleborus emarginatus* Eichhoff, 1878. The phylogenetic distinction between *Coptoborus* and *Streptocranus* was later confirmed with molecular data (Cognato et al. 2011). Recently, Bright (2019) restricted the concept of *Coptoborus* to include only the type species, *C. vespatorius*, which has the elytra deeply sulcate, a distinctly elevated and costate interstriae 3, and each elytron separately rounded and produced. This narrow concept of *Coptoborus* is not supported by a molecular phylogeny (Cognato et al. 2011).

*Theoborus* was described by Hopkins (1915) to accommodate a single species, *Theoborus theobromae* Hopkins, 1915. Like *Coptoborus*, the concept of *Theoborus* was also based primarily on the antennal club, “funicle 5-jointed [including pedicle], joint 5 [segment 4] broad, 2 [segment 1] not longer than 3 (segment 2] and 4 [segment 3] together; club broad, with sides equally rounded, anterior and posterior faces each with two sutures; eyes small, elliptical, broadly emarginate.” Unlike *Coptoborus*, the generic status of *Theoborus* was never in doubt presumably because the two sutures on the posterior face of the club are much more readily apparent on the type species. Wood (1982) distinguished the genus from other Neotropical Xyleborini genera by the presence of two sutures on the posterior face of the club and the flat posterior face of the protibia. Wood (1982) moved eight species from *Xyleborus* to *Theoborus* and additional species were added (Wood and Bright 1992). Wood and Bright (1992) also moved one species from *Theoborus* to *Xyleborus* and placed one species in synonymy. Wood (2007) described one additional species and maintained his 1982 diagnosis but added additional characters separating it from *Coptoborus* in the Xyleborini key including “posterior fourth of elytra comparatively broad, rather broadly rounded behind, suture never emarginate; declivital interstriae 1–3 similar, tubercles minute, if present, body comparatively stout, less than 2.6 × as long as wide”. Most recently Smith et al. (2020) considered the Panamanian species *Theoborus molestulus* (Wood, 1975b) a synonym of the introduced Asian species *Euwallacea perbrevis* (Schedl, 1951).

Wood’s (1982, 2007) generic concepts are very similar and essentially species are defined as *Coptoborus* if the body shape is attenuate, acuminate or narrowly rounded and elongate or *Theoborus* if the body shape is rounded and stout. Xyleborine genera are primarily defined based on characters of the antennal club, protibia, pronotal shape, mycangial tufts and scutellum (Hulcr et al. 2007) rather than overall body shape which has been shown to be convergent (Hulcr et al. 2007; Cognato et al. 2020a; Smith et al. 2020). The striking similarity of the genera questions their taxonomic validity.

## Materials and methods

Examined specimens were obtained via canopy fogging, our own field expeditions targeting scolytines and through loans from several institutions. Canopy fogging specimens came from Terry Erwin’s long-term Ecuadorian canopy fogging project in the primary forest in Yasuní National Park at the Tiputini Biodiversity Station and Okone Gare Station located in the lowland Amazonian forest of Orellana province. Sites were sampled twice a year during each of the rainy (May–October) and dry seasons (November – April) and collection methods are detailed in Erwin et al. (2005). Our field collected specimens were obtained from Brazil, Ecuador, Guyana, Panama, and Peru and were collected either by direct excision from their galleries or with panel flight intercept traps, “Petrov FIT”, as detailed by Nikulina et al. (2015). All descriptions, keys and diagnoses are based on females as males are largely unknown, rarely encountered, and not often present without a female of the same species. Specimens were amassed and examined from the following entomological collections:

**APP** Alexander V. Petrov private collection, Moscow, Russia;

**CNCI**Canadian National Collection of Insects, Ottawa, Canada;

**CSCA**California State Collection of Arthropods, Sacramento, USA;

**FSCA**Florida State Collection of Arthropods, Gainesville, USA;

**ICB**Instituto de Ciencias Biologicas, Escuela Politécnica Nacional, Quito, Ecuador;

**MECN**Museo Ecuatoriano de Ciencias Naturales, Quito, Ecuador;

**MEFEIS** Museu de Entomologia da FEIS/UNESP, Ilha Solteira, São Paulo State, Brazil;

**MIIZ** Zoological Museum, Museum and Institute of Zoology, Polish Academy of Science, Warsaw, Poland;

**MNHN**Muséum National d’Histoire Naturelle, Paris, France;

**MUSM**Museo de Historia Natural, Universidad Nacional Mayor de San Marcos, Lima, Peru;

**MSUC**Michigan State University Arthropod Research Collection, East Lansing, USA;

**MZUSP**Museu de Zoologia da Universidade de São Paulo, São Paulo, Brazil;

**NHMUK**Natural History Museum, London, United Kingdom;

**NHMW**Naturhistorisches Museum Wien, Austria;

**NMNH**Natural Museum of Natural History, Smithsonian Institution, Washington, D.C., USA;

**NHMB**Hungarian Natural History Museum, Budapest, Hungary;

**NZCS**National Zoological Collection of Suriname, Paramaribo, Suriname;

**PUCE**Museo de Zoología, Pontificia Universidad Católica del Ecuador, Quito, Ecuador;

**SEMC**Biodiversity Institute & Natural History Museum, The University of Kansas, Lawrence, USA;

**TAMU**Insect Collection, Texas A & M University, College Station, USA;

**UCDC**R.M. Bohart Museum of Entomology, University of California Davis, Davis, USA;

**UTIC**University of Texas Insect Collection, Austin, USA;

**UAAM**University of Arkansas Arthropod Museum, Fayetteville, USA;

**ZMMU**Zoological Museum, Moscow State University, Moscow, Russia.

Specimens were photographed by SMS with a Visionary Digital Passport II system (Dun Inc., Palmyra, VA) using a Canon EOS 5D Mark II, 65.0 mm Canon Macro photo lens, two Dynalite (Union, NJ) MH2015 road flash heads, Dynalite RoadMax MP8 power pack and a Stack Shot (Cognisys, Inc, Traverse City, MI). Montage images were assembled using Helicon Focus Mac Pro 6.7.1 (Helicon Soft, Kharkov, Ukraine).

Specimens were examined using Leica (Wetzlar, Germany) MZ6 and MZ16 stereomicroscopes and illuminated with an Ikea Jansjö LED work lamp (Delft, Netherlands). Length was measured from pronotum apex to the apex of the declivity, width was measured at the widest point of the pronotum and a maximum of five specimens per species were measured. Measurements were taken of specimens measured and reported by Wood (2007) and these were typically found to be 0.15–0.2 mm shorter than ours, and in the case of *C. cuneatus*, 0.4–0.5 mm smaller. This calibration error has been noted before as 0.1–0.15 mm for specimens 2.0–3.0 mm (Jordal 1998; L.R. Kirkendall, pers. comm. 9 Dec 2020). To maintain accuracy, specimens that we were unable to directly measure have a citation given for their length. Pedicel is not included in the number of funicle segments. Pronotal (dorsal and lateral) and antennal club types follow those proposed by Hulcr et al. (2007) and further illustrated by Smith et al. (2020).

Distribution and host records were aggregated from the following publications: Atkinson and Equihua Martinez 1985, 1986; Estrada Valencia and Atkinson 1989; Dall’oglio and Peres-Filho 1997; Bright and Skidmore 2002; Wood 2007; Pérez de la Cruz et al. 2009; Atkinson et al. 2010; Bright 2014; Sandoval Rodríguez et al. 2017; Smith et al. 2017; Atkinson 2018; Bright 2019; Del Carmen Gerónimo-Torres et al. 2019; Gomez et al. 2019. New locality records are denoted with an asterisk. A list of species and their occurrence by country or territory (e.g. Puerto Rico) are given in Table 1.

**Table 1.** Distribution of *Coptoborus* species by country or territory. X = recorded.

Species	Argentina	Bahamas	Barbados	Bolivia	Brazil	Colombia	Costa Rica	Cuba	Dominica	Dominican Republic	Ecuador	French Guiana	Grenada	Guadeloupe	Guatemala	Guyana	Honduras	Jamaica	Martinique	Mexico	Montserrat	Netherlands Antilles	Panama	Paraguay	Peru	Puerto Rico	Saint Lucia	Saint Vincent and the Grenadines	Suriname	Trinidad	United States	Venezuela	Africa (Introduced)
*Coptoborus amazonicus*																									X								
*Coptoborus amplissimus*																									X								
*Coptoborus artetenuis*				X																													
*Coptoborus asperatus*											X																						
*Coptoborus atlanticus*										X																X							
*Coptoborus attenuatus*					X																												
*Coptoborus barbicauda*												X																					
*Coptoborus bellus*													X													X							
*Coptoborus bettysmithae*											X																						
*Coptoborus brevicauda*											X																						
*Coptoborus brigman*											X																						
*Coptoborus busoror*											X																						
*Coptoborus capillisoror*					X																												
*Coptoborus carumbensis*					X																			X									
*Coptoborus catulus*					X						X									X			X		X				X			X	
*Coptoborus chica*																													X				
*Coptoborus coartatus*					X	X	X				X									X			X	X						X			
*Coptoborus cracens*					X						X														X								
*Coptoborus crassisororcula*																									X								
*Coptoborus crinitulus*																							X				X					X	
*Coptoborus cuneatus*																							X		X							X	
*Coptoborus doliolum*											X																						
*Coptoborus erwini*											X																						
*Coptoborus exilis*							X						X										X				X						
*Coptoborus exutus*							X																										
*Coptoborus furiosa*											X																						
*Coptoborus galacatosae*											X																						
*Coptoborus gentilis*					X																												
*Coptoborus gracilens*					X						X	X													X								
*Coptoborus hansen*					X																												
*Coptoborus incomptus*																									X								
*Coptoborus incultus*																				X			X										
*Coptoborus inornatus*					X																												
*Coptoborus janeway*																									X								
*Coptoborus katniss*											X																						
*Coptoborus leeloo*											X																						
*Coptoborus leia*											X																		X				
*Coptoborus leporinus*																									X								
*Coptoborus magnus*																									X								
*Coptoborus martinezae*											X																						
*Coptoborus micarius*							X																X										
*Coptoborus murinus*											X																						
*Coptoborus newt*																									X								
*Coptoborus nudulus*					X						X														X								
*Coptoborus obtusicornis*					X		X				X														X								
*Coptoborus ochromactonus*											X																						
*Coptoborus osbornae*											X																						
*Coptoborus panosus*												X																					
*Coptoborus papillicauda*																													X				
*Coptoborus paurus*							X																										
*Coptoborus pilisoror*											X																						
*Coptoborus pristis*					X		X				X				X								X		X								
*Coptoborus pseudotenuis*						X	X		X		X		X	X					X	X			X		X		X	X		X	X	X	
*Coptoborus puertoricensis*										X																X							
*Coptoborus ricini*				X	X	X	X			X							X	X		X					X						X	X	X
*Coptoborus ripley*											X																						
*Coptoborus sagitticauda*																X																	
*Coptoborus sarahconnor*					X																												
*Coptoborus schulzi*											X																		X				
*Coptoborus scully*											X																						
*Coptoborus semicostatus*				X	X																												
*Coptoborus sicula*											X																						
*Coptoborus silviasilasi*																				X													
*Coptoborus solitariformis*					X																												
*Coptoborus sororcula*																									X								
*Coptoborus spicatus*																													X				
*Coptoborus starbuck*											X																						
*Coptoborus subtilis*					X																												
*Coptoborus tolimanus*					X	X	X				X	X								X			X									X	
*Coptoborus trinity*					X																												
*Coptoborus tristiculus*					X						X																						
*Coptoborus uhura*																									X								
*Coptoborus vasquez*																							X										
*Coptoborus vespatorius*	X				X	X	X				X	X	X			X				X					X		X					X	
*Coptoborus villosulus*	X	X	X	X	X	X	X	X	X	X	X	X	X	X	X				X	X	X	X	X		X		X	X				X	
*Coptoborus vrataski*					X																												
*Coptoborus yar*											X																						
Total number of species	2	1	1	4	24	6	12	1	2	4	35	6	5	2	2	2	1	1	2	9	1	1	12	2	22	3	5	2	6	2	2	8	1

### Etymology

Perhaps xyleborines are true Amazons given that females dominate the dwarfed flightless males in size and in number. Females disperse to and bore into new host trees to start fungal gardens to feed their offspring (Smith and Hulcr 2015; Kirkendall et al. 2015). Many perish on this journey but those that survive propagate new generations. For millions of years, these intrepid beetles have colonized new lands which led to new lineages and species across the tropics (e.g., Gohli et al 2016; Cognato et al 2018). As a result, the nearly 1200 species exhibit an extraordinary range of morphological diversity. In recognition of these “adventurous” and “unearthly-looking” pioneers, many of the species described herein are named to honor iconic strong female role models of science fiction movies and television. Most of these characters were sources of inspiration for SMS throughout her adolescent life and admiration by AIC.

## Results

### Checklist

*Coptoborus* Hopkins

*Theoborus* Hopkins, syn. nov.

*Coptoborus amazonicus* (Petrov, 2020) comb. nov.

*Coptoborus amplissimus* sp. nov.

*Coptoborus artetenuis* (Schedl, 1973)

*Coptoborus asperatus* sp. nov.

*Coptoborus atlanticus* (Bright & Torres, 2006) comb. nov.

*Coptoborus attenuatus* Wood, 2007

*Coptoborus barbicauda* sp. nov.

*Coptoborus bellus* (Bright & Torres, 2006) comb. nov.

*Coptoborus bettysmithae* sp. nov.

*Coptoborus brevicauda* sp. nov.

*Coptoborus brigman* sp. nov.

*Coptoborus busoror* sp. nov.

*Coptoborus capillisoror* sp. nov.

*Coptoborus carumbensis* Wood, 2007

*Coptoborus catulus* (Blandford, 1898)

*Xyleborus intricatus* Schedl, 1948

*Coptoborus chica* sp. nov.

*Coptoborus coartatus* (Sampson, 1921) comb. nov.

*Xyleborus artecuneolus* Schedl, 1939

*Coptoborus cracens* Wood, 2007

*Coptoborus crassisororcula* sp. nov.

*Coptoborus crinitulus* (Wood, 1974) comb. nov.

*Coptoborus cuneatus* (Eichhoff, 1878)

*Coptoborus doliolum* sp. nov.

*Coptoborus erwini* sp. nov.

*Coptoborus exilis* (Schedl, 1934) comb. nov.

*Coptoborus exutus* (Wood, 1974)

*Coptoborus furiosa* sp. nov.

*Coptoborus galacatosae* sp. nov.

*Coptoborus gentilis* (Schedl, 1972)

*Coptoborus gracilens* Wood, 2007

*Coptoborus hansen* sp. nov.

*Coptoborus incomptus* sp. nov.

*Coptoborus incultus* (Wood, 1975) comb. nov.

*Coptoborus inornatus* Wood, 2007

*Coptoborus janeway* sp. nov.

*Coptoborus katniss* sp. nov.

*Coptoborus leeloo* sp. nov.

*Coptoborus leia* sp. nov.

*Coptoborus leporinus* sp. nov.

*Coptoborus magnus* (Petrov, 2020) comb. nov.

*Coptoborus martinezae* sp. nov.

*Coptoborus micarius* (Wood, 1974) comb. nov.

*Coptoborus murinus* sp. nov.

*Coptoborus newt* sp. nov.

*Coptoborus nudulus* Wood, 2007

*Coptoborus obtusicornis* (Schedl, 1976) comb. nov.

*Coptoborus ochromactonus* Smith & Cognato, 2014

*Coptoborus osbornae* sp. nov.

*Coptoborus panosus* sp. nov.

*Coptoborus papillicauda* sp. nov.

*Coptoborus paurus* (Wood, 2007) comb. nov.

*Coptoborus pilisoror* sp. nov.

*Coptoborus pristis* (Wood, 1974) comb. nov.

*Coptoborus pseudotenuis* (Schedl, 1936) comb. nov.

*Xyleborus tenuis* Schedl, 1948

*Coptoborus puertoricensis* (Bright & Torres, 2006) comb. nov.

*Coptoborus ricini* (Eggers, 1932) comb. nov.

*Xyleborus solitariceps* Schedl, 1954

*Coptoborus ripley* sp. nov.

*Coptoborus sagitticauda* sp. nov.

*Coptoborus sarahconnor* sp. nov.

*Coptoborus schulzi* Wood, 2007

*Coptoborus scully* sp. nov.

*Coptoborus semicostatus* (Schedl, 1948) comb. nov.

*Coptoborus sicula* sp. nov.

*Coptoborus silviasilasi* Atkinson, 2018

*Coptoborus solitariformis* (Schedl, 1976)

*Coptoborus sororcula* sp. nov.

*Coptoborus spicatus* Wood, 2007

*Coptoborus starbuck* sp. nov.

*Coptoborus subtilis* (Schedl, 1970)

*Coptoborus tolimanus* (Eggers, 1928)

*Coptoborus trinity* sp. nov.

*Coptoborus tristiculus* (Wood, 1975) comb. nov.

*Coptoborus uhura* sp. nov.

*Coptoborus vasquez* sp. nov.

*Coptoborus vespatorius* (Schedl, 1931)

*Xyleborus emarginatus* Hopkins, 1915

*Xyleborus corniculatus* Schedl, 1948

*Xyleborus corniculatulus* Schedl, 1948

*Coptoborus villosulus* (Blandford, 1898) comb. nov.

*Theoborus theobromae* Hopkins, 1915 syn. nov.

*Xyleborus pseudococcotrypes* Eggers, 1941

*Xyleborus coccotrypoides* Eggers, 1943

*Xyleborus villosus* Schedl, 1948

*Xyleborus hirtellus* Schedl, 1948

*Coptoborus vrataski* sp. nov.

*Coptoborus yar* sp. nov.

#### Removed from *Coptoborus*

*Xyleborus neosphenos* Schedl, 1976 comb. res.

*Xyleborus neosphenos* Schedl, 1976: 76.

*Coptoborus neosphenos* (Schedl): Wood and Bright 1992: 663.

**Type material. *Holotype*** (NHMW), examined.

**Remarks.** This species is removed from *Coptoborus* because of its incongruent morphology which includes a type 1 antennal club with segment 1 encircling the anterior face, lack of sutures on the posterior face, and very slender protibiae. It is transferred to *Xyleborus* until additional investigations can correctly place it in a genus.

### Taxonomic treatment

#### Coptoborus

Taxon classificationAnimaliaColeopteraCurculionidae

Hopkins, 1915

16C83EC5-647E-5FF0-9F72-EC256DB4FFB0

##### Type species.

*Xyleborus vespatorius* Schedl, 1931; original designation.

##### Diagnosis.

*Coptoborus* is distinguished from all other Xyleborini genera by the following combination of characters: antennal funicle four-segmented, antennal club type 3, 4 or 2 (typically type 3) with two (rarely three) arcuate sutures visible on the posterior face, club round or longer than wide, posterior face of the protibiae flat and unarmed, both elytral discal striae and interstriae uniseriate punctate, anterior margin of pronotum typically weakly produced with a row of serrations, pronotal disc alutaceous, procoxae contiguous, scutellum small, flush with elytral surface and mycangial tufts absent.

*Coptoborus* is very similar to *Euwallacea* Hopkins, 1915, and like *Euwallacea*, is diagnosed by a combination of homoplastic characters (Smith et al. 2019a, 2020). Both genera have the posterior face of the antennal club with 2 or 3 arcuate sutures near the apex. *Euwallacea* species typically have a subquadrate or quadrate pronotum (types 3, 4, 8) but some species do have rounded anterior margins like *Coptoborus* (types 2, 7). All *Coptoborus* have rounded anterior margins of the pronotum (types 7, 2, 1 or 9) (except *C. obtusicornis* (type b) which is conspicuously elongate and acuminate frontally), and most have the median area of the pronotum weakly produced and bearing a row of serrations, usually 2–6. *Euwallacea* species with rounded anterior margins of the pronotum always lack serrations, have semi-circular protibiae with evenly rounded outer edge (except obliquely triangular in *E. luctuosus* (Eggers, 1939)) and a posterolateral costa that extends to at interstriae 7 (except inconspicuous, short in *E. luctuous*).

##### Revised description.

**Female.** Length 1.4–3.6 mm and 2.1–4.3 × as long as wide. Body nearly glabrous to densely setose; color variable, light to dark brown, red brown to nearly black; legs and antennae yellow brown to red brown. Appearing very stout to slender, elytra rather variable, appearing round, attenuate, or acuminate. Mycangial tufts absent.

***Head*:** Epistoma entire, transverse, lined with a row of hair-like setae. Frons slightly convex from epistoma to upper level of eyes; surface shagreened, dull, reticulate; punctures small, fine, shallow. Eyes broadly or narrowly emarginate above level of antennal insertion, upper portion of eyes smaller than lower part. Submentum slightly or deeply impressed below genae, narrowly or broadly triangular. Scape short and thick or long and thick, about as long as club. Antennal funicle four-segmented, segments equal in size. Pedicle shorter than funicle or as long as funicle. Club variable, either obliquely truncate, type 2, approximately circular, segment 1 corneous, transverse or weakly convex on anterior face, nearly covering all of posterior face; segment 2 slightly procurved, corneous, visible on anterior and posterior faces of club or club flattened, types 3 or 4 (rarely obliquely truncate and type 2), approximately circular or longer than wide, segment 1 corneous, transverse or sinuate on anterior face, with segments 1, 2, and rarely 3 visible on posterior face. ***Pronotum***: 0.8–1.75 × as long as wide. Pronotum from lateral view typically elongate with disc as long or slightly longer than anterior slope (type 7), taller than basic (type 2), or round (type 1), rarely basic (type 0) or elongate with disc much longer than anterior slope (type 8). In dorsal view typically rounded frontally and long (type 7), basic and parallel-sided (type 2), rarely rounded (type 1) or rounded frontally and very long (type 9) or conspicuously elongate and angulate frontally (type b), anterior margin of pronotum typically weakly produced with a row of 2–6 serrations. Surface alutaceous, anterior slope finely asperate, asperities close, arranged in concentric rings from midpoint of pronotum to anterior and anterolateral areas; disc finely and evenly punctate. Lateral margins variable, obliquely costate, carinate on basal third or along entire length. Posterior angles acutely rounded. Base transverse. ***Elytra***: 1.2–2.5 × as long as wide. Elytral base transverse, margin oblique; humeral angles rounded. Scutellum small to minute, triangular or linguiform, flat, flush with elytra. Elytral shape quite variable, sides straight between basal 42–88%. Disc convex, longer than declivity, rarely as long as declivity. Disc smooth, shiny, finely punctate; striae not impressed, interstrial punctures seriate, or confused (rare). Declivity variable. Posterolateral margin of declivity typically with interstriae 3 and 9 joining, forming a carina and continuing submarginally to apex, but may also be costa, or carinate from suture to interstriae 2, 7, or 8. ***Legs***: Procoxae contiguous. Protibiae obliquely triangular, broadest at apical third, or distinctly triangular, or with evenly rounded outer margin, posterior face flat, unarmed; 5–8 denticles present on outer margin. Meso- and metatibiae obliquely triangular, flattened, posterior face unarmed with 6–12 and 6–11 denticles, respectively.

### Key to *Coptoborus species* (females only) (excluding *C. artetenuis*)

**Table:** 

1	Elytral apex broadly rounded, never emarginate (Fig. 19A, B, J)	**2**
–	Elytral apex prolonged apically, attenuate (Fig. 18J, P) or acuminate (Fig. 9A, M), emarginate in some species (Fig. 18J, P)	**15**
2	Posterolateral margin of declivity unarmed by a carina	**3**
–	Posterolateral margin of declivity carinate, carina variable in length from very short and mostly visible to striae 2 or distinct and very long, extending to at least striae 6	**6**
3	Declivity convex, interstriae not impressed; discal interstrial punctures confused; larger species, 1.7–2.2 mm	***C. villosulus***
–	Declivity with impressed interstriae or interstriae 2 sulcate; discal interstrial punctures uniseriate; smaller species, 1.4–1.7 mm	**4**
4	Anterior margin of pronotum without a row of serrations; declivital interstrial setae about the combined width of striae 1 and interstriae 1	***C. erwini* sp. nov.**
–	Anterior margin of pronotum armed by two projecting serrations; declivital interstrial setae much longer than the combined width of striae 1 and interstriae 1	**5**
5	Declivital interstriae weakly impressed; all interstriae uniformly armed by granules along their length	***C. doliolum* sp. nov.**
–	Declivital interstriae 2 sulcate; interstriae 1 granulate, interstriae 3 denticulate (those larger than interstriae 1 granules), interstriae 2 with a staggered row of minute obscure granules	***C. paurus***
6	Posterolateral declivital carina smooth, continuous along its length (Fig. 4G, O)	**7**
–	Posterolateral declivital carina serrate, appearing broken (Fig. 13D, N)	**11**
7	Posterolateral carina conspicuously extended posteriad, appearing shelf-like (Fig. 14A, M); declivity moderately impressed along interstriae 2	***C. ricini***
–	Posterolateral carina not extended posteriad (Fig. 4G, O); declivity either with interstriae 2 flattened or convex, or broadly and shallowly impressed between interstriae 3	**8**
8	Declivity broadly and shallowly impressed between interstriae 3; larger, 2.7–2.9 mm	***C. coartatus***
–	Declivity either with interstriae 2 flattened or convex; smaller, 1.7–2.3 mm	**9**
9	Declivital interstriae setae stout, scale-like; declivital striae not impressed, striae and interstriae flush; smaller, 1.7–1.8 mm and more elongate species, 2.6 × as long as wide (Fig. 3A, M)	***C. brigman* sp. nov.**
–	Declivital interstriae setae fine, hair-like; declivital striae 1 and 2 impressed; larger 2.2–2.4 mm, and stouter species, 2.2–2.3 × as long as wide (Fig. 18A, M)	**10**
10	Declivital interstriae feebly granulate, granules sparse, minute, indistinct; declivital face flattened; striae 1 and 2 feebly impressed; declivity moderately covered with hair-like setae shorter than the width of interstriae 2 (Fig. 9G, O)	***C. leia* sp. nov.**
–	Declivital interstriae moderately granulate, granules large, distinct; striae 1 and 2 distinctly impressed; declivity abundantly covered with hair-like setae longer than the width of interstriae 2 (Fig. 18A, M)	***C. tristiculus***
11	Posterolateral carina faint, primarily visible between suture and striae 2 (Fig. 15A, M)	**12**
–	Posterolateral carina distinct, clearly visible from suture to at least striae 6 (Figs 5N, 13N)	**13**
12	Declivity gradual, occupying posterior half of elytra; declivital interstrial setae twice as long as the width of interstriae 1; larger and stouter species, 2.2 mm, 2.4 × as long as wide	***C. murinus* sp. nov**.
–	Declivity very steep, occupying posterior quarter of elytra; declivital interstrial setae as long as interstriae 1 width; smaller and more elongate species, 1.5–1.7 mm, 2.7–3.0 × as long as wide	***C. osbornae* sp. nov.**
13	Posterolateral carina serrations equally sized along its length; elytra stout, 1.3 × as long as wide	***C. crinitulus***
–	Posterolateral carina serrations unequally sized along its length; elytra elongate, 1.6–1.9 × as long as wide	**14**
14	Posterolateral carina with serrations on interstriae 1 and 2 subquadrate, at least twice as large as other serrations (Fig. 13D, N)	***C. pristis***
–	Posterolateral carina with serrations on interstriae 1 and 2 with acute apices, less than twice the size of other serrations (Fig. 1OG, O)	***C. micarius***
15	Declivity excavated, broadly and deeply sulcate between interstriae 3	**16**
–	Declivity either convex, sulcate only along interstriae 2 or weakly sulcate between interstriae 3	**20**
16	Stout species, 2.1–2.5 × as long as wide	**17**
–	Slender species, 3.2–4.3 × as long as wide	**19**
17	Declivital interstriae 1 with a large digitate projection, with length ~2 × its basal diameter and a large digitate projection at the middle of the declivity on interstriae 3 (Fig. 16A, L); declivital slope gradual; color dark brown or black	***C. silviasilasi***
–	Declivital interstriae never with digitate projections; declivital slope obliquely truncate; color light brown	**18**
18	Larger and stouter species, 3.1 mm, 2.17 × as long as wide; disc occupying 80% of elytral length; declivity strongly impressed on basal half; short setae on elytral disc	***C. magnus***
–	Smaller and more elongate, 2.8 mm, 2.37 × as long as wide; disc occupying 65% of elytral length; declivity weakly impressed along entire length; long setae on elytral disc	***C. amazonicus***
19	Anterior margin of pronotum bearing two projecting serrations; sulcate area bearing small granules or denticles; more elongate species, 3.8–4.3 × as long as wide	***C. obtusicornis***
–	Anterior margin of pronotum without two projecting serrations; sulcate area unarmed; less elongate species, 3.2–3.5 × as long as wide	***C. vespatorius***
20	Declivital interstriae 2 armed, bearing granules or denticles (some may be small) (excluding elytral apex)	**21**
–	Declivital interstriae 2 unarmed along the entire length, entirely devoid of granules or denticles (excluding elytral apex)	**59**
21	Elytral apex with a long continuous elevated carina along sutural margin to interstriae 7 (Fig. 17A, M)	**22**
–	Elytral apex never with a carina that extends to interstriae 7, carina may be short, extending to the convergence of interstriae 3 and 9 (Fig. 7J, P), only along interstriae 8 on acuminate elytral apices (Fig. 19G, L), or poorly defined and costate (Fig. 8A, M)	**25**
22	Declivital interstriae raised, forming vermiculate ridges (Fig. 17A, M)	**23**
–	Declivital interstriae not raised, granulate, without vermiculate ridges (Fig. 19D, K)	**24**
23	Vermiculate ridges shorter, as high as 2 × strial width; declivity subshiny; smaller, 3.1 mm (Fig. 15G, O)	***C. semicostatus***
–	Vermiculate ridges taller, as high as 4 × strial width; declivity shagreened; larger, 3.6 mm (Fig. 17A, M)	***C. starbuck* sp. nov.**
24	Declivital interstriae 2 deeply sulcate; declivital interstriae densely granulate, granules on interstriae 1 and 3 separated by the distance of a granule; declivital interstriae densely covered with long thick erect scale-like setae	***C. vrataski* sp. nov.**
–	Declivital interstriae 2 flush with surface; declivital interstriae sparsely granulate, granules on interstriae 1 and 3 separated by the distance of three granules; declivital interstriae moderately covered with long erect hair-like setae	***C. panosus* sp. nov.**
25	Posterolateral margin of elytra with interstriae 3 and 9 joining, forming a short but distinct carina that continues submarginally to apex (Fig. 7J, P)	**26**
–	Posterolateral margin of elytra weakly costate and granulate/denticulate (Fig. 8A, M), or apex acuminate (Fig. 19G, L)	**51**
26	Elytral apex entire	**27**
–	Elytral apex weakly to strongly emarginate	**34**
27	Declivital interstriae densely and coarsely denticulate, denticles large, very closely spaced	***C. trinity* sp. nov.**
–	Declivital interstriae granulate or finely denticulate, granules or denticles small, widely spaced	**28**
28	Elytral apex carina apically produced, apical projection the width of striae 2 (Fig. 2J, P)	**29**
–	Elytral apex not apically produced (Fig. 2A, M)	**30**
29	Declivital interstriae 1–3 denticles distinct, their height equal to interstriae width	***C. gentilis***
–	Declivital interstriae 1–3 denticles minute, faint, their height less than 0.5 × interstriae width	***C. brevicauda* sp. nov.**
30	Declivity weakly convex	***C. barbicauda* sp. nov.**
–	Declivity feebly to weakly sulcate	**31**
31	Declivity weakly but distinctly sulcate	***C. uhura* sp. nov.**
–	Declivity feebly sulcate	**32**
32	Declivital interstriae 1 and 3 denticles large, distinct; declivital interstriae with sparse bristle-like setae	***C. subtilis***
–	Declivital interstriae 1 and 3 denticles small, relatively indistinct; declivital interstriae and striae densely covered with abundant hair-like setae	**33**
33	Declivital interstrial setae 2–3 × as long as interstriae 1 width, setae uniformly fine from base to apex; declivital interstriae 1 unarmed on apical half. (Fig. 7J, P)	***C. hansen* sp. nov.**
–	Declivital interstrial setae 1–1.5 × as long as interstriae 1 width, setae becoming thicker from base to apex; declivital interstriae 1 unarmed on apical quarter (Fig. 3G, O)	***C. capillisoror* sp. nov.**
34	Elytral apex weakly emarginate (Fig. 15A)	**35**
–	Elytral apex strongly emarginate (Figs 6J, 13D)	**42**
35	Declivital interstriae 2 convex	**36**
–	Declivital interstriae 2 distinctly impressed	**37**
36	Declivital interstriae 2 denticles minute, distinctly smaller than those of interstriae 1 or 3; declivity reticulate, shagreened, dull; larger, 2.2–2.3 mm	***C. puertoricensis***
–	Declivital interstriae 2 denticles distinct, about as large as those of interstriae 1 and 3; declivity smooth, shiny; smaller, 1.9 mm	***C. solitariformis***
37	Anterior margin of pronotum with a pair of projecting serrations	**38**
–	Anterior margin of pronotum without a pair of projecting serrations	**40**
38	Declivital interstriae 2 weakly impressed with a median row of minute granules	***C. atlanticus***
–	Declivital interstriae 2 moderately or strongly impressed with denticles on the basal third or entire length	**39**
39	Declivital interstriae 2 moderately impressed; smaller, 1.8–1.9 mm	***C. crassisororcula* sp. nov.**
–	Declivital interstriae 2 strongly impressed; larger, 2.3 mm	***C. incultus***
40	More slender, 3–3.4 × as long as wide; denticles on declivital interstriae 1 and 3 large, distinct	***C. pseudotenuis***
–	Stouter, 2.6–2.7 × as long as wide; denticles on declivital interstriae 1 and 3 small, difficult to discern	**41**
41	Declivital interstriae 1 with a confused row of erect scale-like setae; posterior ~40% of elytra acutely tapered to apex (Fig. 15A)	***C. schulzi***
–	Declivital interstriae 1 with a uniseriate row of erect scale-like setae; posterior ~40% of elytra gradually tapered to apex (Fig. 2G)	***C. bettysmithae* sp. nov.**
42	Elytral apex carina crenulate	**43**
–	Elytral apex carina continuous, smooth	**48**
43	Elytral declivital interstriae 3 with ten or more denticles	***C. furiosa* sp. nov.**
–	Elytral declivital interstriae 3 with fewer than ten denticles	**44**
44	Elytral apex crenulations of equal size	***C. asperatus* sp. nov.**
–	Elytral apex crenulation next to suture larger than other crenulations	**45**
45	Declivital striae 1–3 impressed; stouter, 2.5 × as long as wide	***C. carumbensis***
–	Declivital striae 1–3 not impressed; more elongate, 2.8–3.4 × as long as wide	**46**
46	Elytra stouter, 1.6 × as long as wide	***C. inornatus***
–	Elytra more elongate, 1.7–2.0 × as long as wide	**47**
47	Elytral apex crenulations smaller; declivital slope more gradual, declivity occupying ~57% of elytra (Fig. 8K)	***C. janeway* sp. nov.**
–	Elytral apex crenulations larger; declivital slope steeper, declivity occupying ~50% of elytra (Fig. 17H)	***C. tolimanus***
48	Declivity obtusely tapered, steeply descending; elytral apex not produced (Fig. 10D)	***C. martinezae* sp. nov.**
–	Declivity acutely tapered, gradually descending; apex distinctly produced (Fig. 7G)	**49**
49	Declivital interstriae 1 and 3 denticles subequal	***C. gracilens***
–	Declivital interstriae 3 denticles much larger than those of interstriae 1	**50**
50	Interstriae with long, erect hair-like setae at least twice as wide as interstrial width; larger, 2.35 mm	***C. leporinus* sp. nov.**
–	Interstriae with erect bristle-like setae shorter than interstrial width; smaller, 1.8–2.0 mm	***C. cracens***
51	Elytral apex acuminate (Fig. 14G)	**52**
–	Elytral apex rounded, never acuminate (Fig. 8A)	**55**
52	Declivity with a posterolateral costa extending from apex to interstriae 8 (Fig. 14G)	**53**
–	Declivity either with a very short carina on posterolateral margin extending from apex to interstriae 2 or without a posterolateral costa	**54**
53	Pronotum 1.1 × as long as wide; smaller, 2.0 mm, 2.5 × as long as wide (Fig. 1J)	***C. attenuatus***
–	Pronotum 1.25 × as long as wide; larger, 2.3 mm, 2.88 × as long as wide (Fig. 14G)	***C. sagitticauda* sp. nov.**
54	Elytral discal interstriae 2 with two rows of confused punctures; posterolateral margin with a very short carina extending from apex to interstriae 2; larger, 2.8–2.9 mm	***C. yar* sp. nov.**
–	Elytral discal interstriae 2 with uniseriate punctures; posterolateral margin without a costa or carina; smaller, 2.1 mm	***C. sicula* sp. nov.**
55	Declivital interstriae 2 with about as many denticles as interstriae 1 or 3	**56**
–	Declivital interstriae 2 with much fewer denticles than interstriae 1 or 3	**57**
56	Declivital interstriae 2 denticles as large as those of interstriae 1; larger 1.7–2.0 mm; declivity steep (Fig. 15D, N)	***C. scully* sp. nov.**
–	Declivital interstriae 2 denticles smaller than those of interstriae 1; smaller, 1.7 mm; declivity more gradual (Fig. 1A, M)	***C. newt* sp. nov.**
57	Elytral apex weakly emarginate	***C. catulus***
–	Elytral apex entire	**58**
58	Smaller, 1.7–1.9 mm; antennal club obliquely truncate, type 2, segment 1 occupying basal 1/2	***C. incomptus* sp. nov.**
–	Larger, 2.8 mm; antennal club flat, type 3, segment 1 occupying basal 1/4	***C. amplissimus* sp. nov.**
59	Declivital interstriae 2 sulcate (Fig. 14D, N)	**60**
–	Declivital interstriae 2 convex (Fig. 14J, P)	**68**
60	Declivital interstriae 1 and 3 unarmed, devoid of granules or denticles (Fig. 11D, N)	**61**
–	Declivital interstriae 1 and 3 armed, bearing granules or denticles (Fig. 14D, N)	**63**
61	Declivity densely covered with thick recumbent setae; smaller, 1.8 mm	***C. pilisoror* sp. nov.**
–	Declivity glabrous, larger, 2.2–2.4 mm	**62**
62	Declivital interstriae deeply impressed between suture and interstriae 3, interstriae 3 clearly elevated and costate; declivity smooth, shiny	***C. nudulus***
–	Declivital interstriae shallowly impressed between suture and interstriae 3, interstriae 3 feebly elevated; declivity shagreened, dull	***C. sororcula* sp. nov.**
63	Declivity moderately to strongly sulcate along interstriae 2, interstriae 3 strongly elevated (Fig. 14D, N)	**64**
–	Declivity weakly sulcate along interstriae 2, interstriae 3 weakly elevated (Fig. 16I, O)	**67**
64	Declivital striae 1 and 2 not parallel on declivital face, nearly converging in sulcate area; smaller, 1.6–1.7 mm	***C. leeloo* sp. nov.**
–	Declivital striae 1 and 2 parallel on declivital face, widely spaced; larger, 2.5–3.5 mm	**65**
65	Declivital interstriae 2 impunctate; larger, 3.5 mm	***C. ripley* sp. nov.**
–	Declivital interstriae 2 punctate, numerous distinct punctures on basal half and several minute punctures on posterior half; smaller, 2.5–2.7 mm	**66**
66	Smaller, 2.5–2.6 mm, less elongate, 2.5–2.6 × as long as wide; pronotum stouter, 1.05–1.1 × as long as wide; distributed west of the Andes	***C. ochromactonus***
–	Larger, 2.7 mm, more elongate, 2.7 × as long as wide; pronotum more elongate, 1.2 × as long as wide; distributed east of the Andes	***C. busoror* sp. nov.**
67	Declivity nearly glabrous; declivital interstriae 1 and 3 with six and four small to moderate denticles, respectively; body light brown; stouter, body 2.4 × as long as wide, elytra 1.4 × as long as wide	***C. spicatus***
–	Declivity densely setose; declivital interstriae 1 and 3 with two large denticles; body dark brown; more elongate, body 3 × as long as wide, elytra 2 × as long as wide	***C. vasquez* sp. nov.**
68	Elytral apex acuminate	**69**
–	Elytral apex entire or emarginate	**71**
69	Elytral apex feebly acuminate (Fig. 2D)	***C. bellus***
–	Elytral apex strongly acuminate (Fig. 9A)	**70**
70	Elytral discal interstriae punctate, declivity with a carina extending from apex to interstriae 2; smaller, 2.3 mm (Fig. 14J, P)	***C. sarahconnor* sp. nov.**
–	Elytral discal interstriae impunctate, declivity with a carina extending from apex to interstriae 3; larger, 2.7 mm (Fig. 9A, M)	***C. katniss* sp. nov.**
71	Elytral apex obviously emarginate	**72**
–	Elytral apex weakly emarginate or entire	**74**
72	Declivity more gradual, occupying at least posterior 50% of elytral length (Fig. 4B)	***C. exilis***
–	Declivity steeper, occupying less than posterior 40% of declivity (Fig. 5H)	**73**
73	Declivity devoid of denticles or tubercles on interstriae 1–3	***C. exutus***
–	Declivity with denticles on interstriae 1 and 3	***C. cuneatus***
74	Elytral apex weakly emarginate (Fig. 7M); declivital striae shallowly impressed	***C. galacatosae* sp. nov.**
–	Elytral apex entire (Fig. 12O); declivital striae deeply impressed	**75**
75	Denticles on declivital interstriae 1 large, 1–2 × high as wide (Fig. 12G)	***C. papillicauda* sp. nov.**
–	Denticles on declivital interstriae 1 small, 0.5–1 × high as wide (Fig. 4D)	***C. chica* sp. nov.**

#### Coptoborus amazonicus

Taxon classificationAnimaliaColeopteraCurculionidae

(Petrov, 2020) comb. nov.

28D8B1AD-50BE-552C-A03A-498F85A699ED

Figure 1A–C, M

Theoborus amazonicus Petrov, 2020: 406.

##### Type material.

***Holotype*** (ZMMU), not examined, ***paratype*** (ZMMU), examined.

##### New records.

Peru: Junin, near Rio Venado village, 1100 m a.s.l., Petrov (APP, 3).

##### Diagnosis.

2.8 mm (n = 1), 2.37 × as long as wide (Petrov 2020). This species is distinguished by the elytra attenuated, apex entire, elytra shallowly excavated between interstriae 3, anterior margin of pronotum with a pair of projecting serrations, disc occupying 65% of elytral length, moderately sized at 2.8 mm, and stout, 2.37 × as long as wide.

##### Similar species.

*C. magnus*.

##### Distribution.

Peru (Junin, Loreto).

##### Biology.

Unknown.

**Figure 1.**
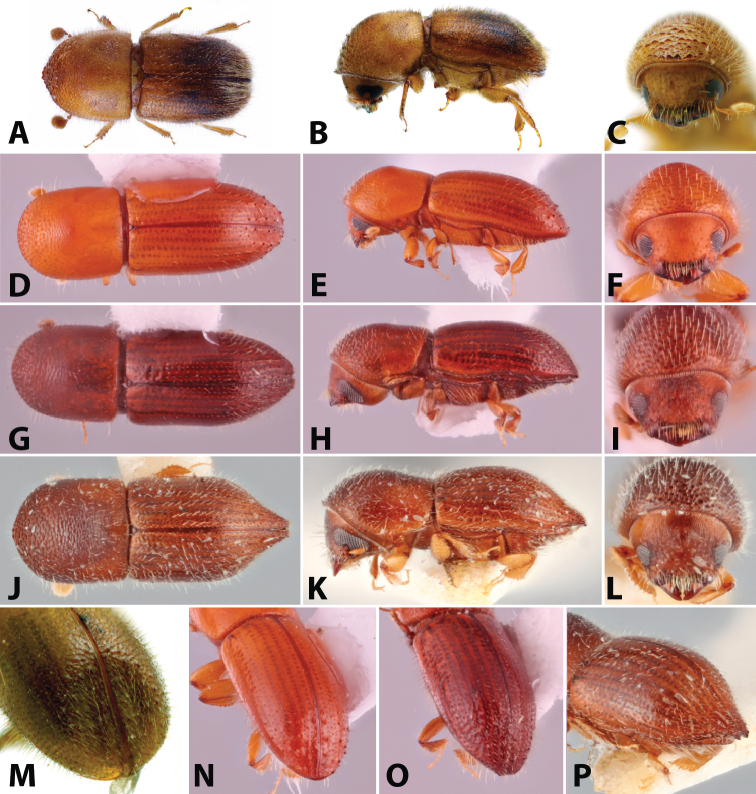
Dorsal, lateral, frontal and declivital view of *Coptoborus amazonicus* holotype, 2.8 mm (**A–C, M**), *C. amplissimus* holotype, 2.8 mm (**D–F, N**), *C. asperatus* holotype, 2.0 mm (**G–I, O**), *C. attenuatus* holotype, 2.0 mm (**J–L, P**). All photographs by SMS except (**A–C, M**) by A.V. Petrov and (**J–L, P**) by R.K. Osborn.

#### Coptoborus amplissimus sp. nov.

Taxon classificationAnimaliaColeopteraCurculionidae

B7109BB4-ECAB-567F-8A05-3C4A897AF8F2

http://zoobank.org/6F25B756-A846-4E36-9923-04B6CA4855D1

Figure 1D–F, N

##### Type material.

***Holotype*,** female, Peru: Madre de Dios, Los Amigos Biological Station, CM2, 12°44.92'S, 70°25.17'W, 17–18.v.2008, Smith, Hulcr, sample Peru 103a, 8 cm diameter branch (MUSM).

##### Diagnosis.

2.8 mm (n = 1), 2.8 × as long as wide. This species is distinguished by the elytral apex attenuate and entire, declivital interstriae 1–3 denticulate, interstriae 2 with much fewer denticles than interstriae 1 or 3, antennal club flat, type 3, segment 1 occupying basal 1/4, and posterolateral margin of declivity costate, armed with two denticles.

##### Similar species.

*C. catulus*, *C. incomptus*, *C. newt*, *C. scully*.

##### Description

**(female). *Holotype*** 2.8 mm, 2.8 × as long as wide. Body light brown, elytral declivity darker, antennae and legs lighter. ***Head***: epistoma smooth. Frons dull, finely punctate, glabrous. Eyes broadly and moderately emarginate. Submentum narrow, triangular, deeply impressed. Antennal scape short and thick, as long as club. Pedicel shorter than funicle. Club circular, flat, type 3; segment 1 corneous, subconvex on anterior face, occupying basal ~1/4; segment 2 narrow, subconvex, corneous; segments 1 and 2 present on posterior face. ***Pronotum***: 1.2 × as long as wide. In dorsal view long and rounded frontally, type 7, sides parallel in basal 3/4, rounded anteriorly; anterior margin without serrations. In lateral view elongate, disc as long as anterior slope, type 7, summit prominent, at midpoint. Anterior slope with densely spaced, broad fine asperities, becoming lower and more strongly transverse towards summit. Disc strongly shiny with sparse, minute punctures, some longer hair-like setae at margins. Lateral margins obliquely costate. ***Elytra***: 1.6 × as long as wide, 1.3 × as long as pronotum. Scutellum minute. Elytra attenuate, parallel-sided in basal 63%, then acutely tapered to apex, apex entire. Disc smooth, shiny; striae minutely punctate, glabrous; interstriae flat, sparsely, minutely punctate, unarmed, each puncture bearing a long, erect seta. Declivity gradually rounded, occupying ~1/3 of elytra, smooth, shiny, declivital face weakly flattened; striae not impressed, strial punctures larger, deeper than those of disc, each puncture bearing a recumbent seta as long as one punctures, striae 1 slightly laterally broadened from base to declivital midpoint and then narrowing towards apex; interstriae flat, interstriae 1 and 3 with four or five and four, respectively, subequal, uniformly spaced small denticles, interstriae 2 with a row of minute denticles, interstriae 2 with five or six minute denticles, much smaller than those of interstriae 1 or 3, interstrial setae sparse erect bristle-like, interstriae 1 with an additional sparse row of slightly shorter semi-recumbent setae. Posterolateral margin costate, armed with two denticles. ***Legs***: protibiae distinctly triangular, broadest at apical 1/5; apical 1/2 of outer margin with six large, socketed denticles, their length longer than basal width. Meso- and metatibiae flattened; outer margin evenly rounded with seven and eight moderately sized socketed denticles, respectively, their lengths equal to basal width.

##### Etymology.

*L. amplissimus* = largest. In reference to the species size relative to other similar species. Adjective.

##### Distribution.

Peru (Madre de Dios).

##### Biology.

This species was collected from an unidentified branch 8 cm in diameter.

#### Coptoborus artetenuis

Taxon classificationAnimaliaColeopteraCurculionidae

(Schedl, 1973)

62116BFF-A89D-59C8-8F7F-95DCD3B468BE

Xyleborus artetenuis Schedl, 1973: 372.
Coptoborus artetenuis (Schedl): Wood and Bright 1992: 662.

##### Type material.

***Holotype*** (NHMB), not examined and potentially lost (see remarks).

##### New records.

None.

##### Diagnosis.

1.85 mm, 2.9 × as long as wide (Schedl 1973). This species is distinguished by the elytral apex attenuate and weakly emarginate, declivital interstriae 2 convex, declivital interstriae 1 and 3 denticulate and interstriae 2 unarmed, elytral apex deeply emarginate, declivity gradual, occupying at least posterior 50% of declivity, 1.85 mm and 2.9 × as long as wide. This species is most similar to *C. exilis* which lacks granules or denticles on declivital interstriae 2.

##### Similar species.

*C. exilis*, *C. pseudotenuis*.

##### Distribution.

Bolivia (Beni).

##### Biology.

Unknown.

##### Remarks.

Schedl (1973) stated that the holotype was deposited in NHMB. The specimen is not there, nor in Schedl’s collection in NHMW (Schedl 1979; Wood 2007). Wood (2007) reported it from MZUSP but this is not confirmed.

#### Coptoborus asperatus sp. nov.

Taxon classificationAnimaliaColeopteraCurculionidae

7A33407C-5736-5418-B684-46B5FA164830

http://zoobank.org/E2AA8F62-3AEA-4398-9570-6151576AE9DD

Figure 1G–I, O

##### Type material.

***Holotype*,** female, Ecuador: Napo Prov. [= Orellana Prov.], Res[erva]. Ethnica Waorani, 1 km S. Okone Gare Camp, Trans[ect]. Ent[omology]., 00°39'10"S, 076°26'W, 220 m, October 1994, T.L. Erwin et al. collectors, insecticidal fogging, terra firme forest, trans[ect] 6, sta[tion] 3, Erwin lot #922 (ICB).

##### Diagnosis.

2.0 mm (n = 1), 2.86 × as long as wide. This species is distinguished by the elytral apex attenuate and strongly emarginate, declivity convex, declivital interstriae 2 denticulate, elytral apex with interstriae 3 and 9 joining, forming a crenulate carina that continues submarginally to apex, crenulations equal in size, declivital interstriae 3 densely denticulate with fewer than ten denticles, declivital interstriae 1 with three rows of setae, and declivital striae not impressed.

##### Similar species.

*C. carumbensis*.

##### Description

**(female). *Holotype*** 2.0 mm, 2.86 × as long as wide. Body uniformly brown, antennae and legs lighter. ***Head***: epistoma tuberculate. Frons dull, finely punctate, glabrous. Eyes broadly and moderately emarginate. Submentum narrow, triangular, slightly impressed. Antennal scape short and thick, much shorter than club. Pedicel shorter than funicle. Club circular, flat, type 3; segment 1 corneous, procurved on anterior face, occupying basal ~1/3; segment 2 narrow, subconvex, corneous; segments 1 and 2 present on posterior face. ***Pronotum***: 1.1 × as long as wide. In dorsal view long and rounded frontally, type 7, sides parallel in basal 2/3, rounded anteriorly; anterior margin without serrations. In lateral view elongate, disc as long as anterior slope, type 7, summit indistinct, at midpoint. Anterior slope with densely spaced, broad fine asperities, becoming lower and more strongly transverse towards summit. Disc strongly shiny with moderately dense, minute punctures, some longer hair-like setae at margins. Lateral margins obliquely costate. ***Elytra***: 1.7 × as long as wide, 1.5 × as long as pronotum. Scutellum small. Elytra attenuate, parallel-sided in basal 2/3, then acutely tapered to apex, apex weakly emarginate. Disc smooth, shiny; strial punctures large, deep, each bearing a recumbent seta the length of a puncture; interstriae flat, sparsely, minutely punctate, unarmed, each puncture bearing a long, erect seta. Declivity gradual, occupying ~1/3 of elytra, shagreened, dull, declivital face convex; striae not impressed, strial punctures larger, deeper than those of disc, each puncture bearing a semi-erect seta as long as two punctures; interstriae flat, interstriae denticulate along their entire lengths, interstriae 3 sparsely denticulate, denticles separated by at least the width of three denticles and with eight or fewer denticles, interstrial setae erect, bristle-like, uniseriate, interstriae 1 with two additional rows of slightly shorter erect hair-like setae. Posterolateral margin with interstriae 3 and 9 joining, forming a granulate carina and continuing submarginally to apex. ***Legs***: protibiae obliquely triangular, broadest at apical 1/3; apical 1/2 of outer margin with five moderately sized socketed denticles, their length as long as basal width. Meso- and metatibiae flattened; outer margin evenly rounded with seven and six moderately sized socketed denticles, respectively.

##### Etymology.

L. *asperatus* = rough. In reference to the species’ sculptured declivity. Adjective.

##### Distribution.

Ecuador (Orellana).

##### Biology.

The holotype was collected by canopy fogging.

#### Coptoborus atlanticus

Taxon classificationAnimaliaColeopteraCurculionidae

(Bright & Torres, 2006) comb. nov.

4CA330D7-9865-5586-9DEE-51D5408F7EFD

Xyleborus atlanticus Bright & Torres, 2006: 417.
Theoborus atlanticus (Bright & Torres): Bright 2019: 272.

##### Type material.

***Holotype*,*paratypes*** (CNCI) (Bright 2019), not examined.

##### New records.

None.

##### Diagnosis.

1.8–2.0 mm, 2.7 × as long as wide (Bright and Torres 2006). This species is distinguished by the elytral apex attenuate and weakly emarginate, declivital interstriae 2 granulate along entire length, declivital interstriae 2 weakly impressed, posterolateral margin of declivity with interstriae 3 and 9 joining, forming a carina and continuing submarginally to apex, declivital interstriae distinctly impressed, anterior margin of pronotum with a pair of projecting serrations.

##### Similar species.

*C. crassisororcula*, *C. incultus*.

##### Distribution.

Dominican Republic, Puerto Rico.

##### Biology.

Unknown.

##### Remarks.

Specimens of this species were unable to be examined as part of this study. Our treatment is based on Bright and Torres (2006) description, Bright’s (2019) treatment and images of the declivty.

#### Coptoborus attenuatus

Taxon classificationAnimaliaColeopteraCurculionidae

Wood, 2007

BF4E54BA-9B2C-5A5D-ADDF-8B583FA49BD7

Figure 1J–L, P

Coptoborus attenuatus Wood, 2007: 400.

##### Type material.

***Holotype*** (NHMUK), examined.

##### New records.

None.

##### Diagnosis.

2.0 mm, 2.5 × as long as wide (Wood 2007). This species is distinguished by the elytral apex strongly acuminate, declivital interstriae 2 granulate near apex, declivity with a costa extending from apex to interstriae 8, pronotum 1.1 × as long as wide. It is most similar to *C. sagitticauda* from which it can be distinguished by the smaller size, 2.0 mm vs. 2.3 mm, and stouter form, 2.5 × as long as wide vs. 2.88 × as long as wide.

##### Similar species.

*C. bellus*, *C. katniss*, *C. sagitticauda*, *C. sarahconnor*, *C. sicula*, *C. yar*.

##### Distribution.

Brazil (Mato Grosso).

##### Biology.

Unknown.

##### Remarks.

The holotype has a field notebook code ‘C76’ on its locality label. The holotype was taken at a light trap set 22 m up in a tree in gallery forest (R.A. Beaver, pers. comm., 30 October 2020).

#### Coptoborus barbicauda sp. nov.

Taxon classificationAnimaliaColeopteraCurculionidae

23919FAC-AC42-52FE-809E-E4F822A421F3

http://zoobank.org/042EA1B6-77DB-477A-9F22-88D161BDEEAD

Figure 2A–C, M

##### Type material.

***Holotype*,** female, French Guiana: Amazone Nature Lodge, 30 km SE Roura on Kaw Rd., 18–23-IV-2007, J.E. Eger, 4.55954°N, -52.2072°W, 300 m, UV light trap (NMNH).

##### Diagnosis.

2.0 mm (n = 1), 2.22 × as long as wide. This species is distinguished by the elytral apex attenuate and entire and not produced, declivital interstriae 2 convex, declivital interstriae 1–3 denticulate, posterolateral margin of declivity with interstriae 3 and 9 joining, forming a carina and continuing submarginally to apex, stout form, declivity weakly convex, and declivital interstriae 1 with two rows of erect scale-like setae.

##### Similar species.

*C. bettysmithae*, *C. capillisoror*, *C. hansen*, *C. schulzi*, *C. subtilis*, *C. trinity*, *C. uhura*.

##### Description

**(female). *Holotype*** 2.0 mm, 2.22 × as long as wide. Body uniformly light brown, antennae and legs lighter. ***Head***: epistoma smooth. Frons dull, finely punctate, setose; each puncture bearing a long, erect hair-like seta. Eyes broadly and moderately emarginate. Submentum narrow, triangular, slightly impressed. Antennal scape short and thick, much shorter than club. Pedicel shorter than funicle. Club circular, flat, type 3; segment 1 corneous, subconvex on anterior face, occupying basal ~1/3; segment 2 broad, convex, corneous; segments 1 and 2 present on posterior face. ***Pronotum***: 0.9 × as long as wide. In dorsal view basic and parallel-sided, type 2, sides parallel in basal 2/5, rounded anteriorly; anterior margin with two serrations. In lateral view tall, type 2, disc flat, summit pronounced, at midpoint. Anterior slope with densely spaced, broad coarse asperities, becoming lower and more strongly transverse towards summit. Disc subshiny with dense, minute punctures, some longer hair-like setae at margins. Lateral margins obliquely costate. ***Elytra***: 1.3 × as long as wide, 1.5 × as long as pronotum. Scutellum minute. Elytra attenuate, parallel-sided in basal 3/4, then acutely rounded to apex, apex entire. Disc smooth, shiny; strial punctures large, deep, each bearing a recumbent seta the length of a puncture; interstriae flat, minutely, moderately punctate, unarmed, each puncture bearing a long, erect bristle-like seta. Declivity steeply rounded, occupying ~1/3 of elytra, shagreened, shiny, declivital face weakly convex; striae not impressed, strial punctures larger, deeper than those of disc, each puncture bearing a recumbent seta as long as two punctures; interstriae flat, uniformly denticulate along their entire lengths, denticles confused, spaced by four widths of a denticle, setae erect, scale-like, as long as interstriae 1 width; interstriae 1 with an additional row of shorter erect scale-like setae. Posterolateral margin with interstriae 3 and 9 joining, forming a granulate carina and continuing submarginally to apex. ***Legs***: protibiae obliquely triangular, broadest at apical 1/4; apical 1/2 of outer margin with five large, socketed denticles, their length longer than basal width. Meso- and metatibiae flattened; outer margin evenly rounded with nine large, socketed denticles.

##### Etymology.

L. *barba* = beard, *caudus* = tail. In reference, to the appearance of unkempt beard stubble (setae and granules) on the declivity. Noun in apposition.

##### Distribution.

French Guiana.

##### Biology.

Unknown.

**Figure 2.**
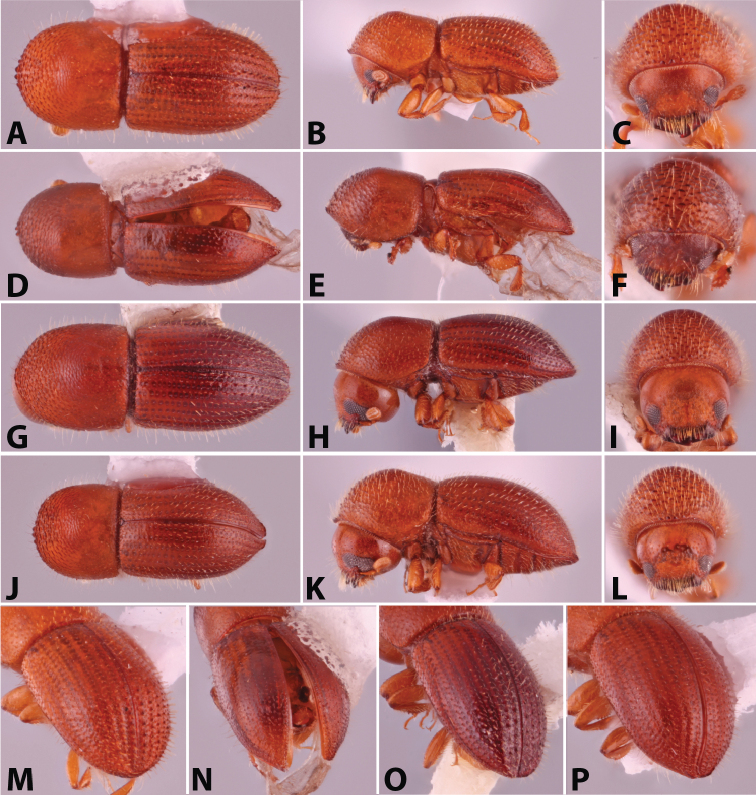
Dorsal, lateral, frontal and declivital view of *Coptoborus barbicauda* holotype, 2.0 mm (**A–C, M**), *C. bellus*, 2.1–2.3 mm (**D–F, N**),*C. bettysmithae* holotype, 2.4 mm (**G–I, O**), *C. brevicauda* holotype, 2.4–2.6 mm (**J–L, P**). All photographs by SMS.

#### Coptoborus bellus

Taxon classificationAnimaliaColeopteraCurculionidae

(Bright & Torres, 2006) comb. nov.

CE97B237-812B-50EB-8C98-1138B8103470

Figure 2D–F, N

Coptoborus bellus Bright & Torres, 2006: 415.
Theoborus bellus Bright & Torres, 2006: Bright 2019: 273.

##### Type material.

***Holotype*** (NMNH), examined.

##### New records.

None.

##### Diagnosis.

2.1–2.3 mm, 2.6 × as long as wide (Bright and Torres 2006). This species is distinguished by the elytral apex feebly acuminate, declivital interstriae unarmed along its entire length, and antennal club with three sutures on posterior face.

##### Similar species.

*C. attenuatus*, *C. katniss*, *C. sagitticauda*, *C. sarahconnor*, *C. sicula*, *C. yar*.

##### Distribution.

Grenada, Puerto Rico.

##### Biology.

Unknown.

##### Remarks.

Wood’s (2007) measurements of three paratypes differ compared to the measurements of Bright and Torres’ original description (2006) and those of Bright (2019). Given the previously noted observations of calibration error in Wood’s measurements, these values are not included here. Bright (2019) reports that this species has only one suture on the posterior face of the club but it has two.

#### Coptoborus bettysmithae sp. nov.

Taxon classificationAnimaliaColeopteraCurculionidae

FA0951A1-5021-513F-9DE2-E51A2D2C235A

http://zoobank.org/922F74FF-DFC3-4BEB-BA4D-8E5259FAC65D

Figure 2G–I, O

##### Type material.

***Holotype*,** female, Ecuador: Napo Prov. [= Orellana Prov.], Tiputini Biodiversity Station, 00°37'55"S, 076°08'39"W, 220–250 m, June 1998, T.L. Erwin et al. collectors, insecticidal fogging, terra firme forest, trans[ect] 4, sta[tion] 5, Erwin lot #1834 (ICB).

##### Diagnosis.

2.4 mm (n = 1), 2.67 × as long as wide. This species is distinguished by the elytral apex attenuate and weakly emarginate, declivital interstriae 2 convex, declivital interstriae 1–3 denticulate, posterolateral margin of declivity with interstriae 3 and 9 joining, forming a carina and continuing submarginally to apex, stout form, declivital interstriae 1 with a uniseriate row of erect scale-like setae, and posterior ~40% of elytra gradually tapered to apex.

##### Similar species.

*C. barbicauda*, *C. capillisoror*, *C. hansen*, *C. schulzi*, *C. subtilis*, *C. trinity*, *C. uhura*.

##### Description

**(female). *Holotype*** 2.4 mm, 2.67 × as long as wide. Body light brown, elytral declivity darker, antennae and legs lighter. ***Head***: epistoma smooth. Frons dull, finely punctate, setose; each puncture bearing a long, erect hair-like seta. Eyes broadly and moderately emarginate. Submentum narrow, triangular, deeply impressed. Antennal scape short and thick, as long as club. Pedicel shorter than funicle. Club circular, flat, type 3; segment 1 corneous, transverse on anterior face, occupying basal ~1/4; segment 2 broad, transverse, corneous; segments 1 and 2 present on posterior face. ***Pronotum***: 1.1 × as long as wide. In dorsal view long and rounded frontally, type 7, sides parallel in basal 3/4, rounded anteriorly; anterior margin with two serrations. In lateral view tall, type 2, disc flat, summit evident, on basal 1/3. Anterior slope with densely spaced, broad coarse asperities, becoming lower and more strongly transverse towards summit. Disc strongly shiny with dense, minute punctures, some longer hair-like setae at margins. Lateral margins carinate on basal third. ***Elytra***: 1.6 × as long as wide, 1.4 × as long as pronotum. Scutellum minute. Elytra attenuate, parallel-sided in basal 64%, then acutely tapered to apex, apex weakly emarginate. Disc smooth, shiny; strial punctures large, deep, each bearing a recumbent seta the length of a puncture; interstriae flat, minutely, moderately punctate, unarmed, each puncture bearing a long, erect bristle-like seta. Declivity gradually rounded, occupying ~2/5 of elytra, shagreened, shiny, declivital face weakly flattened; striae 1 and 2 feebly impressed, strial punctures larger, deeper than those of disc, each puncture bearing a recumbent seta as long as two punctures; interstriae flat, uniformly denticulate along their entire lengths, denticles spaced by two widths of a denticle, setae semi-erect, scale-like, as long as interstriae 1 width; interstriae 1 with a one row of short setae as described for striae on each side of median erect setae. Posterolateral margin with interstriae 3 and 9 joining, forming a carina and continuing submarginally to apex. ***Legs***: protibiae distinctly triangular, broadest at apical 1/5; apical 1/2 of outer margin with six large, socketed denticles, their length longer than basal width. Meso- and metatibiae flattened; outer margin evenly rounded with six and eight large, socketed denticles, respectively.

##### Etymology.

For Catherine (Betty) Smith, beloved grandmother of SMS. Betty was a remarkable woman who embodied the theme of ‘kick-ass’ women: she was a “Rosie the Riveter” (https://www.loc.gov/rr/program/journey/rosie.html) in her youth and later displayed extraordinary fortitude in her battles with cancer. Noun in genitive.

##### Distribution.

Ecuador (Orellana).

##### Biology.

The holotype was collected by canopy fogging.

#### Coptoborus brevicauda sp. nov.

Taxon classificationAnimaliaColeopteraCurculionidae

05CE47BE-A3A5-5FB4-8064-4550C0A924F0

http://zoobank.org/02BD30EC-C344-4389-9735-51EB185D0B37

Figure 2J–L, P

##### Type material.

***Holotype*,** female, Ecuador: Napo Prov. [= Orellana Prov.], Res[erva]. Ethnica Waorani, 1 km S. Okone Gare Camp, Trans[ect]. Ent[omology]., 00°39'10"S, 076°26'W, 220 m, October 1995, T.L. Erwin et al. collectors, insecticidal fogging, terra firme forest, trans[ect] 6, sta[tion] 5, Erwin lot #1181 (ICB). ***Paratypes***, female, as holotype except: January 1995, trans[ect] 6, sta[tion] 10, Erwin lot #1039 (MSUC, 1); as holotype except: October 1996, trans[ect] 6, sta[tion] 5, Erwin lot #1715 (NMNH, 1).

##### Diagnosis.

2.4–2.6 mm (mean = 2.5 mm; n = 3), 2.4–2.5 × as long as wide. This species is distinguished by the elytral apex attenuate, entire and produced, apical projection the width of striae 2, declivital interstriae 2 convex, declivital interstriae 1–3 denticulate, denticles minute, faint, their height less than 0.5 × interstriae width, posterolateral margin of declivity with interstriae 3 and 9 joining, forming a carina and continuing submarginally to apex, and stout form.

##### Similar species.

*C. gentilis*.

##### Description

**(female).** 2.4–2.6 mm (mean = 2.5 mm; n = 3), 2.4–2.5 × as long as wide (***holotype*** 2.4 mm, 2.4 × as long as wide). Body light brown to brown, antennae and legs lighter. ***Head***: epistoma tuberculate. Frons strongly shiny, finely punctate, setose; each puncture bearing a long, erect hair-like seta. Eyes broadly and moderately emarginate. Submentum large, triangular, deeply impressed. Antennal scape regularly thick, shorter than club. Pedicel shorter than funicle. Club circular, flat, type 3; segment 1 corneous, subconvex on anterior face, occupying basal ~1/4; segment 2 narrow, subconvex, corneous; segments 1 and 2 present on posterior face. ***Pronotum***: 1.0 × as long as wide. In dorsal view basic and parallel-sided, type 2, sides parallel in basal 3/4, rounded anteriorly; anterior margin with four subequal serrations. In lateral view tall, type 2, disc flat, summit pronounced, on basal 2/5. Anterior slope with densely spaced, broad very coarse asperities, becoming lower and more strongly transverse towards summit. Disc dull with sparse, minute punctures, some longer hair-like setae at margins. Lateral margins obliquely costate. ***Elytra***: 1.4–1.5 × as long as wide, 1.4 × as long as pronotum. Scutellum small. Elytra attenuate, parallel-sided in basal 68%, then acutely tapered to apex, apex apically produced, entire. Disc shagreened, dull; strial punctures large, deep, each bearing a recumbent seta the length of a puncture; interstriae flat, minutely, moderately punctate, unarmed, each puncture bearing a long semi-erect bristle-like seta. Declivity gradually rounded, occupying ~1/2 of elytra, shagreened, shiny, declivital face convex; striae not impressed, strial punctures larger, deeper than those of disc, each puncture bearing a semi-erect seta as long as two punctures; interstriae flat, interstriae 1–3 uniformly minutely granulate along their entire lengths, granules faint, their height less than 0.5 × interstriae width, setae semi-erect, bristle-like, in two rows on interstriae 1 and uniseriate on interstriae 2 and 3. Posterolateral margin with interstriae 3 and 9 joining, forming an acute carina and continuing submarginally to apex. ***Legs***: protibiae obliquely triangular, broadest at apical 1/4; apical 1/2 of outer margin with eight large, socketed denticles, their length longer than basal width. Meso- and metatibiae flattened; outer margin evenly rounded with ten and nine large, socketed denticles, respectively.

##### Etymology.

L. *brevis* = short, *cauda* = tail. Noun in apposition.

##### Distribution.

Ecuador (Orellana).

##### Biology.

Specimens were collected by canopy fogging.

#### Coptoborus brigman sp. nov.

Taxon classificationAnimaliaColeopteraCurculionidae

605D4F62-5655-5B6E-AE4A-348D305B45C2

http://zoobank.org/F152D5CE-03CB-48C7-BF08-D0F581F3BDCA

Figure 3A–C, M

##### Type material.

***Holotype*,** female, Ecuador: Napo Prov. [= Orellana Prov.], Res[erva]. Ethnica Waorani, 1 km S. Okone Gare Camp, Trans[ect]. Ent[omology]., 00°39'10"S, 076°26'W, 220 m, October 1996, T.L. Erwin et al. collectors, insecticidal fogging, terra firme forest, trans[ect] 9, sta[tion] 7, Erwin lot #1747 (ICB). ***Paratype***, female, as holotype except: January 1996, trans[ect] 3, sta[tion] 6, Erwin lot #1426 (ICB, 1).

##### Diagnosis.

1.7–1.8 mm (n = 2), 2.57–2.62 × as long as wide. This species is distinguished by the elytral apex broadly rounded and entire, posterolateral margin continuously and smoothly carinate to striae 6 and not extended posteriad, declivital interstrial setae stout and scale-like, declivital interstriae minutely granulate, and declivital striae not impressed.

##### Similar species.

*C. leia*, *C. tristiculus*, *Euwallacea perbrevis*.

##### Description

**(female).** 1.7–1.8 mm (n = 2), 2.57–2.62 × as long as wide (***holotype*** 1.8 mm, 2.57 × as long as wide). Body light brown, elytral declivity darker, antennae and legs lighter. ***Head***: epistoma smooth. Frons strongly shiny, finely punctate, setose; each puncture bearing a long, erect hair-like seta. Eyes broadly and moderately emarginate. Submentum narrow, triangular, deeply impressed. Antennal scape regularly thick, much shorter than club. Pedicel shorter than funicle. Club circular, flat, type 3; segment 1 corneous, subconvex on anterior face, occupying basal ~1/3; segment 2 narrow, corneous; segments 1 and 2 present on posterior face. ***Pronotum***: 1.1–1.2 × as long as wide. In dorsal view long and rounded frontally, type 7, sides parallel in basal 3/4, rounded anteriorly; anterior margin with four projecting serrations, median pair larger than lateral pair. In lateral view elongate, disc anterior slope subequal, type 7, summit prominent, at midpoint. Anterior slope with densely spaced, broad coarse asperities, becoming lower and more strongly transverse towards summit. Disc strongly shiny with moderately dense, minute punctures, some longer hair-like setae at margins. Lateral margins obliquely costate. ***Elytra***: 1.4 × as long as wide, 1.25 × as long as pronotum. Scutellum minute. Elytra rounded, parallel-sided in basal 80%, then broadly rounded to apex, apex entire. Disc shagreened, dull; strial punctures large, deep, each bearing a recumbent seta the length of a puncture; interstriae flat, minutely, moderately punctate, unarmed, each puncture bearing a long semi-erect bristle-like seta. Declivity gradually rounded, occupying ~2/5 of elytra, smooth, shiny, declivital face flattened; striae not impressed, strial punctures larger, deeper than those of disc, each puncture bearing a semi-erect seta as long as two punctures; interstriae flat, uniformly minutely granulate along their entire lengths, setae stout, semi-erect, scale-like. Posterolateral margin continuously and smoothly carinate to striae 6. ***Legs***: protibiae obliquely triangular, broadest at apical 1/4; apical 1/2 of outer margin with six or seven large, socketed denticles, their length longer than basal width. Meso- and metatibiae flattened; outer margin evenly rounded with eight and nine large, socketed denticles, respectively.

##### Etymology.

Portrayed by Mary Elizabeth Mastrantonio, Dr. Lindsey Brigman is the heroine in the movie ‘The Abyss’ (1989). Noun in apposition.

##### Distribution.

Ecuador (Orellana).

##### Biology.

The type specimens were collected by canopy fogging.

**Figure 3.**
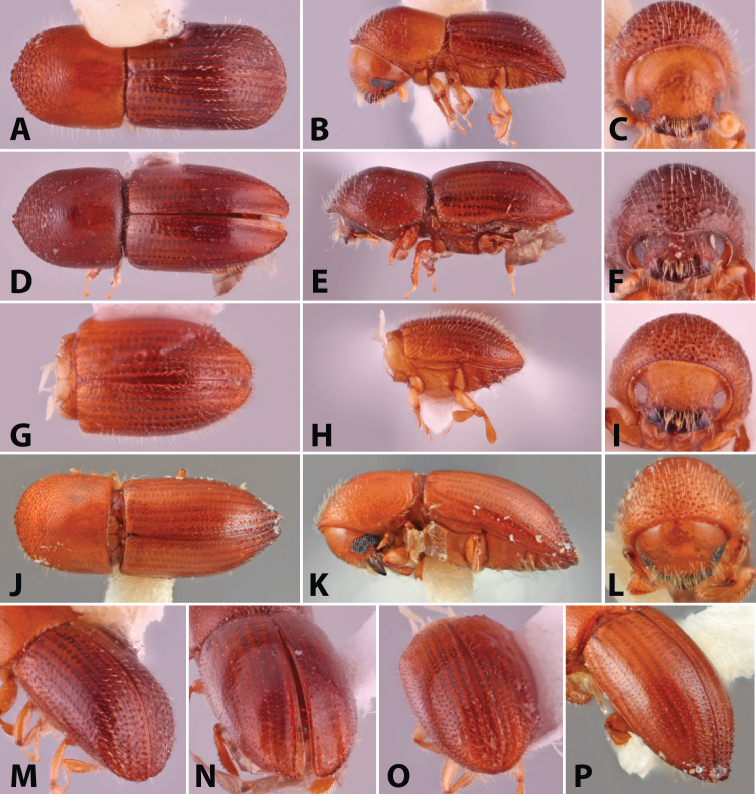
Dorsal, lateral, frontal and declivital view of *Coptoborus brigman* holotype, 1.8 mm (**A–C, M**), *C. busoror* holotype, 2.7 mm (**D–F, N**), *C. capillisoror* holotype, 2.3 mm (**G–I, O**), *C. carumbensis* holotype, 2.2 mm (**J–L, P**). All photographs by SMS, **J–L, P** copyright National Museum of Natural History, Smithsonian Institution, Washington, D.C., published by permission.

#### Coptoborus busoror sp. nov.

Taxon classificationAnimaliaColeopteraCurculionidae

4C9F5EF0-AF72-5382-A0DF-BC0A710E97C5

http://zoobank.org/84C444F8-1056-4C6E-9375-3399AE400B8D

Figure 3D–F, N

##### Type material.

***Holotype*,** female, Ecuador: Napo [= Orellana], Via Kerrmegee, Sta. Rosa, 1°5'77"S, 17[sic, possibly 77]°34'14"W, 377 m, 21 Sep 2000, M. Vallejo R., ex. *Astrocaryum urostachys* (PUCE).

##### Diagnosis.

2.7 mm (n = 1), 2.7 × as long as wide. This species is distinguished by the elytral apex attenuate and weakly emarginate, declivity distinctly sulcate along interstriae 2, declivital interstriae 2 unarmed, interstriae 1 and 3 armed, declivital striae 1 and 2 parallel on declivital face and widely spaced, and declivital striae 2 punctate. It is most similar to *C. ochromactonus* and can be further distinguished by the larger size 2.7 mm vs. 2.5–2.6 mm, and more elongate body 2.7 × as long as wide vs. 2.5–2.6 × as long as wide, more elongate pronotum 1.2 × as long as wide vs. 1.05–1.1 × as long as wide, and distribution east of the Andes vs. west of the Andes.

##### Similar species.

*C. leeloo*, *C. nudulus*, *C. ochromactonus*, *C. pilisoror*, *C. ripley*, *C. sororcula*, *C. spicatus*.

##### Description

**(female). *Holotype*** 2.7 mm, 2.7 × as long as wide. Body brown, antennae and legs lighter. ***Head***: epistoma smooth. Frons strongly shiny, finely punctate, setose; each puncture bearing a long, erect hair-like seta. Eyes broadly and moderately emarginate. Submentum narrow, triangular, deeply impressed. Antennal scape regularly thick, shorter than club. Pedicel shorter than funicle. Club circular, flat, type 3; segment 1 corneous, subconvex on anterior face, occupying basal ~1/4; segment 2 narrow, subconvex, corneous; segments 1 and 2 present on posterior face. ***Pronotum***: 1.2 × as long as wide. In dorsal view long and rounded frontally, type 7, sides parallel in basal 3/4, rounded anteriorly; anterior margin with two projecting serrations. In lateral view elongate, disc longer than anterior slope, type 7, summit prominent, on anterior 3/4. Anterior slope with densely spaced, broad coarse asperities, becoming lower and more strongly transverse towards summit. Disc reticulate, subshiny with moderately dense, minute punctures, some longer hair-like setae at margins. Lateral margins carinate on basal third. ***Elytra***: 1.5 × as long as wide, 1.25 × as long as pronotum. Scutellum minute. Elytra attenuate, parallel-sided in basal 2/3, then acutely rounded to apex, apex weakly emarginate. Disc smooth, dull; strial punctures moderate, shallow, glabrous; interstriae flat, sparsely, minutely punctate, unarmed, each puncture bearing a long semi-erect hair-like seta. Declivity gradual, smooth, shiny, appearing bisulcate, occupying apical 2/5 of elytra; striae not impressed, striae 1 and 2 parallel, strial punctures much larger and shallower than those of disc; interstriae 2 weakly sulcate, unarmed, punctate; interstriae 1 and 3 weakly costate with six and five minute granules, each granule bearing a long semi-erect hair-like seta. Posterolateral margin with interstriae 3 and 9 joining, forming a weakly serrate acute carina and continuing submarginally to apex. ***Legs***: protibiae obliquely triangular, broadest at apical 1/4; apical 1/2 of outer margin with seven large, socketed denticles, their length longer than basal width. Meso- and metatibiae flattened; outer margin evenly rounded with seven large, socketed denticles.

##### Etymology.

L. *bu* = big, *soror* = sister. Noun in apposition.

##### Distribution.

Ecuador (Orellana).

##### Biology.

This species has been recorded from *Astrocaryum urostachys* (Arecaceae).

#### Coptoborus capillisoror sp. nov.

Taxon classificationAnimaliaColeopteraCurculionidae

9C9A16AA-7A28-5353-92B8-267EBF420302

http://zoobank.org/ACF70BDC-D17D-48D7-AFA9-19B0B50B544C

Figure 3G–I, O

##### Type material.

***Holotype*,** female, Brazil: Bahia, Camacan, Serra Bonita Reserve, 15°23.429'S, 39°33.810'W, 700–100 m, 6–14.V.2013, AI Cognato, SM Smith, CAH Flechtmann, #115, ex *Tibouchina*, DNA voucher Theo.sp1 (MZUSP).

##### Diagnosis.

2.3 mm (n = 1), 2.88 × as long as wide. This species is distinguished by the elytral apex attenuate and entire and not produced, declivity interstriae 2 feebly sulcate, declivital interstriae 1– 3 denticulate, denticles on interstriae 1 and 3 small and relatively indistinct, interstriae 1 unarmed on apical quarter, posterolateral margin of declivity with interstriae 3 and 9 joining, forming a carina and continuing submarginally to apex, stout form, and declivital interstriae and striae densely covered with abundant hair-like setae, setae becoming thicker from base to apex.

##### Similar species.

*C. barbicauda*, *C. bettysmithae*, *C. hansen*, *C. schulzi*, *C. subtilis*, *C. trinity*, *C. uhura*.

##### Description

**(female). *Holotype*** 2.3 mm, 2.88 × as long as wide. Body light brown, antennae and legs lighter. ***Head***: epistoma smooth. Frons dull, finely punctate, setose; each puncture bearing a long, erect hair-like seta. Eyes narrowly and deeply emarginate. Submentum narrow, triangular, slightly impressed. Antennal scape regularly thick, shorter than club. Pedicel shorter than funicle. Club circular, flat, type 3; segment 1 corneous, weakly bisinuate on anterior face, occupying basal ~1/4; segment 2 narrow, weakly bisinuate, corneous; segments 1 and 2 present on posterior face. ***Pronotum***: 1.0 × as long as wide. In dorsal view basic and parallel-sided, type 2, sides parallel in basal 3/4, rounded anteriorly; anterior margin with four projecting serrations, median pair larger. In lateral view tall, type 2, disc flat, summit pronounced, at midpoint. Anterior slope with densely spaced, broad fine asperities, becoming lower and more strongly transverse towards summit. Disc reticulate, subshiny with moderately dense, moderate punctures, some longer hair-like setae at margins. Lateral margins obliquely costate. ***Elytra***: 1.9 × as long as wide, 1.9 × as long as pronotum. Scutellum minute. Elytra attenuate, parallel-sided in basal 2/3, then acutely rounded to apex, apex entire. Disc smooth, shiny; strial punctures large, deep, each bearing a recumbent seta the length of a puncture; interstriae flat, sparsely, minutely punctate, unarmed, each puncture bearing a long fine semi-erect hair-like seta. Declivity gradually rounded, occupying ~1/2 of elytra, shagreened, subshiny, declivital face feebly sulcate; striae distinctly impressed, strial punctures larger, deeper than those of disc, each puncture bearing a semi-recumbent hair-like seta as long as 3–5 punctures; interstriae flat, sparsely and inconsistently denticulate, denticles uniseriate, spaced by at least six widths of a denticle, denticles absent on apical 1/4 of interstriae 1, interstriae 3 denticles faint, setae dense, uniseriate, long, erect, hair-like at the base and gradually increasing in thickness toward apex, 1.5 × as long as interstriae 1 width, interstriae 1 with an additional row of slightly shorter setae. Posterolateral margin with interstriae 3 and 9 joining, forming a feeble carina and continuing submarginally to apex ***Legs***: protibiae obliquely triangular, broadest at apical 1/3; apical 1/2 of outer margin with eight large, socketed denticles, their length longer than basal width. Meso- and metatibiae flattened; outer margin evenly rounded with nine and seven large, socketed denticles, respectively.

##### Etymology.

L. *capillosus* = hairy *soror* = sister. In reference to the abundant long setae of the declivity. Noun in apposition.

##### Distribution.

Brazil (Bahia).

##### Biology.

This species was collected from *Tibouchina* (Melastomataceae).

#### Coptoborus carumbensis

Taxon classificationAnimaliaColeopteraCurculionidae

Wood, 2007

171B027C-E65B-59F2-B378-3AF9289BC936

Figure 3J–L, P

Coptoborus carumbensis Wood, 2007: 399.

##### Type material.

***Holotype*** (NMNH), examined.

##### New records.

None.

##### Diagnosis.

2.2 mm, 2.5 × as long as wide. This species is distinguished by the elytral apex attenuate and strongly emarginate, declivity convex, declivital interstriae 2 denticulate, elytral apex with interstriae 3 and 9 joining, forming a crenulate carina that continues submarginally to apex, crenulation next to suture larger than other crenulations, declivital interstriae 3 densely denticulate with fewer than ten denticles, and declivital striae 1–3 impressed.

##### Similar species.

*C. asperatus*.

##### Distribution.

Brazil (Espírito Santo), Paraguay (San Pedro).

##### Biology.

Unknown.

#### Coptoborus catulus

Taxon classificationAnimaliaColeopteraCurculionidae

(Blandford, 1898)

521B4146-F6BD-54AA-8D04-F16F25DFFA27

Figure 4A–C, M

Xyleborus catulus Blandford, 1898: 215.
Coptoborus catulus (Blandford): Wood and Bright 1992: 663.
Xyleborus intricatus Schedl, 1948: 274. Synonymy: Wood 1975a: 23.

##### Type material.

***Holotype****Xyleborus catulus* (NHMUK), not examined. ***Holotype****Xyleborus intricatus* Schedl (NHMW), examined.

##### New records.

Brazil: Paraná, Rondon, 23.I.[19]53, F. Plaumann (NHMW, 1). Ecuador: Los Ríos, Canton La Clementina, Samama Nature Reserve, 01°38.852'S, 79°19.867'W, 381–430 m, 13–15.v.2015, Cognato, Smith, Osborn, Martinez et al., sample EC 30, ex buttressed tree, 30 cm DBH (MSUC, 2; PUCE, 1). Panama: Panamá Prov., [Parque Nacional Soberanía], Pipeline Rd, 9°9.222'N, 79°44.25'W, 65 m, 3.ix.2008, S.M. Smith, A.D. Smith, A.R. Gillogly, PAN 116, [ex. Malvaceae] (MSUC, 3).

##### Diagnosis.

1.8–2.2 mm (mean = 2.1 mm; n = 5), 3.0–3.14 × as long as wide. This species is distinguished by the elytral apex attenuate and weakly emarginate, declivital interstriae 1–3 denticulate, interstriae 2 with fewer denticles than interstriae 1 or 3, and posterolateral margin of declivity costate, armed with two denticles.

##### Similar species.

*C. amplissimus*, *C. incomptus*, *C. newt*, *C. scully*.

##### Distribution.

Brazil (Paraná*, Santa Catarina), Ecuador* (Los Ríos), Mexico (Oaxaca, Tabasco, Veracruz), Panama (Colón, Panamá*), Peru (Madre de Dios), Suriname, Venezuela (Barinas, Mérida).

##### Biology.

This species has only been recorded from *Guazuma ulmifolia* (Malvaceae) (Wood and Bright 1992) and an unidentified Malvaceae. Wood (1982) reported collecting specimens from new tunnels in recently cut limbs and boles 5–20 cm in diameter.

##### Remarks.

The holotype of *X. catulus* was not directly examined by the authors. Our concept of the species is based Blandford’s (1898) description, and a specimen compared to the holotype by S.L. Wood in 1972 with the following locality: Panama, 24.V.[19]49, wood with orchids, mobile 7756 49 7569 (NMNH).

**Figure 4.**
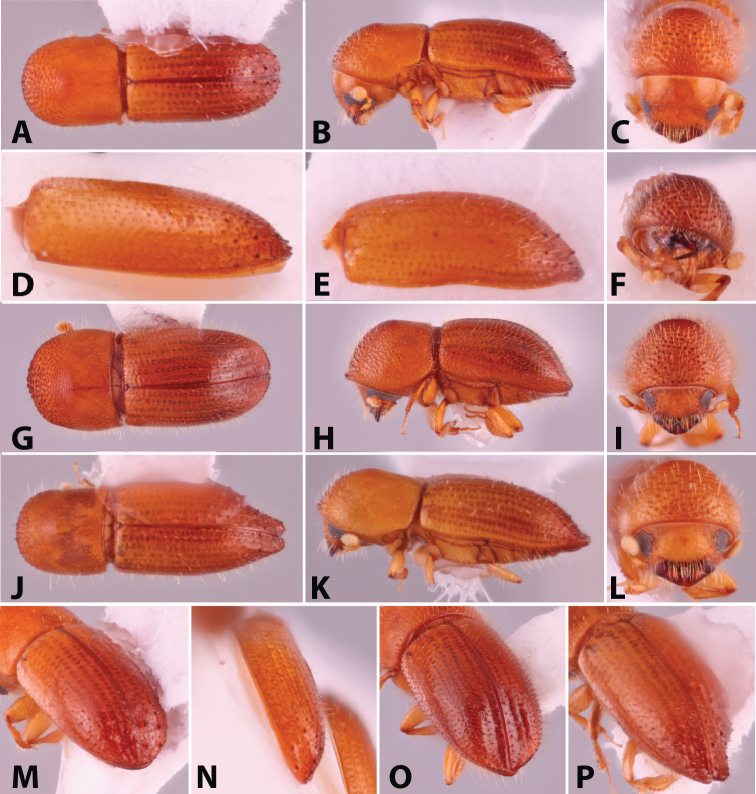
Dorsal, lateral, frontal and declivital view of *Coptoborus catulus* 2.2 mm (**A–C, M**), *C. chica* holotype, 2.0 mm (**D–F, N**), *C. coartatus* 2.7–2.9 mm (**G–I, O**), *C. cracens* 1.8–2.0 mm (**J–L, P**). All photographs by SMS.

#### Coptoborus chica sp. nov.

Taxon classificationAnimaliaColeopteraCurculionidae

3FF830AF-2618-50F0-9B98-0D20EF41E845

http://zoobank.org/B0B2EED2-87F6-4378-9A6D-574CEEECA656

Figure 4D–F, N

##### Type material.

***Holotype*,** female, Suriname: Sipaliwini, 2.977312°N, 55.38500°W, 200 m, Camp 4 (low), Kasikasima, T. Larsen, 20–25.iii.2012, FIT, SR12-0320-TN1, 2012 CI-RAP survey (NZCS).

##### Diagnosis.

2.0 mm (n = 1), 3.33 × as long as wide. This species is distinguished by the elytral apex attenuate and entire, declivity interstriae 2 convex, declivital interstriae 1 and 3 denticulate and interstriae 2 unarmed, declivital subapical margin armed with three denticles, and declivital interstriae 1 denticles small, 0.5–1 × high as wide.

##### Similar species.

*C. papillicauda*.

##### Description

**(female). *Holotype*** 2.0 mm, 3.33 × as long as wide. Body, antennae, and legs light brown. ***Head***: epistoma tuberculate. Frons strongly shiny, finely punctate, setose; each puncture bearing a long, erect hair-like seta. Eyes narrowly and deeply emarginate. Submentum narrow, triangular, slightly impressed. Antennal scape regularly thick, shorter than club. Pedicel shorter than funicle. Club circular, flat, type 3; segment 1 corneous, transverse on anterior face, occupying basal ~1/5; segment 2 narrow, transverse, corneous; segments 1 and 2 present on posterior face. ***Pronotum***: 1.3 × as long as wide. In dorsal view long and rounded frontally, type 7, sides parallel in basal 4/5, rounded anteriorly; anterior margin without serrations. In lateral view elongate, disc longer than anterior slope, type 7, summit prominent, on anterior 3/4. Anterior slope with densely spaced, broad fine asperities, becoming lower and more strongly transverse towards summit. Disc dull with sparse, minute punctures, some longer hair-like setae at margins. Lateral margins obliquely costate. ***Elytra***: 2.0 × as long as wide, 1.5 × as long as pronotum. Scutellum small. Elytra attenuate, parallel-sided in basal 3/4, then acutely rounded to apex, apex entire. Disc shiny; striae minutely punctate, glabrous; interstriae flat, sparsely, minutely punctate, unarmed, glabrous. Declivity gradually rounded, occupying ~1/3 of elytra, smooth, shiny, declivital face weakly convex; striae very shallowly impressed, strial punctures larger, deeper than those of disc, glabrous, striae 1 irregular, slightly laterally broadened from base to declivital midpoint and then narrowing towards apex; interstriae flat, interstriae 1 and 3 each with three small denticles, interstriae 2 unarmed, those of interstriae 1 and 3 subequal, 0.5–1 × high as wide, interstriae with a sparse row of erect bristle-like setae. Posterolateral margin with interstriae 3 and 9 joining, forming a feebly carina armed with three small denticles and continuing submarginally to apex. ***Legs***: protibiae obliquely triangular, broadest at apical 1/3; apical 1/2 of outer margin with seven large, socketed denticles, their length longer than basal width. Meso- and metatibiae flattened; outer margin evenly rounded with nine and seven large, socketed denticles, respectively.

##### Etymology.

Spanish, *chica* = girl. Noun in apposition.

##### Distribution.

Suriname.

##### Biology.

Unknown.

#### Coptoborus coartatus

Taxon classificationAnimaliaColeopteraCurculionidae

(Sampson, 1921) comb. nov.

DFC5439B-807A-5FF4-86AA-C6A4250BC64E

Figure 4G–I, O

Xyleborus coartatus Sampson, 1921: 32.
Theoborus coartatus (Sampson): Wood 1982: 776.
Xyleborus artecuneolus Schedl, 1939: 14: Synonymy: Wood 1966: 31.

##### Type material.

***Holotype****Xyleborus coartatus* (NHMUK), examined.

##### New records.

Ecuador: Los Ríos Prov., Canton La Clementina, Samama Nature Reserve, 01°38.852'S, 79°19.867'W, 381–430 m, 13–15.v.2015, Cognato, Smith, Osborn, Martinez et al., sample EC 13, ex 4 cm diameter hanging liana (MSUC, 4; PUCE, 1); Napo Prov. [= Orellana Prov.], Tiputini Biodiversity Station, 00°37'55"S, 076°08'39"W, 220–250 m, June 1998, T.L. Erwin et al. collectors, insecticidal fogging, terra firme forest, trans[ect] 2, sta[tion] 1, Erwin lot #1810 (ICB, 1). Peru: Madre de Dios Dept., Los Amigos Biological Station, 12°34.9'S, 70°6.04'W, Smith, Hulcr, 26.iv.–2.v.2008, sample Peru 40b, 4 cm twig (MSUC, 3); as previous except: CM2, GPS 12.4492°S, 70.2517°W, Smith, Hulcr, 17–18.v.2008, sample Peru 88c 3 cm diameter branch (MSUC, 7).

##### Diagnosis.

2.7–2.9 mm (mean = 2.82 mm; n = 5), 2.33–2.55 × as long as wide. This species is distinguished by the elytral apex broadly rounded and entire, posterolateral margin continuously and smoothly carinate to interstriae 6, extended posteriad and appearing shelf-like, and declivity moderately impressed along interstriae 2.

##### Similar species.

*C. ricini*.

##### Distribution.

Brazil (Bahia, Minas Gerais), Colombia (Cundinamarca, Tolima, Valle de Cauca), Costa Rica (Limón, San José), Ecuador* (Los Ríos, Orellana), Mexico (Chiapas), Panama (Chiriquí), Peru (Madre de Dios), Trinidad.

##### Biology.

This species is known from *Mimosa* (Fabaceae), *Theobroma cacao*, and an unidentified *Theobroma* (Malvaceae) (Wood and Bright 1992). Wood (1982) reported collecting the species from boles of small trees 8–20 cm in diameter but has been collected in smaller 3–4 cm diameter branches in Ecuador and Peru. It is considered a minor pest of cacao (Wood 2007).

##### Remarks.

Wood (2007) reports this species as introduced into Africa but published reports were not located. This erroneous record appears to be due to a reference to the species in the description of the African species *Xyleborus ovatus*Eggers (1932: 298) in which Eggers compared the form of *X. ovatus* to that of the South American species *X. coartatus* but did not state that the latter species occurred in Africa.

#### Coptoborus cracens

Taxon classificationAnimaliaColeopteraCurculionidae

Wood, 2007

530EFAAB-85F4-560A-BDD7-F16F9002456E

Figure 4J–L, P

Coptoborus cracens Wood, 2007: 400.

##### Type material.

***Holotype*** (MEFEIS), not examined. ***Paratype*** (NMNH), examined.

##### New records.

Ecuador: Napo Prov. [= Orellana Prov.], Res[erva]. Ethnica Waorani, 1 km S. Okone Gare Camp, Trans[ect]. Ent[omology]., 00°39'10"S, 076°26'W, 220 m, January 1994, T.L. Erwin et al. collectors, insecticidal fogging, terra firme forest, trans[ect] 7, sta[tion] 2, Erwin lot #611 (ICB, 1); as previous except: July 1994, trans[ect] 6, sta[tion] 1, Erwin lot #750 (NMNH, 1); as previous except: July 1995, trans[ect] 4, sta[tion] 4, Erwin lot #1094 (ICB, 1); as previous except: trans[ect] 9, sta[tion] 3, Erwin lot #1113 (MSUC, 1); as previous except: October 1995, trans[ect] 2, sta[tion] 1, Erwin lot #1181 (ICB, 1); as previous except: January 1996, trans[ect] 2, sta[tion] 1, Erwin lot #1411 (NMNH, 1); as previous except: July 1996, trans[ect] 2, sta[tion] 1, Erwin lot #1531 (ICB, 1); as previous except: October 1996, trans[ect] 2, sta[tion] 1, Erwin lot #1671 (MSUC, 1; NMNH, 1); Tiputini Biodiversity Station, 00°37'55"S, 076°08'39"W, 220–250 m, October 1995, T.L. Erwin et al. collectors, insecticidal fogging, terra firme forest, trans[ect] 9, sta[tion] 9, Erwin lot #1259 (ICB, 1); as previous except: February 1999, trans[ect] 4, sta[tion] 3, Erwin lot # 2032 (ICB, 1; NMNH, 2); as previous except: trans[ect] 4, sta[tion] 6, Erwin lot #2035 (ICB, 1); as previous except: Yasuni National Park, Estacíon Científica Yasuní, 00°39.675'S, 76°24.023'W, 11.ii.2018, R. Osborn, EC18-41, ex 6 cm dia. branch (MSUC, 6; PUCE, 1). Peru: Madre de Dios Dept., Los Amigos Biological Station, 12°34.9S, 70°6.04W, Smith, Hulcr, 26.iv.–2.v.2008, sample Peru 52, branch (MSUC, 3).

##### Diagnosis.

1.8–2.0 mm (mean = 1.86 mm; n = 5), 3.0–3.33 × as long as wide. This species is distinguished by the elytral apex attenuate and strongly emarginate, declivity convex, declivital interstriae 2 denticulate, elytral apex with interstriae 3 and 9 joining, forming a smooth continuous carina that continues submarginally to apex, apex produced, declivital interstriae 3 denticles much larger than those of interstriae 1, and interstriae setae with short erect bristle-like setae that are shorter than interstrial width.

##### Similar species.

*C. leporinus*, *C. gracilens*.

##### Distribution.

Brazil (Espírito Santo), Ecuador* (Orellana), Peru* (Madre de Dios).

##### Biology.

Specimens were collected by canopy fogging as well from the wood of an unidentified branch.

##### Remarks.

Wood (2007) incorrectly reported the holotype’s location as MZUSP; the holotype is in MEFEIS.

#### Coptoborus crassisororcula sp. nov.

Taxon classificationAnimaliaColeopteraCurculionidae

A6CC0B4A-3BC2-5136-8218-E5DFCC3B2D13

http://zoobank.org/ED5AA5F6-70D6-4A94-A1C5-1B38362CEDD7

Figure 5A–C, M

##### Type material.

***Holotype*,** female, Peru: Madre de Dios Dept., Los Amigos Biological Station, 12°34.9S, 70°6.04W, Smith, Hulcr, 26.iv.–27.v.2008, sample Peru 52 (MUSM). ***Paratypes***, female, as holotype (MSUC, 1; MUSM, 1; NHMUK, 1; NMNH, 1).

##### Diagnosis.

1.8–1.9 mm (mean = 1.86 mm; n = 5), 2.38–2.57 × as long as wide. This species is distinguished by the elytral apex attenuate and weakly emarginate, declivital interstriae 2 granulate only on basal third, declivital interstriae 2 moderately sulcate, posterolateral margin of declivity with interstriae 3 and 9 joining, forming a carina and continuing submarginally to apex, declivital interstriae distinctly impressed, anterior margin of pronotum with a pair of projecting serrations.

##### Similar species.

*C. atlanticus*, *C. incultus*.

##### Description

**(female).** 1.8–1.9 mm (mean = 1.86 mm; n = 5), 2.38–2.57 × as long as wide (***holotype*** 1.8 mm, 2.57 × as long as wide). Body light brown to brown, antennae, and legs lighter. ***Head***: epistoma tuberculate. Frons dull, finely punctate, setose; each puncture bearing a long, erect hair-like seta. Eyes narrowly and deeply emarginate. Submentum narrow, triangular, slightly impressed. Antennal scape short and thick, much shorter than club. Pedicel shorter than funicle. Club circular, flat, type 3; segment 1 corneous, weakly convex on anterior face, occupying basal ~1/4; segment 2 narrow, weakly convex, corneous; segments 1 and 2 present on posterior face. ***Pronotum***: 1.0 × as long as wide. In dorsal view basic and parallel-sided, type 2, sides parallel in basal 3/4, rounded anteriorly; anterior margin with two projecting serrations. In lateral view tall, type 2, disc flat, summit pronounced, at midpoint. Anterior slope with densely spaced, broad coarse asperities, becoming lower and more strongly transverse towards summit. Disc reticulate, dull with sparse, minute punctures, some longer hair-like setae at margins. Lateral margins obliquely costate. ***Elytra***: 1.5–1.6 × as long as wide, 1.6 × as long as pronotum. Scutellum minute. Elytra attenuate, parallel-sided in basal 66–72%, then acutely tapered to apex, apex weakly emarginate. Disc smooth, dull; strial punctures moderate, shallow, glabrous; interstriae flat, sparsely, minutely punctate, unarmed, glabrous. Declivity gradual, shagreened, dull, appearing bisulcate, occupying apical 2/5 of elytra; striae not impressed, striae 1 and 2 parallel, strial punctures much larger and shallower than those of disc, glabrous; interstriae impunctate, interstriae 2 moderately sulcate, minutely granulate on basal third, glabrous; interstriae 1 and 3 moderately costate with eight small granules, covered with three confused rows of minute recumbent setae. Posterolateral margin with interstriae 3 and 9 joining, forming a granulate carina and continuing submarginally to apex. ***Legs***: protibiae semi-circular with evenly rounded outer margin, broadest at apical 1/3; apical 1/2 of outer margin with six large, socketed denticles, their length longer than basal width. Meso- and metatibiae flattened; outer margin evenly rounded with seven and six large, socketed denticles, respectively.

##### Etymology.

L. *crassus* = stout, *soror* = sister, -*culus* = little. Noun in apposition.

##### Distribution.

Peru (Madre de Dios).

##### Biology.

Collected from wood of an unidentified host.

**Figure 5.**
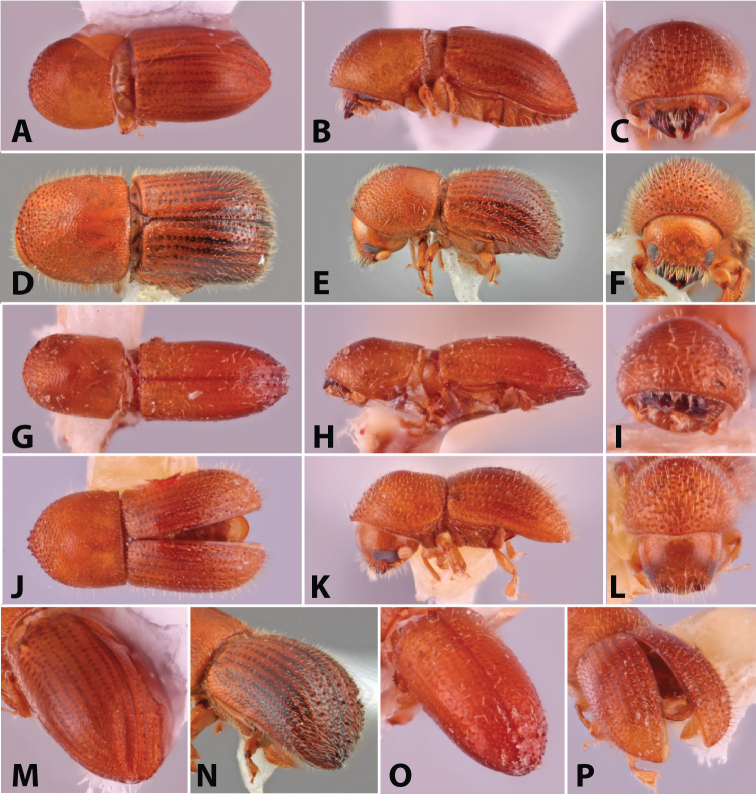
Dorsal, lateral, frontal and declivital view of *Coptoborus crassisororcula* holotype, 1.8–1.9 mm (**A–C, M**), *C. crinitulus* holotype, 1.9–2.5 mm (**D–F, N**), *C. cuneatus* syntype, 2.1 mm (**G–I, O**), *C. doliolum* holotype, 1.7 mm (**J–L, P**). All photographs by SMS, **D–F, N** copyright National Museum of Natural History, Smithsonian Institution, Washington, D.C., published by permission.

#### Coptoborus crinitulus

Taxon classificationAnimaliaColeopteraCurculionidae

(Wood, 1974) comb. nov.

4D8C31F2-573E-5D93-A9AC-4D0ECC3A487E

Figure 5D–F, N

Xyleborus crinitulus Wood, 1974: 34.
Theoborus crinitulus (Wood): Wood 1982: 774.
Xyleborus crinitulus Wood: Wood and Bright 1992: 721.
Theoborus crinitulus (Wood): Wood 2007: 389.

##### Type material.

***Holotype*** (NMNH), ***paratypes*** (NMNH, 2), examined.

##### New records.

None.

##### Diagnosis.

1.9–2.5 mm, 2.3–2.5 × as long as wide (Wood 2007; Bright 2019). This species is distinguished by the broadly rounded and entire elytral apex, posterolateral margins of elytra distinctly carinate to interstriae 8, bearing crenulations of equal size along its length, and very stout elytra, 1.3 × as long as wide.

##### Similar species.

*Ambrosiodmus* Hopkins, 1915 spp.

##### Distribution.

Panama (Panamá), Saint Lucia, Venezuela (Barinas).

##### Biology.

This species is only known from *Hirtella triandra* (Chrysobalanaceae) (Wood 2007).

##### Remarks.

Wood (2007) reports this species as occurring in Africa however we could not locate further details or published records of this claim. The report from Africa should be considered dubious.

#### Coptoborus cuneatus

Taxon classificationAnimaliaColeopteraCurculionidae

(Eichhoff, 1878)

FB800B12-3BA4-5B5F-AB22-39F345F20679

Figure 5G–I, O

Xyleborus cuneatus Eichhoff, 1878: 380.
Coptoborus cuneatus (Eichhoff): Wood and Bright 1992: 663.

##### Type material.

***Syntypes*** (NHMW, 1; examined) and (MIIZ, 1; not examined) (Węgrzynowicz and Mokrzecki 1996). See remarks.

##### Diagnosis.

2.1 mm (n = 1), 3.5 × as long as wide. This species is distinguished by the elytral apex attenuate and weakly emarginate, declivital interstriae 2 convex, declivital interstriae 1–3 unarmed, elytral apex deeply emarginate, and declivity steep, occupying less than posterior 40% of declivity.

##### Similar species.

*C. exutus*, *C. galacatosae*.

##### Distribution.

Panama (Panamá), Peru (Huánuco), Venezuela (Barinas).

##### Biology.

Unknown.

##### Remarks.

Eichhoff (1878) described the species from an unspecified number of specimens. Schedl (1979) apparently presumed that the specimen of *X. cuneatus* in his collection to be a holotype under the assumption that the species was described from a single specimen. This is not the case, as an additional syntype is in MIIZ along with several other Eichhoff types (Węgrzynowicz and Mokrzecki 1996; Cognato et al. 2019; Smith et al. 2019b). Both specimens are therefore syntypes.

The species was described from specimens collected in Varinas [sic, Barinas], Nova Grenada. The species was listed from Colombia (Wood and Bright 1992; Wood 2007) but we were unable to find specimens definitively from this location (Wood 2007). The type specimens were likely collected from Barinas, Venezuela.

#### Coptoborus doliolum sp. nov.

Taxon classificationAnimaliaColeopteraCurculionidae

1DF14C05-D320-5EAB-A195-114167E1636F

http://zoobank.org/F85E24B9-5557-4FC4-AE89-302B124A2A3F

Figure 5J–L, P

##### Type material.

***Holotype*,** female, Ecuador: Napo Prov. [= Orellana Prov.], Tiputini Biodiversity Station, 00°37'55"S, 076°08'39"W, 220–250 m, February 1999, T.L. Erwin et al. collectors, insecticidal fogging, terra firme forest, trans[ect] 4, sta[tion] 8, Erwin lot #2037 (ICB).

##### Diagnosis.

1.7 mm (n = 1), 2.43 × as long as wide. This species is distinguished by the broadly rounded and entire elytral apex, posterolateral margin rounded, anterior margin of pronotum with two projecting serrations, declivital interstrial setae much longer than the combined width of striae 1 and interstriae 1, declivital interstriae flat and all interstriae uniformly armed by denticles along their length.

##### Similar species.

*C. erwini*, *C. paurus*.

##### Description

**(female). *Holotype*** 1.7 mm, 2.43 × as long as wide. Body light brown, antennae and legs lighter. ***Head***: epistoma smooth. Frons dull, finely punctate, setose; each puncture bearing a long, erect hair-like seta. Eyes broadly and moderately emarginate. Submentum narrow, triangular, slightly impressed. Antennal scape short and thick, much shorter than club. Pedicel shorter than funicle. Club longer than wide, flat, type 3; segment 1 corneous, transverse on anterior face, occupying basal ~1/3; segment 2 narrow, transverse, corneous; segments 1 and 2 present on posterior face. ***Pronotum***: 1.0 × as long as wide. In dorsal view basic and parallel-sided, type 2, sides parallel in basal 3/4, rounded anteriorly; anterior margin with two projecting serrations. In lateral view tall, type 2, disc flat, summit pronounced, at midpoint. Anterior slope with densely spaced, broad coarse asperities, becoming lower and more strongly transverse towards summit. Disc dull, with moderately dense, minute punctures, some longer hair-like setae at margins. Lateral margins obliquely costate. ***Elytra***: 1.6 × as long as wide, 1.6 × as long as pronotum. Scutellum small. Elytra rounded, parallel-sided in basal 82%, then broadly rounded to apex, apex entire. Disc smooth, subshiny; strial punctures large, deep, each bearing a recumbent seta the length of a puncture; interstriae flat, minutely, moderately punctate, unarmed, each puncture bearing a long semi-erect bristle-like seta. Declivity gradually rounded, occupying ~2/3 of elytra, smooth, shiny, declivital face convex; striae weakly impressed, strial punctures much larger, deeper than those of disc, each puncture bearing a semi-erect seta half as long as those of interstriae; interstriae flat, uniformly granulate along their entire lengths, granules small, interstriae 1–7 each with a row of long, erect setae much longer than the combined width of striae 1 and interstriae 1; interstriae 1 with a one row of short setae as described for striae on each side of median erect setae. Posterolateral margin rounded. ***Legs***: protibiae semi-circular with evenly rounded outer margin, broadest at apical 1/3; apical 1/2 of outer margin with five large, socketed denticles, their length longer than basal width. Meso- and metatibiae flattened; outer margin evenly rounded with seven large, socketed denticles.

##### Etymology.

L. *doliolum* = little wine cask. Noun in apposition.

##### Distribution.

Ecuador (Orellana).

##### Biology.

The holotype was collected by canopy fogging.

#### Coptoborus erwini sp. nov.

Taxon classificationAnimaliaColeopteraCurculionidae

D3EBFC9F-A608-5392-B183-F8092265672E

http://zoobank.org/59CF7571-B346-43EF-86CB-CA84A5DD630D

Figure 6A–C, M

##### Type material.

***Holotype*,** female, Ecuador: Napo Prov. [= Orellana Prov.], Tiputini Biodiversity Station, 00°37'55"S, 076°08'39"W, 220–250 m, February 1999, T.L. Erwin et al. collectors, insecticidal fogging, terra firme forest, trans[ect] 5, sta[tion] 7, Erwin lot #2046 (ICB). ***Paratypes***, female, as holotype except: Res[erva]. Ethnica Waorani, 1 km S. Okone Gare Camp, Trans[ect]. Ent[omology]., 00°39'10"S, 076°26'W, 220 m, January 1994, T.L. Erwin et al. collectors, insecticidal fogging, terra firme forest, trans[ect] 10, sta[tion] 3, Erwin lot #632 (ICB, 1); as previous except: January 1996, trans[ect] 2, sta[tion] 3, Erwin lot #1413 (NMNH, 1); as previous except: October 1996, trans[ect] 1, sta[tion] 7, Erwin lot #1667 (NMNH, 1); as previous except: trans[ect] 6, sta[tion] 6, Erwin lot #1716 (ICB, 1).

##### Diagnosis.

1.4–1.5 mm (mean = 1.45 mm; n = 4), 2.5–3.0 × as long as wide. This species is distinguished by the broadly rounded and entire elytral apex, posterolateral margin rounded, anterior margin of pronotum without serrations, and declivital interstrial setae about as long as the combined width of striae 1 and interstriae 1.

##### Similar species.

*C. doliolum*, *C. paurus*.

##### Description

**(female).** 1.4–1.5 mm (mean = 1.45 mm; n = 4), 2.5–3.0 × as long as wide (***holotype*** 1.4 mm, 2.8 × as long as wide). Body brown, antennae and legs lighter. ***Head***: epistoma smooth. Frons dull, finely punctate, setose; each puncture bearing a long, erect hair-like seta. Eyes narrowly and deeply emarginate. Submentum narrow, triangular, deeply impressed. Antennal scape short and thick, much shorter than club. Pedicel as long as funicle. Club longer than wide, flat, type 3; segment 1 corneous, transverse on anterior face, occupying basal ~1/3; segment 2 narrow, transverse, corneous; segments 1 and 2 present on posterior face. ***Pronotum***: 1.0–1.2 × as long as wide. In dorsal view basic and parallel-sided, type 2, sides parallel in basal 2/3, rounded anteriorly; anterior margin without serrations. In lateral view tall, type 2, disc flat, summit pronounced, on basal 2/5. Anterior slope with densely spaced, broad coarse asperities, becoming lower and more strongly transverse towards summit. Disc strongly shiny with sparse, minute punctures, some longer hair-like setae at margins. Lateral margins obliquely costate. ***Elytra***: 1.5–1.6 × as long as wide, 1.3 × as long as pronotum. Scutellum minute. Elytra round, parallel-sided in basal 75–78%, then broadly rounded to apex, apex entire. Disc shagreened, subshiny; strial punctures large, shallow, each bearing a recumbent seta the length of a puncture; interstriae flat, minutely and moderately punctate, unarmed, each puncture bearing a long semi-erect bristle-like seta. Declivity gradually rounded, occupying ~2/3 of elytra, smooth, shiny, declivital face convex; striae weakly impressed, strial punctures much larger, deeper than those of disc, each puncture bearing a semi-erect seta half as long as those of interstriae; interstriae flat, uniformly granulate along their entire lengths, granules small, interstriae 1–7 each with a row of long, erect setae as long as the combined width of striae 1 and interstriae 1. Posterolateral margin rounded. ***Legs***: protibiae semi-circular with evenly rounded outer margin, broadest at apical 1/3; apical 1/2 of outer margin with six large, socketed denticles, their length longer than basal width. Meso- and metatibiae flattened; outer margin evenly rounded with nine and eight large, socketed denticles, respectively.

##### Etymology.

Named after our colleague, the late Dr. Terry Erwin. Without his dedication to canopy fogging, this species and most of those described in this publication may never have been discovered. Noun in genitive.

##### Distribution.

Ecuador (Orellana).

##### Biology.

Specimens were collected by canopy fogging.

**Figure 6.**
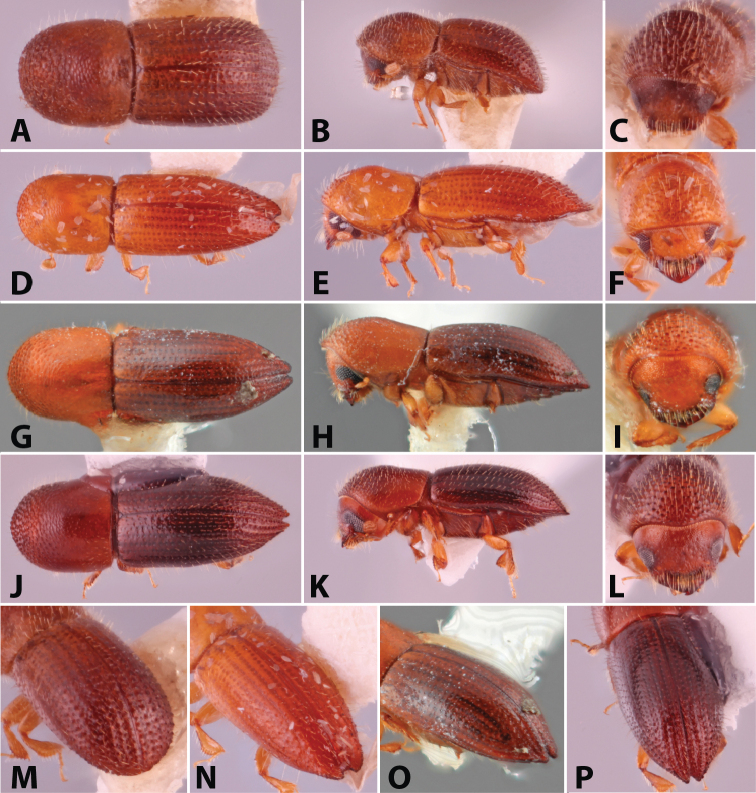
Dorsal, lateral, frontal and declivital view of *Coptoborus erwini* holotype, 1.4–1.5 mm (**A–C, M**), *C. exilis* 2.3–2.4 mm (**D–F, N**), *C. exutus* holotype, 1.9 mm (**G–I, O**), *C. furiosa* holotype, 2.1–2.2 mm (**J–L, P**). All photographs by SMS, **G–I, O** copyright National Museum of Natural History, Smithsonian Institution, Washington, D.C., published by permission.

#### Coptoborus exilis

Taxon classificationAnimaliaColeopteraCurculionidae

(Schedl, 1934) comb. nov.

C5D0F339-EF80-542A-BAB8-775216A6DB57

Figure 6D–F, N

Xyleborus exilis Schedl, 1934: 209.
Coptobrus exilis (Schedl): Wood and Bright 1992: 664 (as a synonym of C. pseudotenuis)
Xyleborus exilis Schedl: Bright 2019: 293.

##### Type material.

***Holotype*** (NHMW), examined.

##### New records.

Panama: Panamá Prov., [Parque Nacional Soberanía], Pipeline Rd, 9°7.975'N, 79°43.142'W, 174 m, 13.viii.2008, S.M. Smith, A.D. Smith, A.R. Gillogly, PAN 7, ex *Cecropia* (MSUC, 1); as previous except: Canal Zone, Barro Colorado [Island], 12.V.1980, Henk Wolda (UCDC, 1).

##### Diagnosis.

2.3–2.4 mm (mean = 2.35; n = 2), 3.29–3.43 × as long as wide. This species is distinguished by the elytral apex attenuate and weakly emarginate, declivital interstriae 2 convex, declivital interstriae 1 and 3 denticulate and interstriae 2 unarmed, declivital interstriae 3 with two or three enlarged denticles, denticles slightly larger than remaining interstriae 3 denticles, elytral apex deeply emarginate, and declivity gradual, occupying at least posterior 50% of declivity.

##### Similar species.

*C. artetenuis*, *C. pseudotenuis*.

##### Distribution.

Costa Rica (Puntarenas), Grenada, Panama (Panamá), Saint Lucia.

##### Biology.

This species is only known from *Cecropia* (Urticaceae).

##### Remarks.

The status of *C. exilis* has been surrounded by uncertainty for over 40 years. It has been considered a synonym of *C. pseudotenuis* (Wood 1982) and a ‘probable’ synonym of *C. pseudotenuis* (Wood 1976; Wood and Bright 1992). Wood (2007) later treated the species as valid and Bright (2019) returned this species to *Xyleborus* without discussion. This species belongs in *Coptoborus* because it shares the characters outlined in the generic diagnosis.

#### Coptoborus exutus

Taxon classificationAnimaliaColeopteraCurculionidae

(Wood, 1974)

5FE31ECD-6144-5522-A0BC-54F80159362D

Figure 6G–I, O

Xyleborus exutus Wood, 1974: 36.
Coptoborus exutus (Wood): Wood and Bright 1992: 663.

##### Type material.

***Holotyp*e** (NMNH), examined.

##### New records.

None.

##### Diagnosis.

1.9 mm, 3.0 × as long as wide (Wood 1982). This species is distinguished by the elytral apex attenuate and weakly emarginate, declivital interstriae 2 convex, declivital interstriae 1 and 3 denticulate and interstriae 2 unarmed, elytral apex deeply emarginate, and declivity steep, occupying less than posterior 40% of declivity.

##### Similar species.

*C. cuneatus*, *C. galacatosae*.

##### Distribution.

Costa Rica (Cartago).

##### Biology.

The holotype was collected from a fence post (Wood 1982).

#### Coptoborus furiosa sp. nov.

Taxon classificationAnimaliaColeopteraCurculionidae

8AFC50C5-7667-5B70-BD5C-3F0B76DA5372

http://zoobank.org/10BAA2AD-9CBD-4023-B5B9-A59EB6C6ED58

Figure 6J–L, P

##### Type material.

***Holotype*,** female, Ecuador: Los Ríos Prov., Canton La Clementina, Samama Nature Reserve, 01°38.852'S, 79°19.867'W, 381–430 m, 13–15.v.2015, Cognato, Smith, Osborn, Martinez et al., sample EC 15, ex 7 cm diameter bole from large tree fall (PUCE). ***Paratypes***, female, as holotype (MSUC, 1; PUCE, 1); as holotype except: sample EC 32, ex 3 cm dia. branches of tree fall (NMNH, 1: PUCE, 1).

##### Diagnosis.

2.1–2.2 mm (mean = 2.12 mm; n = 5), 3–3.14 × as long as wide. This species is distinguished by the elytral apex attenuate and strongly emarginate, declivity convex, declivital interstriae 2 denticulate, elytral apex with interstriae 3 and 9 joining, forming a crenulate carina that continues submarginally to apex, declivital interstriae 3 densely denticulate with more than ten denticles, and elytral apices sharply acute.

##### Similar species.

*C. inornatus*, *C. janeway*, *C. martinezae*, *C. tolimanus*, *C. vasquez*.

##### Description

**(female).** 2.1–2.2 mm (mean = 2.12 mm; n = 5), 3.0–3.14 × as long as wide (***holotype*** 2.1 mm, 3.0 × as long as wide). Body brown, elytra darker, antennae and legs lighter. ***Head***: epistoma smooth. Frons subshiny, finely punctate, setose; each puncture bearing a long, erect hair-like seta. Eyes broadly and moderately emarginate. Submentum narrow, triangular, deeply impressed. Antennal scape short and thick, much shorter than club. Pedicel shorter than funicle. Club longer than wide, flat, type 3; segment 1 corneous, convex on anterior face, occupying basal ~1/3; segment 2 narrow, transverse, corneous; segments 1 and 2 present on posterior face. ***Pronotum***: 1.1 × as long as wide. In dorsal view long and rounded frontally, type 7, sides parallel in basal 3/4, rounded anteriorly; anterior margin without serrations. In lateral view elongate, disc longer than anterior slope, type 7, summit prominent, on anterior 3/5. Anterior slope with densely spaced, broad fine asperities, becoming lower and more strongly transverse towards summit. Disc strongly shiny with sparse, minute punctures, some longer hair-like setae at margins. Lateral margins obliquely costate. ***Elytra***: 1.9 × as long as wide, 1.6 × as long as pronotum. Scutellum small. Elytra attenuate, parallel-sided in basal 62%, then acutely tapered to apex, apex acutely produced, strongly emarginate. Disc smooth, strongly shiny; strial punctures large, deep, glabrous; interstriae flat, sparsely, minutely punctate, unarmed, each puncture bearing a long semi-erect seta. Declivity gradual, occupying ~1/3 of elytra, smooth, shining, declivital face convex; striae not impressed, strial punctures larger, deeper than those of disc, each puncture bearing a semi-erect seta as long as two punctures; interstriae flat, interstriae denticulate along their entire lengths, interstriae 3 very densely denticulate, denticles separated by no more than the width of two denticles and with at least ten denticles, interstrial setae erect, bristle-like, uniseriate, interstriae 1 with an additional row of slightly shorter erect hair-like setae. Posterolateral margin with interstriae 3 and 9 joining, forming an acutely denticulate carina and continuing submarginally to apex. ***Legs***: protibiae obliquely triangular, broadest at apical 1/4; apical 1/2 of outer margin with five large, socketed denticles, their length longer than basal width. Meso- and metatibiae flattened; outer margin evenly rounded with six and seven large, socketed denticles, respectively.

##### Etymology.

Portrayed by Charlize Theron, Imperator Furiosa is the heroine in the movie ‘Mad Max: Fury Road’ (2015). The “spiny” elytra give the species a fierce appearance. Noun in apposition.

##### Distribution.

Ecuador (Los Ríos).

##### Biology.

This species was found in a bole and branches of an unidentified tree 3–7 cm in diameter.

#### Coptoborus galacatosae sp. nov.

Taxon classificationAnimaliaColeopteraCurculionidae

E4AC6AE2-E8F0-5F0C-BF4C-02D954430576

http://zoobank.org/C9B83469-B0F6-4429-BB0A-0E3E27E11939

Figure 7A–C, M

##### Type material.

***Holotype*,** female, Ecuador: Orellana Prov., Parque Nacional Yasuní ranger station, Tiputini, 11.vi.1996, A.I. Cognato, ex “Wabba” (MSUC). ***Paratypes***, female, as holotype (MSUC, 1; NMNH, 1).

##### Diagnosis.

1.75 mm (mean = 1.75 mm; n = 2), 2.92 × as long as wide. This species is distinguished by the elytral apex attenuate and weakly emarginate, declivital interstriae 2 convex, declivital interstriae 1 and 3 denticulate and interstriae 2 unarmed, elytral apex weakly emarginate, declivital striae 2 shallowly impressed.

##### Similar species.

*C. cuneatus*, *C. exutus*.

##### Description

**(female).** 1.75 mm (mean = 1.75 mm; n = 2), 2.92 × as long as wide (***holotype*** 1.75 mm, 2.92 × as long as wide). Body, antennae, and legs light brown. ***Head***: epistoma smooth. Frons subshiny, finely punctate, setose; each puncture bearing a long, erect hair-like seta. Eyes narrowly and deeply emarginate. Submentum large, triangular, slightly impressed. Antennal scape regularly thick, shorter than club. Pedicel as long as funicle. Club longer than wide, flat, type 3; segment 1 corneous, transverse on anterior face, occupying basal ~1/3; segment 2 narrow, transverse, corneous; segments 1 and 2 present on posterior face. ***Pronotum***: 1.0 × as long as wide. In dorsal view basic and parallel-sided, type 2, sides parallel in basal 4/5, rounded anteriorly; anterior margin without serrations. In lateral view uniformly rounded without a clear summit, type 1. Anterior slope with densely spaced, broad fine asperities, becoming lower and more strongly transverse towards summit. Disc strongly shiny with sparse, minute punctures, some longer hair-like setae at margins. Lateral margins obliquely costate. ***Elytra***: 1.67 × as long as wide, 1.7 × as long as pronotum. Scutellum minute. Elytra attenuate, parallel-sided in basal 70%, then acutely tapered to apex, apex weakly emarginate. Disc smooth, shiny; striae minutely punctate, glabrous; interstriae flat, sparsely, minutely punctate, unarmed, each puncture bearing a long semi-erect seta. Declivity gradually rounded, occupying ~1/3 of elytra, smooth, shiny, declivital face weakly convex; striae 2 very shallowly impressed, strial punctures larger than those of disc, glabrous, striae 1 parallel to suture; interstriae flat, interstriae 1 with two small denticles, interstriae 2 unarmed, interstriae 3 with three minute denticles, interstriae with a sparse row of erect bristle-like setae. Posterolateral margin with a poorly defined carina extending to interstriae 9 and composed of a few granules. ***Legs***: protibiae obliquely triangular, broadest at apical 1/3; apical 1/2 of outer margin with six large, socketed denticles, their length longer than basal width. Meso- and metatibiae flattened; outer margin evenly rounded with seven large, socketed denticles.

##### Etymology.

In gratitude of Dr. Katerina Galacatos who commanded several ichthyological and entomological expeditions to the remote reaches of the Yasuni River which provided AIC with his first Amazonian collecting trip. Noun in genitive.

##### Distribution.

Ecuador (Orellana).

##### Biology.

The type series was directly excised from wood of “Wabba”.

**Figure 7.**
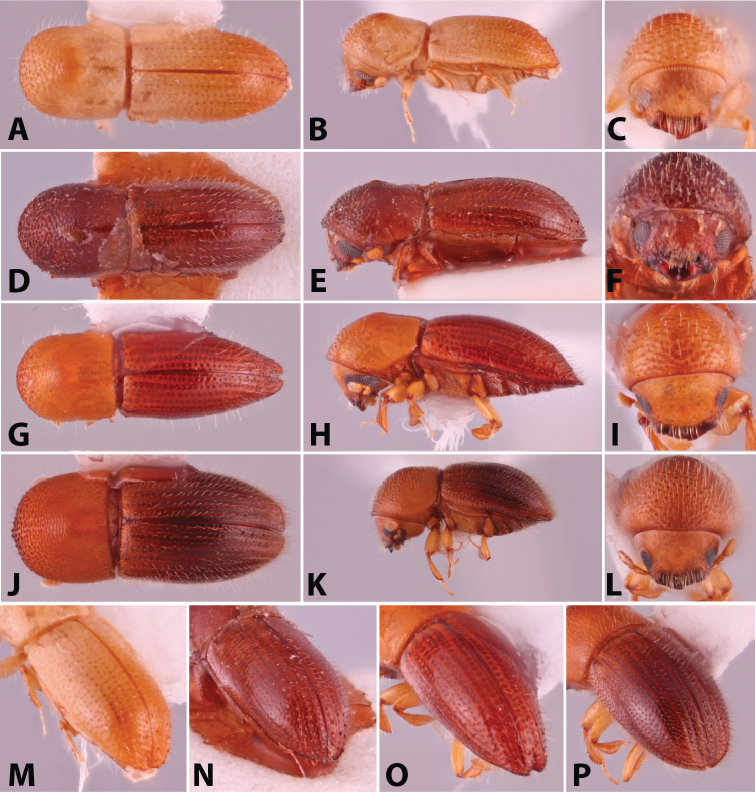
Dorsal, lateral, frontal and declivital view of *Coptoborus galacatosae* holotype, 1.75 mm (**A–C, M**), *C. gentilis* holotype, 2.3 mm (**D–F, N**), *C. gracilens* 2.4–2.5 mm (**G–I, O**), *C. hansen* holotype, 2.3 mm (**J–L, P**). All photographs by SMS.

#### Coptoborus gentilis

Taxon classificationAnimaliaColeopteraCurculionidae

(Schedl, 1972)

44B4FA72-D4F6-55E9-8ACB-C2A5D0258D69

Figure 7D–F, N

Xyleborus gentilis Schedl, 1972: 70.
Coptoborus gentilis (Schedl): Wood and Bright 1992: 663.

##### Type material.

***Holotype****Xyleborus gentilis* (NHMW), examined.

##### New records.

None.

##### Diagnosis.

2.3 mm (n = 1), 2.88 × as long as wide. This species is distinguished by the elytral apex attenuate, entire and produced, apical projection the width of striae 2, declivital interstriae 2 convex, declivital interstriae 1–3 denticulate, denticles distinct, their height equal to interstriae width, posterolateral margin of declivity with interstriae 3 and 9 joining, forming a carina and continuing submarginally to apex, and stout form.

##### Similar species.

*C. brevicauda*.

##### Distribution.

Brazil (Rio de Janeiro)

##### Biology.

Unknown.

#### Coptoborus gracilens

Taxon classificationAnimaliaColeopteraCurculionidae

Wood, 2007

ED81CC93-6770-5500-80D4-150EBBBFA8B0

Figure 7G–I, O

Coptoborus gracilens Wood, 2007: 401.

##### Type material.

***Holotype*** (MEFEIS), not examined. ***Paratypes*** (NMNH, 2), examined.

##### New records.

Brazil: Pará, Belém, Utinga, III-27–28, 1970, JM & BA Campbell (CNCI, 1). Ecuador: Napo Prov. [= Orellana Prov.], Res[erva]. Ethnica Waorani, 1 km S. Okone Gare Camp, Trans[ect]. Ent[omology]., 00°39'10"S, 076°26'W, 220 m, October 1996, T.L. Erwin et al. collectors, insecticidal fogging, terra firme forest, trans[ect] 3, sta[tion] 7, Erwin lot #91687 (ICB, 1). French Guiana: Crique Alma Maripasoula, 2°14'2.47"N, 54°27'0.19W, 12–20-VIII-2015, FIT with blue LED, E Poirier, P-H Dalens, F. Robin, Expedition “Our Planet Reviewed” Mitarka French Guiana 2015, MNHN/PNI & SEAG APA 973-1 (MSUC, 4). Peru: Madre de Dios Dept., CICRA Fld Stn., trail 6, research plot, 12.55207°S, 70.10962°W, 295 m, 11–13.VI.2011, Chaboo team, flight intercept trap PER-11-FIT-021 (SEMC, 1); as previous except: PER-11-FIT-027 (SEMC, 1); as previous except: PER-11-FIT-025 (SEMC, 13); as previous except: Los Amigos Biological Station, 12°34.9S, 70°6.04W, Smith, Hulcr, 26.iv.–2.v.2008, sample Peru 57, 7 cm diameter branch (MSUC, 4; MUSM, 1; NMNH, 3; NHMUK, 3; as previous except 3–9.v.2008, ex *Cecropia* 4 (MSUC, 2).

##### Diagnosis.

2.4–2.5 mm, 3.13–3.57 × as long as wide. This species is distinguished by the elytral apex attenuate and strongly emarginate, declivity convex, declivital interstriae 2 denticulate, elytral apex with interstriae 3 and 9 joining, forming a smooth continuous carina that continues submarginally to apex, apex produced, declivital interstriae 1 and 3 denticles subequal, and interstriae with long, erect hair-like setae at least twice as wide as interstrial width.

##### Similar species.

*C. cracens*, *C. leporinus*.

##### Distribution.

Brazil (Espírito Santo, Pará), Ecuador* (Orellana), French Guiana*, Peru* (Madre de Dios).

##### Biology.

This species has been collected from *Cecropia* (Urticaceae).

##### Remarks.

Wood (2007) incorrectly reported the holotype’s location as MZUSP. The holotype is in MEFEIS.

#### Coptoborus hansen sp. nov.

Taxon classificationAnimaliaColeopteraCurculionidae

96A4A411-84A1-5250-B271-6338DE71AFB9

http://zoobank.org/FBC54F3D-9CE6-4D98-A8DC-94BAF0B1B848

Figure 7J–L, P

##### Type material.

***Holotype*,** female, Brazil: Bahia, Camacan, Serra Bonita Reserve, 15°23.429'S, 39°33.810'W, 700–100 m, 6–14.V.2013, AI Cognato, SM Smith, CAH Flechtmann (MZUSP).

##### Diagnosis.

2.3 mm (n = 1), 2.6 × as long as wide. This species is distinguished by the elytral apex attenuate and entire and not produced, declivity interstriae 2 feebly sulcate, declivital interstriae 1–3 denticulate, denticles on interstriae 1 and 3 small and relatively indistinct, interstriae 1 unarmed on apical half, posterolateral margin of declivity with interstriae 3 and 9 joining, forming a carina and continuing submarginally to apex, stout form, and declivital interstriae and striae densely covered with abundant hair-like setae, setae uniformly fine from base to apex.

##### Similar species.

*C. barbicauda*, *C. bettysmithae*, *C. capillisoror*, *C. schulzi*, *C. subtilis*, *C. trinity*, *C. uhura*.

##### Description

**(female). *Holotype*** 2.3 mm, 2.6 × as long as wide. Body light brown, elytra darker, antennae and legs lighter. ***Head***: epistoma smooth. Frons dull, finely punctate, setose; each puncture bearing a long, erect hair-like seta. Eyes narrowly and deeply emarginate. Submentum narrow, triangular, slightly impressed. Antennal scape regularly thick, shorter than club. Pedicel shorter than funicle. Club longer than wide, flat, type 3; segment 1 corneous, transverse on anterior face, occupying basal ~1/4; segment 2 narrow, transverse, corneous; segments 1 and 2 present on posterior face. ***Pronotum***: 1.0 × as long as wide. In dorsal view basic and parallel-sided, type 2, sides parallel in basal 2/3, rounded anteriorly; anterior margin with two projecting serrations. In lateral view uniformly rounded without a clear summit, type 1. Anterior slope with densely spaced, narrow coarse asperities, becoming lower and more strongly transverse towards summit. Disc dull with dense, minute punctures, some longer hair-like setae at margins. Lateral margins obliquely costate. ***Elytra***: 1.4 × as long as wide, 1.4 × as long as pronotum. Scutellum minute. Elytra attenuate, parallel-sided in basal 62%, then acutely rounded to apex, apex entire. Disc smooth, shiny; strial punctures moderate, deep, each bearing a semi-erect hair-like seta the length of two punctures; interstriae flat, minutely, densely punctate, unarmed, each puncture bearing a long, erect bristle-like seta. Declivity gradually rounded, occupying ~1/2 of elytra, smooth, shiny, declivital face feebly sulcate; striae weakly impressed, strial punctures larger, deeper than those of disc, each puncture bearing a semi-recumbent hair-like seta as long as 3–5 punctures; interstriae flat, sparsely and inconsistently denticulate, denticles uniseriate, spaced by at least six widths of a denticle, apical half of interstriae 1 without denticles, interstriae 3 denticles faint, setae dense, long, erect, hair-like, 2–3 × as long as interstriae 1 width and uniform in thickness from base to apex, interstriae 1 with two additional rows of slightly shorter setae. Posterolateral margin with interstriae 3 and 9 joining, forming a carina, and continuing submarginally to apex. ***Legs***: protibiae obliquely triangular, broadest at apical 1/4; apical 1/2 of outer margin with eight large, socketed denticles, their length longer than basal width. Meso- and metatibiae flattened; outer margin evenly rounded with nine and eight large, socketed denticles, respectively.

##### Etymology.

Portrayed by Jeri Ryan, Annika Hansen (Seven of Nine as a Borg drone) is a heroine in the ‘Star Trek: Voyager’ (1995–2001) and ‘Star Trek: Picard’ (2020) television series. Noun in apposition.

##### Distribution.

Brazil (Bahia).

##### Biology.

Unknown.

#### Coptoborus incomptus sp. nov.

Taxon classificationAnimaliaColeopteraCurculionidae

80441226-15BB-5A28-A8B8-5F4BED8716BD

http://zoobank.org/E0C85F9C-B3D9-4D36-82E2-9952AA29B035

Figure 8A–C, M

##### Type material.

***Holotype*,** female, Peru: Madre de Dios Dept., Los Amigos Biological Station, 12°34.9S, 70°6.04W, Smith, Hulcr, 26.iv.–2.v.2008, sample Peru 2, branch (MUSM). ***Paratypes***, female, as holotype except: sample 50a, 9 cm diameter trunk (MUSM); as previous except: CM2, 12.4492°S, 70.2517°W, Smith, Hulcr, 17–18.v.2008, sample Peru 76, 3 cm diameter twig (MSUC, 1; NMNH, 1).

##### Diagnosis.

1.7–1.9 mm (mean = 1.8 mm; n = 4), 2.83–3.17 × as long as wide. This species is distinguished by the elytral apex attenuate and entire, declivital interstriae 1–3 denticulate, interstriae 2 with many fewer denticles than interstriae 1 or 3, declivital striae weakly impressed, antennal club obliquely truncate, type 2, segment 1 occupying basal 1/2, and posterolateral margin of declivity costate, armed with two large denticles.

##### Similar species.

*C. amplissimus*, *C. catulus*, *C. newt*, *C. scully*.

##### Description

**(female).** 1.7–1.9 mm (mean = 1.8 mm; n = 4), 2.83–3.17 × as long as wide (***holotype*** 1.7 mm, 2.83 × as long as wide). Body light brown, elytra darker, antennae and legs lighter. ***Head***: epistoma smooth. Frons dull, finely punctate, setose; each puncture bearing a long, erect hair-like seta. Eyes narrowly and deeply emarginate. Submentum narrow, triangular, deeply impressed. Antennal scape short and thick, as long as club. Pedicel shorter than funicle. Club circular, obliquely truncate, type 2; segment 1 corneous, transverse on anterior face, occupying basal ~2/5; segment 2 narrow, transverse, corneous; segments 1 and 2 present on posterior face. ***Pronotum***: 1.1–1.2 × as long as wide. In dorsal view long and rounded frontally, type 7, sides parallel in basal 4/5, rounded anteriorly; anterior margin without serrations. In lateral view elongate, disc longer than anterior slope, type 7, summit prominent, on anterior 2/3. Anterior slope with densely spaced, broad fine asperities, becoming lower and more strongly transverse towards summit. Disc dull with sparse, minute punctures, some longer hair-like setae at margins. Lateral margins obliquely costate. ***Elytra***: 1.8–1.9 × as long as wide, 1.8 × as long as pronotum. Scutellum small. Elytra attenuate, parallel-sided in basal 82–83%, then acutely tapered to apex, apex entire. Disc smooth, shiny; striae minutely punctate, glabrous; interstriae flat, sparsely, minutely punctate, unarmed, each puncture bearing a long, erect seta (typically abraded). Declivity steeply rounded, occupying ~1/4 of elytra, smooth, shiny, declivital face weakly convex; striae distinctly impressed, strial punctures larger, deeper than those of disc, glabrous, striae 1 irregular, slightly laterally broadened from base to declivital midpoint and then narrowing towards apex; interstriae flat, interstriae 1 and 3 each with three large denticles, interstriae 2 with two denticles, one at summit and one near apex, those of interstriae 1 and 3 subequal, much larger than those of interstriae 2, interstriae with a sparse row of erect bristle-like setae. Posterolateral margin costate, armed with two large denticles. ***Legs***: protibiae obliquely triangular, broadest at apical 1/3; apical 1/2 of outer margin with six large, socketed denticles, their length longer than basal width. Meso- and metatibiae flattened; outer margin evenly rounded with eight and seven large, socketed denticles, respectively.

##### Etymology.

L. *incomptus* = unadorned. In reference to the sparsely granulate declivity. Adjective.

##### Distribution.

Peru (Madre de Dios).

##### Biology.

The species has been collected from twigs and a trunk of an unknown tree 3–9 cm diameter.

**Figure 8.**
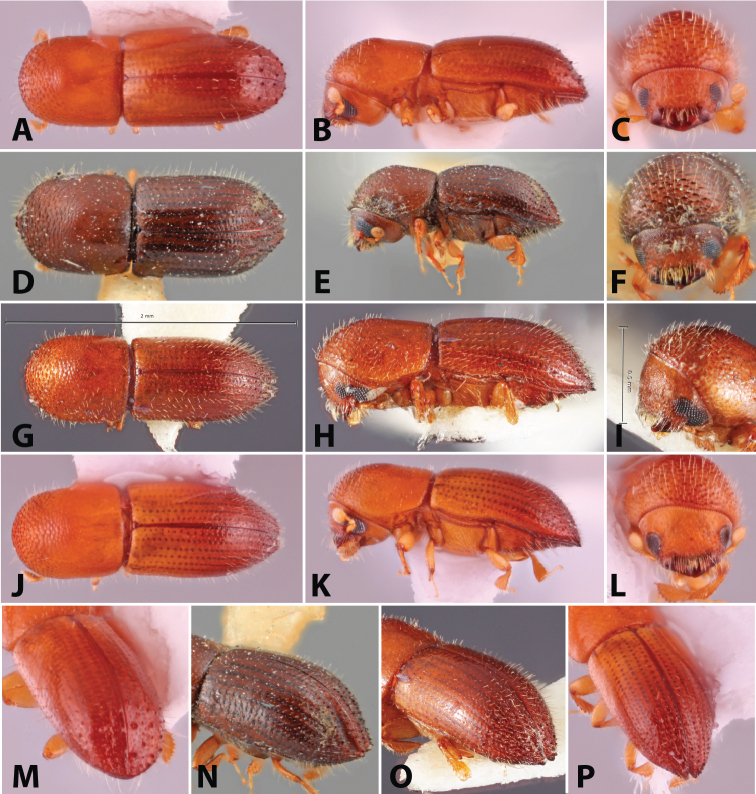
Dorsal, lateral, frontal and declivital view of *Coptoborus incomptus* holotype, 1.7–1.9 mm (**A–C, M**), *C. incultus* holotype, 2.3 mm (**D–F, N**), *C. inornatus* paratype, 1.8 mm (**G–I, O**), *C. janeway* holotype, 2.0 mm (**J–L, P**). All photographs by SMS except **G–I, O** by T.H. Atkinson, copyright National Museum of Natural History, Smithsonian Institution, Washington, D.C., published by permission.

#### Coptoborus incultus

Taxon classificationAnimaliaColeopteraCurculionidae

(Wood, 1975) comb. nov.

60B9B57D-31D5-52C8-ABC8-13ABF5E149A8

Figure 8D–F, N

Xyleborus incultus Wood, 1975b: 400.
Theoborus incultus (Wood): Wood 1982: 773.

##### Type material.

***Holotype*** (NMNH), examined.

##### New records.

None.

##### Diagnosis.

2.3 mm, 2.6 × as long as wide (Wood 1975b). This species is distinguished by the elytral apex attenuate and weakly emarginate, declivital interstriae 2 granulate only on basal third, declivital interstriae 2 strongly impressed, posterolateral margin of declivity with interstriae 3 and 9 joining, forming a carina and continuing submarginally to apex, declivital interstriae distinctly impressed, anterior margin of pronotum with a pair of projecting serrations.

##### Similar species.

*C. atlanticus*, *C. crassisororcula*.

##### Distribution.

Mexico (Campeche), Panama (Panamá).

##### Biology.

This species is only known from a *Cecropia* (Urticaceae) branch (Wood and Bright 1992).

#### Coptoborus inornatus

Taxon classificationAnimaliaColeopteraCurculionidae

Wood, 2007

F870FC3A-A930-5955-AF36-85BD485BFBF6

Figure 8G–I, O

Coptoborus inornatus Wood, 2007: 399.

##### Type material.

***Holotype*** (MEFEIS), not examined. ***Paratype*** (NMNH), examined.

##### New records.

None.

##### Diagnosis.

1.8 mm, 2.8 × as long as wide (Wood 2007). This species is distinguished by the elytral apex attenuate and strongly emarginate, declivity convex, declivital interstriae 2 denticulate, elytral apex with interstriae 3 and 9 joining, forming a crenulate carina that continues submarginally to apex, declivital interstriae 3 with fewer than ten denticles, elytral apices acute, declivital striae not impressed, elytral apex crenulations large and coarse, declivital slope steep, occupying 50% of elytra. It is most similar to *C. tolimanus* but has stouter elytra, 1.6 × as long as wide vs. 1.7–2.0 × as long as wide, and smaller size, 1.8 mm vs. 2.0–2.2 mm.

##### Similar species.

*C. furiosa*, *C. janeway*, *C. martinezae*, *C. tolimanus*, *C. vasquez*.

##### Distribution.

Brazil (Espírito Santo, Mato Grosso).

##### Biology.

Unknown.

##### Remarks.

Wood (2007) incorrectly reported the holotype’s location as MZUSP; the holotype is in MEFEIS. We examined the species description and images of a paratype but were unable to examine the holotype. Specimens of the type series do not share the same collecting event or locality. The holotype was collected from Mato Grosso, Brazil and the ten paratypes were collected in Espírito Santo, Brazil. The measurements of this species overlap almost entirely with those of *C. tolimanus* except in the elytral length width ratio, 1.6 × as long as wide vs. 1.7–2.0 × as long as wide. The body size is also likely identical given that Wood’s measurements were found to be 0.2 mm short (see methods). The slope of the elytra and elytral sculpturing is also identical. This species is almost certainly a synonym of *C. tolimanus* but the holotype should be examined to ensure that its morphology is congruent with that of the paratypes before it is formally synonymized.

#### Coptoborus janeway sp. nov.

Taxon classificationAnimaliaColeopteraCurculionidae

8C08E883-E5E7-56E1-9190-BCA306709056

http://zoobank.org/E9531FB9-A90A-4CE8-984A-631CDD36B0C5

Figure 8J–L, P

##### Type material.

***Holotype*,** female, Peru: Madre de Dios Dept., Los Amigos Biological Station, CM2, GPS 12.4492°S, 70.2517°W, Smith, Hulcr, 17–18.v.2008, sample Peru 83b 8 cm diameter branch (MUSM). ***Paratypes***, female, as holotype (MSUC, 1); as holotype except: sample Peru 96c 1 cm diameter branch (MSUC, 1).

##### Diagnosis.

2.0 mm (mean = 2.0 mm; n = 3), 2.86 × as long as wide. This species is distinguished by the elytral apex attenuate and strongly emarginate, declivity convex, declivital interstriae 2 denticulate, elytral apex with interstriae 3 and 9 joining, forming a crenulate carina that continues submarginally to apex, declivital interstriae 3 with fewer than ten denticles, elytral apices obtuse, declivital striae not impressed, elytral apex crenulations small and fine, and declivital slope gradual, occupying 57% of elytral length.

##### Similar species.

*C. furiosa*, *C. inornatus*, *C. martinezae*, *C. tolimanus*, *C. vasquez*.

##### Description

**(female). *Holotype*** 2.0 mm, 2.86 × as long as wide. Body light brown, elytra darker, antennae and legs lighter. ***Head***: epistoma tuberculate. Frons subshiny, finely punctate, setose; each puncture bearing a long, erect hair-like seta. Eyes narrowly and deeply emarginate. Submentum narrow, triangular, deeply impressed. Antennal scape short and thick, as long as club. Pedicel shorter than funicle. Club circular, obliquely truncate, type 2; segment 1 corneous, convex on anterior face, occupying basal ~2/5; segment 2 narrow, convex, corneous; segments 1 and 2 present on posterior face. ***Pronotum***: 1.1 × as long as wide. In dorsal view long and rounded frontally, type 7, sides parallel in basal 2/3, rounded anteriorly; anterior margin without serrations. In lateral view elongate, disc longer than anterior slope, type 7, summit prominent, on anterior 2/3. Anterior slope with densely spaced, broad fine asperities, becoming lower and more strongly transverse towards summit. Disc strongly shiny with moderately dense, minute punctures, some longer hair-like setae at margins. Lateral margins obliquely costate. ***Elytra***: 1.7 × as long as wide, 1.5 × as long as pronotum. Scutellum small. Elytra attenuate, parallel-sided in basal 63%, then acutely tapered to apex, apex weakly emarginate. Disc smooth, shiny; striae minutely punctate, glabrous; interstriae flat, sparsely, minutely punctate, unarmed, glabrous. Declivity gradual, occupying ~1/3 of elytra, smooth, shiny, declivital face convex; striae not impressed, strial punctures larger, deeper than those of disc, each puncture bearing a semi-erect seta as long as two punctures; interstriae flat, interstriae densely denticulate along their entire lengths, separated by at least the width of four denticles, interstrial setae erect, thick, bristle-like, uniseriate, interstriae 1 with two additional rows of shorter erect hair-like setae. Posterolateral margin with interstriae 3 and 9 joining, forming a minutely denticulate carina and continuing submarginally to apex. ***Legs***: protibiae obliquely triangular, broadest at apical 1/3; apical 2/3 of outer margin with six large, socketed denticles, their length longer than basal width. Meso- and metatibiae flattened; outer margin evenly rounded with eight and seven large, socketed denticles, respectively.

##### Etymology.

Portrayed by Kate Mulgrew, Captain Kathryn Janeway is the heroine in the television series ‘Star Trek: Voyager’ (1995–2001). Noun in apposition.

##### Distribution.

Peru (Madre de Dios).

##### Biology.

The species has been collected from branches and twigs of unidentified trees 1–8 cm in diameter.

#### Coptoborus katniss sp. nov.

Taxon classificationAnimaliaColeopteraCurculionidae

55B55612-E1F9-50EB-A301-AE5F994C9772

http://zoobank.org/69ABA8D7-DA4D-4BF0-B3EF-89E3E8D87DA2

Figure 9A–C, M

##### Type material.

***Holotype*,** female, Ecuador: [Sucumbíos Prov.], Limoncocha, 0°23'S, 76°38"W, 300 m, 31.iii.1974, H.P. Stockwell (TAMU).

##### Diagnosis.

2.7 mm (n = 1), 2.7 × as long as wide. This species is distinguished by the elytral apex strongly acuminate, declivital interstriae unarmed along its entire length, antennal club with two sutures on posterior face, elytral discal interstriae impunctate, declivity nearly devoid of granules except for interstriae 1 and 3 on acuminate projection, and declivity with a carina extending from apex to interstriae 2.

##### Similar species.

*C. attenuatus*, *C. bellus*, *C. sagitticauda*, *C. sarahconnor*, *C. sicula*, *C. yar*.

##### Description

**(female). *Holotype*** 2.7 mm, 2.7 × as long as wide. Body uniformly brown. ***Head***: epistoma smooth. Frons subshiny, finely punctate, setose; each puncture bearing a long, erect hair-like seta. Eyes narrowly and deeply emarginate. Antennal scape regularly thick, as long as club. Club circular, flat, type 3; segment 1 corneous, transverse on anterior face, occupying basal ~2/5; segment 2 narrow, transverse, corneous; segments 1 and 2 present on posterior face. ***Pronotum***: 1.2 × as long as wide. In dorsal view long and rounded frontally, type 7, sides parallel in basal 2/3, rounded anteriorly; anterior margin with four projecting serrations, median pair larger. In lateral view tall, type 2, disc flat, summit pronounced, at midpoint. Anterior slope with densely spaced, broad fine asperities, becoming lower and more strongly transverse towards summit. Disc strongly reticulate, dull with sparse, minute punctures, some longer hair-like setae at margins. Lateral margins entirely carinate. ***Elytra***: 1.5 × as long as wide, 1.25 × as long as pronotum. Scutellum minute. Elytra attenuate, parallel-sided in basal 2/3, then acutely narrowed to acuminate apex. Disc shagreened, shiny; strial punctures minute, each bearing a recumbent hair-like seta the length of three punctures; interstriae flat, impunctate, unarmed, glabrous. Declivity gradually rounded, occupying ~2/5 of elytra, shagreened, dull, declivital face weakly convex; striae not impressed, strial punctures larger, deeper than those of disc, each puncture bearing a recumbent hair-like seta as long as two punctures; interstriae flat, nearly devoid of granules except for interstriae 1 and 3 on acuminate projection, interstriae with a row of short erect hair-like setae. Posterolateral margin with a very short carina extending from apex to interstriae 2. ***Legs***: protibiae distinctly triangular, broadest at apical 1/5; apical 1/2 of outer margin with six large, socketed denticles, their length longer than basal width. Meso- and metatibiae flattened; outer margin evenly rounded with seven large, socketed denticles.

##### Etymology.

Portrayed by Jennifer Lawrence, Katniss Everdeen is a heroine in the ‘The Hunger Games’ movie franchise (2008–2010). The apex of the elytra declivity of this species is shaped like an arrowhead, Katniss’ weapon of choice. Noun in apposition.

##### Distribution.

Ecuador (Sucumbíos).

##### Biology.

Unknown.

**Figure 9.**
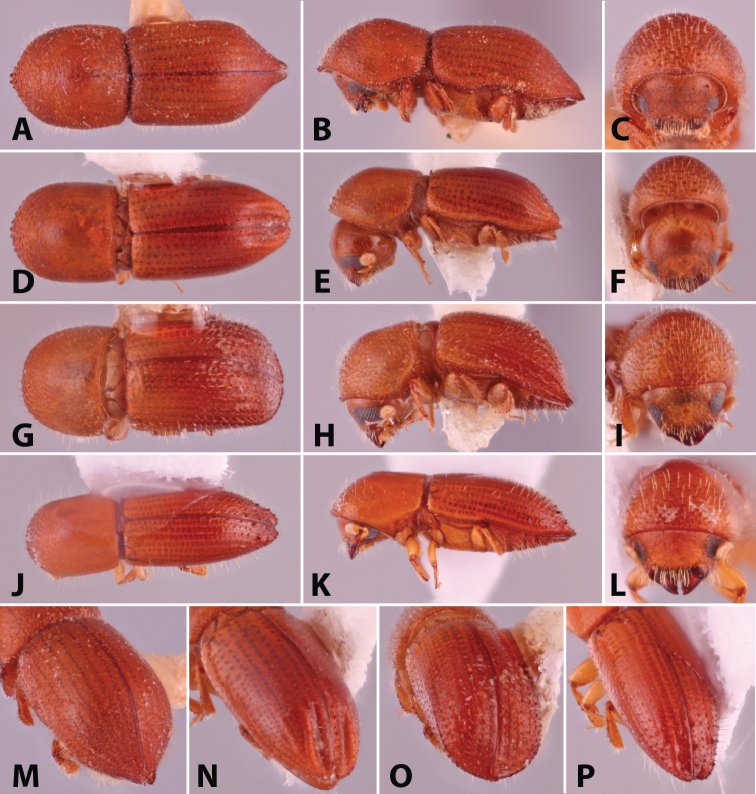
Dorsal, lateral, frontal and declivital view of *Coptoborus katniss* holotype, 2.7 mm (**A–C, M**), *C. leeloo* holotype, 1.6–1.7 mm (**D–F, N**), *C. leia* holotype, 2.0 mm (**G–I, O**), *C. leporinus* holotype, 2.35 mm (**J–L, P**). All photographs by SMS.

#### Coptoborus leeloo sp. nov.

Taxon classificationAnimaliaColeopteraCurculionidae

4FA0728D-15FD-5BAD-94CE-ACB28B77F1F4

http://zoobank.org/E3D3681B-8A81-4532-8F1A-3EF4450B12F8

Figure 9D–F, N

##### Type material.

***Holotype*,** female, Ecuador: Napo Prov. [= Orellana Prov.], Res[erva]. Ethnica Waorani, 1 km S. Okone Gare Camp, Trans[ect]. Ent[omology]., 00°39'10"S, 076°26'W, 220 m, January1996, T.L. Erwin et al. collectors, insecticidal fogging, terra firme forest, trans[ect] 2, sta[tion] 1, Erwin lot #1411 (ICB). ***Paratypes***, female, as holotype (ICB, 1); as holotype except: Tiputini Biodiversity Station, 00°37'55"S, 076°08'39"W, 220–250 m, June 1998, T.L. Erwin et al. collectors, insecticidal fogging, terra firme forest, trans[ect] 6, sta[tion] 2, Erwin lot #1851 (ICB, 1; NMNH, 2).

##### Diagnosis.

1.6–1.7 mm (mean = 1.65 mm; n = 4), 2.67–2.83 × as long as wide. This species is distinguished by the elytral apex attenuate and weakly emarginate, declivity distinctly sulcate along interstriae 2, declivital interstriae 2 unarmed, interstriae 1 and 3 armed, declivital striae 1 and 2 not parallel on declivital face, nearly converging in sulcate area, and minute size.

##### Similar species.

*C. busoror*, *C. nudulus*, *C. ochromactonus*, *C. pilisoror*, *C. ripley*, *C. sororcula*, *C. spicatus*.

##### Description

**(female).** 1.6–1.7 mm (mean = 1.65 mm; n = 4), 2.67–2.83 × as long as wide (***holotype*** 1.6 mm, 2.67 × as long as wide). Body light brown, antennae and legs lighter. ***Head***: epistoma smooth. Frons subshiny, finely punctate, setose; each puncture bearing a long, erect hair-like seta. Eyes broadly and moderately emarginate. Submentum large, triangular, deeply impressed. Antennal scape long and slender, shorter than club. Pedicel shorter than funicle. Club circular, flat, type 3; segment 1 corneous, transverse on anterior face, occupying basal ~1/3; segment 2 narrow, corneous; segments 1 and 2 present on posterior face. ***Pronotum***: 1.2 × as long as wide. In dorsal view long and rounded frontally, type 7, sides parallel in basal 3/5, rounded anteriorly; anterior margin with two projecting serrations. In lateral view elongate, disc longer than anterior slope, type 7, summit prominent, on anterior 3/5. Anterior slope with densely spaced, broad coarse asperities, becoming lower and more strongly transverse towards summit. Disc reticulate, dull with sparse, minute punctures, some longer hair-like setae at margins. Lateral margins carinate on basal third. ***Elytra***: 1.5–1.7 × as long as wide, 1.3 × as long as pronotum. Scutellum minute. Elytra attenuate, parallel-sided in basal 67–70%, then acutely tapered to apex, apex weakly emarginate. Disc smooth shiny; strial punctures moderate, shallow, each bearing a recumbent seta the length of a puncture; interstriae flat, sparsely, minutely punctate, unarmed, each puncture bearing a recumbent seta the length of a puncture. Declivity gradual, smooth, shiny, appearing bisulcate, occupying apical 2/5 of elytra; striae not impressed, striae 1 and 2 nearly converging in sulcate area, strial punctures much larger and shallower than those of disc; interstriae impunctate, interstriae 2 moderately sulcate, unarmed; interstriae 1 and 3 strongly costate with eight small granules, each puncture bearing a short recumbent seta. Posterolateral margin with interstriae 3 and 9 joining, forming a costa and continuing submarginally to apex. ***Legs***: protibiae obliquely triangular, broadest at apical 1/4; apical 1/2 of outer margin with six large, socketed denticles, their length longer than basal width. Meso- and metatibiae flattened; outer margin evenly rounded with seven and six moderately sized socketed denticles, respectively, their length as large as basal width.

##### Etymology.

Portrayed by Milla Jovovich, Leeloo is the heroine in the movie ‘The Fifth Element’ (1997). Noun in apposition.

##### Distribution.

Ecuador (Orellana).

##### Biology.

Specimens were collected by canopy fogging.

#### Coptoborus leia sp. nov.

Taxon classificationAnimaliaColeopteraCurculionidae

A308E71D-81B5-5045-A944-C15C7415889E

http://zoobank.org/E629D059-A21E-494B-B742-8D187C6823B3

Figure 9G–I, O

##### Type material.

***Holotype*,** female, Ecuador: Napo Prov. [= Orellana Prov.], Tiputini Biodiversity Station, 00°37'55"S, 076°08'39"W, 220–250 m, June 1998, T.L. Erwin et al. collectors, insecticidal fogging, terra firme forest, trans[ect] 10, sta[tion] 4, Erwin lot #1893 (ICB). ***Paratype***, female, Suriname: Sipaliwini, 2.977312°N, 55.38500°W, 200 m, Camp 4 (low), Kasikasima, T. Larsen, 20–25.iii.2012, FIT, SR12-0320-TN1, 2012 CI-RAP survey (NZCS, 1).

##### Diagnosis.

2.0 mm (mean = 2.0 mm; n = 2), 2.2 × as long as wide. This species is distinguished by the elytral apex broadly rounded and entire, posterolateral margin continuously and smoothly carinate to striae 6 and not extended posteriad, declivital interstrial setae fine hair-like, shorter than the width of interstriae 2 and moderately covering declivity, declivital interstriae minutely granulate, and declivital striae 1 and 2 feebly impressed.

##### Similar species.

*C. brigman*, *C. tristiculus*, *Euwallacea perbrevis*.

##### Description

**(female). *Holotype*** 2.0 mm, 2.2 × as long as wide. Body light brown, antennae and legs lighter. ***Head***: epistoma smooth. Frons strongly shiny, finely punctate, setose; each puncture bearing a long, erect hair-like seta. Eyes narrowly and moderately emarginate. Submentum narrow, triangular, deeply impressed. Antennal scape regularly thick, much shorter than club. Pedicel shorter than funicle. Club longer than wide, flat, type 4; segment 1 corneous, transverse on anterior face, occupying basal ~1/5; segment 2 narrow, transverse, corneous; segments 1 and 2 present on posterior face. ***Pronotum***: 0.9 × as long as wide. In dorsal view basic and parallel-sided, type 2, sides parallel in basal 5/7, rounded anteriorly, abundantly covered with long hair-like setae; anterior margin with four projecting serrations, median pair larger than lateral pair. In lateral view uniformly rounded without a clear summit, type 1. Anterior slope with densely spaced, broad coarse asperities, becoming lower and more strongly transverse towards summit. Disc strongly shiny with sparse, minute punctures. Lateral margins obliquely costate. ***Elytra***: 1.3 × as long as wide, 1.5 × as long as pronotum. Scutellum small. Elytra round, parallel-sided in basal 83%, then broadly rounded to apex, apex entire. Disc smooth, shiny; strial punctures large, deep, each bearing a recumbent hair-like seta the length of two punctures; interstriae flat, minutely, moderately punctate, unarmed, each puncture bearing a long semi-erect bristle-like seta. Declivity gradually rounded, occupying ~1/3 of elytra, smooth, shiny, declivital face flattened; striae 1 and 2 feebly impressed, strial punctures larger, deeper than those of disc, each puncture bearing a semi-erect seta as long as two punctures; interstriae flat, uniformly minutely granulate along their entire lengths, setae fine, hair-like, shorter than the width of interstriae 2. Posterolateral margin continuously and smoothly carinate to striae 6. ***Legs***: protibiae obliquely triangular, broadest at apical 1/4; apical 1/2 of outer margin with eight large, socketed denticles, their length longer than basal width. Meso- and metatibiae flattened; outer margin evenly rounded with ten and eight large, socketed denticles, respectively.

##### Etymology.

Portrayed by Carrie Fisher, Princess Leia Organa is the heroine in the ‘Star Wars’ movies IV–IX (1977–2019). The species is setose and round like the character’s bun-styled hair. Noun in apposition.

##### Distribution.

Ecuador (Orellana), Suriname.

##### Biology.

The holotype was collected by canopy fogging.

#### Coptoborus leporinus sp. nov.

Taxon classificationAnimaliaColeopteraCurculionidae

8E88B800-EA6B-55A2-A68D-83182BC43539

http://zoobank.org/E60598E6-2BEB-4822-BA2F-DA25DDFE7A70

Figure 9J–L, P

##### Type material.

***Holotype*,** female, Peru: Madre de Dios Dept., Los Amigos Biological Station, CM2, 12.4492°S, 70.2517°W, Smith, Hulcr, 17–18.v.2008, sample Peru 76, 3 cm diameter twig (MUSM).

##### Diagnosis.

2.35 mm (n = 1), 3.36 × as long as wide. This species is distinguished by the elytral apex attenuate and strongly emarginate, declivity convex, declivital interstriae 2 denticulate, elytral apex with interstriae 3 and 9 joining, forming a smooth continuous carina that continues submarginally to apex, apex produced, declivital interstriae 3 denticles much larger than those of interstriae 1, and interstriae with short erect bristle-like setae that are at least twice as wide as interstrial width.

##### Similar species.

*C. cracens*, *C. gracilis*.

##### Description

**(female). *Holotype*** 2.35 mm, 3.36 × as long as wide. Body light brown, antennae and legs lighter. ***Head***: epistoma smooth. Frons dull, finely punctate, setose; each puncture bearing a long, erect hair-like seta. Eyes broadly and moderately emarginate. Submentum narrow, triangular, slightly impressed. Antennal scape short and thick, as long as club. Pedicel shorter than funicle. Club circular, flat, type 3; segment 1 corneous, subconvex on anterior face, occupying basal 1/3; segment 2 narrow, transverse, corneous; segments 1 and 2 present on posterior face. ***Pronotum***: 1.3 × as long as wide. In dorsal view long and rounded frontally, type 7, sides parallel in basal 3/4, rounded anteriorly; anterior margin without serrations. In lateral view elongate, disc longer than anterior slope, type 7, summit prominent, on anterior 3/5. Anterior slope with densely spaced, broad fine asperities, becoming lower and more strongly transverse towards summit. Disc reticulate, dull with sparse, minute punctures, some longer hair-like setae at margins. Lateral margins obliquely costate. ***Elytra***: 2.1 × as long as wide, 1.6 × as long as pronotum. Scutellum small. Elytra attenuate, parallel-sided in basal 62%, then acutely tapered to apex, apex strongly emarginate and apically produced. Disc smooth, shiny; striae minutely punctate, glabrous; interstriae flat, sparsely, minutely punctate, unarmed, glabrous. Declivity gradual, occupying ~2/5 of elytra, smooth, shiny, declivital face convex; striae distinctly impressed, strial punctures larger, deeper than those of disc, each puncture bearing a semi-recumbent seta as long as two punctures; interstriae 2 weakly sulcate, interstriae 1 and 3 each with three and five denticles, respectively, those on interstriae 3 the largest, interstriae 2 with three minute denticles on basal third, interstrial setae erect, thick, bristle-like, uniseriate. Posterolateral margin with interstriae 3 and 9 joining, forming a granulate carina and continuing submarginally to apex. ***Legs***: protibiae obliquely triangular, broadest at apical 1/4; apical 1/2 of outer margin with six large, socketed denticles, their length longer than basal width. Meso- and metatibiae flattened; outer margin evenly rounded with seven and six large, socketed denticles, respectively.

##### Etymology.

L. *leporinus* = of hares. In reference to the appearance of rabbit ears when the elytral apex is viewed from a dorsal profile. Adjective.

##### Distribution.

Peru (Madre de Dios).

##### Biology.

This species has been collected from a 3 cm diameter twig of an unidentified tree.

#### Coptoborus magnus

Taxon classificationAnimaliaColeopteraCurculionidae

(Petrov, 2020) comb. nov.

2D05E522-AFCA-54D2-A570-39FB07C7DC81

Figure 10A–C, M

Theoborus magnus Petrov 2020: 408.

##### Type material.

***Holotype*** (ZMMU), examined.

##### Diagnosis.

3.1 mm (mean = 3.1 mm; n = 2), 2.17 × as long as wide (Petrov 2020). This species is distinguished by the elytra attenuate, apex entire, elytra excavated between interstriae 3 and much more strongly impressed on basal half, anterior margin of pronotum with a pair of projecting serrations, disc occupying 80% of elytral length, and large size, 3.1 mm, and stout form, 2.17 × as long as wide.

##### Similar species.

*C. amazonicus*.

##### Distribution.

Peru (Loreto).

##### Biology.

Unknown.

**Figure 10.**
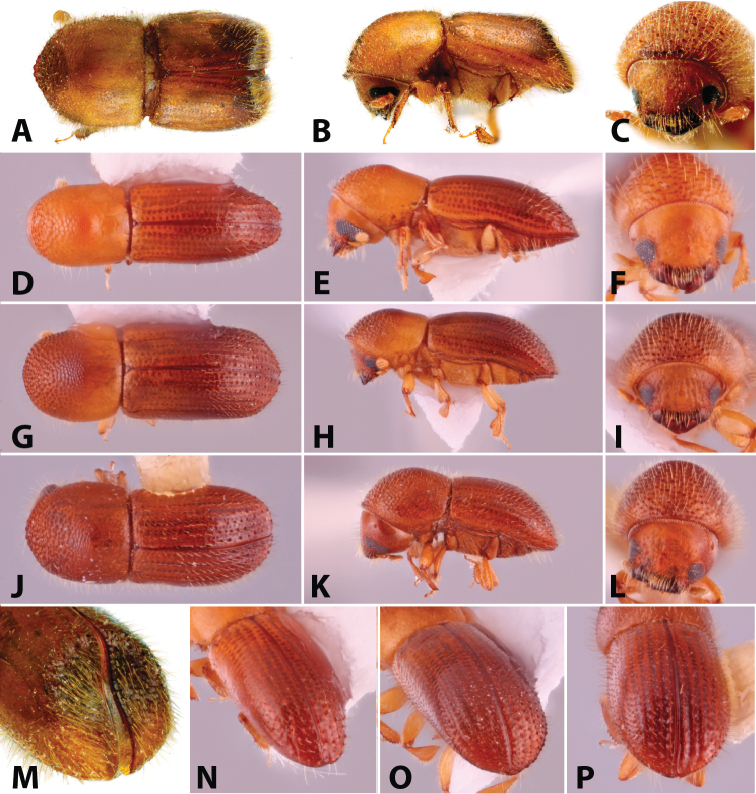
Dorsal, lateral, frontal and declivital view of *Coptoborus magnus* holotype, 3.1 mm (**A–C, M**), *C. martinezae* holotype, 2.0–2.1 mm (**D–F, N**), *C. micarius*, 2.0–2.4 mm (**G–I, O**), *C. murinus* holotype, 2.2 mm (**J–L, P**). All photographs by SMS except (**A–C, M)** by A.V. Petrov.

#### Coptoborus martinezae sp. nov.

Taxon classificationAnimaliaColeopteraCurculionidae

BE02C721-76B5-5ED7-A009-4B5BF7B7D57A

http://zoobank.org/136416C9-8696-4843-8D6F-AE3667F7AF10

Figure 10D–F, N

##### Type material.

***Holotype*,** female, Ecuador: Los Ríos, Canton La Clementina, Samama Nature Reserve, 01°38.852'S, 79°19.867'W, 381–430 m, 13–15.v.2015, Cognato, Smith, Osborn, Martinez et al., sample EC 30, ex buttressed tree, 30 cm DBH (PUCE). ***Paratypes***, female, as holotype (MSUC, 1; PUCE, 1); as holotype except: EC 14 (MSUC, 12; NHMUK, 5; NMNH, 5; PUCE, 5); as holotype except: EC 13, ex 4 cm diameter hanging liana (MSUC, 6; NMNH, 5; NHMUK, 5; PUCE, 5); as holotype except: EC 15, ex 7 cm diameter branch from large tree fall (MSUC, 6; NHMH, 3; PUCE, 3); as holotype except: EC 25, ex 2 cm diameter branches along trail (MSUC, 1; PUCE, 1); as holotype except: EC 32, ex 3 cm diameter branches at tree fall (MSUC, 2; PUCE, 1).

##### Diagnosis.

2.0–2.1 mm (mean = 2.04 mm; n = 5), 3.0–3.5 × as long as wide. This species is distinguished by the elytral apex attenuate and strongly emarginate, declivity convex, declivital interstriae 2 denticulate, elytral apex with interstriae 3 and 9 joining, forming a smooth continuous carina that continues submarginally to apex, and apex not produced.

##### Similar species.

*C. furiosa*, *C. inornatus*, *C. janeway*, *C. tolimanus*, *C. vasquez*.

##### Description

**(female).** 2.0–2.1 mm (mean = 2.04 mm; n = 5), 3.0–3.5 × as long as wide (***holotype*** 2.0 mm, 3.33 × as long as wide). Body light brown, elytra darker, antennae and legs lighter. ***Head***: epistoma smooth. Frons subshiny, finely punctate, setose; each puncture bearing a long, erect hair-like seta. Eyes broadly and moderately emarginate. Submentum narrow, triangular, slightly impressed. Antennal scape short and thick, as long as club. Pedicel shorter than funicle. Club circular, obliquely truncate, type 2; segment 1 corneous, transverse on anterior face, occupying basal ~2/5; segment 2 narrow, transverse, corneous; segments 1 and 2 present on posterior face. ***Pronotum***: 1.1–1.3 × as long as wide. In dorsal view long and rounded frontally, type 7, sides parallel in basal 2/3, rounded anteriorly; anterior margin without serrations. In lateral view elongate, disc as long as anterior slope, type 7, summit prominent. Anterior slope with densely spaced, broad fine asperities, becoming lower and more strongly transverse towards summit. Disc strongly shiny with sparse, minute punctures, some longer hair-like setae at margins. Lateral margins obliquely costate. ***Elytra***: 2.0–2.2 × as long as wide, 1.5 × as long as pronotum. Scutellum small. Elytra attenuate, parallel-sided in basal 64–69%, then acutely tapered to apex, apex strongly emarginate. Disc smooth, shiny; striae minutely punctate, glabrous; interstriae flat, sparsely, minutely punctate, unarmed, each puncture bearing a long, erect seta. Declivity gradual, occupying ~1/3 of elytra, smooth, shiny, declivital face convex; striae not impressed, strial punctures larger, deeper than those of disc, each puncture bearing a recumbent seta as long as two punctures; interstriae flat, interstriae sparsely denticulate along their entire lengths, interstriae 1 and 3 much larger than those of interstriae 2, interstrial setae erect, thick, bristle-like, uniseriate. Posterolateral margin with interstriae 3 and 9 joining, forming a granulate carina and continuing submarginally to apex. ***Legs***: protibiae obliquely triangular, broadest at apical 1/3; apical 1/2 of outer margin with six large, socketed denticles, their length longer than basal width. Meso- and metatibiae flattened; outer margin evenly rounded with seven large, socketed denticles.

##### Etymology.

In recognition of Dr. Malena Martinez’s collaboration in the study of xyleborines and their symbiotic fungi. Noun in genitive.

##### Distribution.

Ecuador (Los Ríos).

##### Biology.

The species was found in 2–30 cm diameter branches of unidentified trees.

#### Coptoborus micarius

Taxon classificationAnimaliaColeopteraCurculionidae

(Wood, 1974) comb. nov.

CF21427B-7642-5239-B7A3-46FBB2009E89

Figure 10G–I, O

Xyleborus micarius Wood, 1974: 33.
Theoborus micarius (Wood): Wood 1982: 774.

##### Type material.

***Holotype*** (NMNH), ***paratypes*** (NMNH, 3), examined.

##### New records.

Panama: Chiriquí, Fortuna, 8°24.840'N, 82°14.562'W, 1150 m, SM Smith, AD Smith, AR Gillogly, 29.viii.2008, PAN102 (MSUC, 1).

##### Diagnosis.

2.0–2.4 mm, 2.67–2.86 × as long as wide. This species is distinguished by the elytral apex broadly rounded and entire, posterolateral margin distinctly carinate to striae 6, carina unequally serrate and appearing broken, serrations on interstriae 1 and 2 with acute apices that are less than 2 × the size of other serrations.

##### Similar species.

*C. pristis*.

##### Distribution.

Costa Rica (Cartago), Panama* (Chiriquí).

##### Biology.

This species is only known from *Cordia* (Cordiaceae) (Wood and Bright 1992). Wood (1982) noted that the species was collected attacking branches 5–7 cm in diameter.

#### Coptoborus murinus sp. nov.

Taxon classificationAnimaliaColeopteraCurculionidae

1862D9B2-6F4A-569B-B919-DE42D1E7F8AD

http://zoobank.org/B0CF7A83-65B2-4184-8B3E-BEEB53D629A4

Figure 10J–L, P

##### Type material.

***Holotype*,** female, Ecuador: Napo Prov. [= Orellana Prov.], Tiputini Biodiversity Station, 00°37'55"S, 076°08'39"W, 220–250 m, June 1998, T.L. Erwin et al. collectors, insecticidal fogging, terra firme forest, trans[ect] 5, sta[tion] 4, Erwin lot #1843 (ICB).

##### Diagnosis.

2.2 mm (n = 1), 2.44 × as long as wide. This species is distinguished by the elytral apex broadly rounded and entire, posterolateral margin feebly carinate to striae 6, primarily visible between suture and striae 2, unequally serrate and appearing broken, declivity gradual, occupying posterior half of elytra, and declivital interstrial setae twice as long as interstriae 1 width.

##### Similar species.

*C. osbornae*.

##### Description

**(female). *Holotype*** 2.2 mm, 2.44 × as long as wide. Body brown, antennae and legs lighter. ***Head***: epistoma smooth. Frons strongly shiny, finely punctate, setose; each puncture bearing a long, erect hair-like seta. Eyes narrowly and moderately emarginate. Submentum narrow, triangular, deeply impressed. Antennal scape regularly thick, much shorter than club. Pedicel shorter than funicle. Club circular, flat, type 3; segment 1 corneous, subconvex on anterior face, occupying basal ~1/3; segment 2 narrow, subconvex, corneous; segments 1 and 2 present on posterior face. ***Pronotum***: 1.0 × as long as wide. In dorsal view basic and parallel-sided, type 2, sides parallel in basal 2/3, rounded anteriorly; anterior margin without serrations. In lateral view uniformly rounded without a clear summit, type 1. Anterior slope with densely spaced, broad fine asperities, becoming lower and more strongly transverse towards summit. Disc smooth, strongly shiny with sparse, minute punctures, some longer hair-like setae at margins. Lateral margins obliquely costate. ***Elytra***: 1.4 × as long as wide, 1.4 × as long as pronotum. Scutellum minute. Elytra round, parallel-sided in basal 69%, then broadly rounded to apex, apex entire. Disc subshiny; strial punctures large, deep, each bearing a recumbent seta the length of two punctures; interstriae flat, minutely, moderately punctate, unarmed, each puncture bearing a long semi-erect bristle-like seta. Declivity gradually rounded, occupying ~1/2 of elytra, smooth, shiny, declivital face weakly convex; striae 1 and 2 feebly impressed, strial punctures larger, deeper than those of disc, each puncture bearing a recumbent seta as long as two punctures; interstriae weakly convex, uniformly denticulate along their entire lengths, setae thick, erect, bristle-like, twice as long as interstriae 1 width. Posterolateral margin feebly carinate to striae 6, primarily visible between suture and striae 2, unequally serrate. ***Legs***: protibiae obliquely triangular, broadest at apical 1/3; apical 1/2 of outer margin with seven large, socketed denticles, their length longer than basal width. Meso- and metatibiae flattened; outer margin evenly rounded with nine and eight large, socketed denticles, respectively.

##### Etymology.

L. *murinus* = of mice. In reference to the species hairy globular appearance. Adjective.

##### Distribution.

Ecuador (Orellana).

##### Biology.

The holotype was collected by canopy fogging.

#### Coptoborus newt sp. nov.

Taxon classificationAnimaliaColeopteraCurculionidae

F2C72EF4-4E53-5D8A-AC6D-A02E0A7A168E

http://zoobank.org/917E61F0-45E1-472E-9971-DE1EDAF4AF1D

Figure 11A–C, M

##### Type material.

***Holotype*,** female, Peru: Madre de Dios Dept., Los Amigos Biological Station, CM2, GPS 12.4492°S, 70.2517°W, Smith, Hulcr, 17–18.v.2008, sample Peru 96c 9.1 cm diameter branch (MUSM). ***Paratypes***, female, as holotype (MSUC, 1; MUSM, 1; NHMUK, 1; NMNH, 1); Loreto Pr., nr. jct. Rio Maranon & Ucayali, 4.8°S, 73.5°W, 6–20-VIII-1994, P. Skelley, flight trap (FSCA, 1).

##### Diagnosis.

1.7 mm (mean = 1.7 mm; n = 4), 2.83 × as long as wide. This species is distinguished by the elytral apex attenuate and weakly emarginate, declivital interstriae 2 denticulate along entire length, denticles as numerous but smaller than those of interstriae 1, posterolateral margin of declivity costate, armed with two large denticles, and declivital slope gradual.

##### Similar species.

*C. amplissimus*, *C. catulus*, *C. incomptus*, *C. scully*.

##### Description

**(female).** 1.7 mm (mean = 1.7 mm; n = 4), 2.83 × as long as wide (***holotype*** 1.7 mm, 2.83 × as long as wide). Body light brown, elytra darker, antennae and legs lighter. ***Head***: epistoma smooth. Frons strongly shiny, finely punctate, setose; each puncture bearing a long, erect hair-like seta. Eyes narrowly and deeply emarginate. Submentum large, triangular, slightly impressed. Antennal scape short and thick, as long as club. Pedicel shorter than funicle. Club circular, flat, type 3; segment 1 corneous, transverse on anterior face, occupying basal ~1/3; segment 2 narrow, transverse, corneous; segments 1 and 2 present on posterior face. ***Pronotum***: 1.0 × as long as wide. In dorsal view basic and parallel-sided, type 2, sides parallel in basal 3/4, rounded anteriorly; anterior margin without serrations. In lateral view elongate, disc longer than anterior slope, type 7, summit prominent, on anterior 5/7. Anterior slope with densely spaced, broad fine asperities, becoming lower and more strongly transverse towards summit. Disc strongly shiny with sparse, minute punctures, some longer hair-like setae at margins. Lateral margins obliquely costate. ***Elytra***: 1.8 × as long as wide, 1.8 × as long as pronotum. Scutellum minute. Elytra attenuate, parallel-sided in basal 73%, then acutely rounded to apex, apex weakly emarginate. Disc smooth, shiny; striae minutely punctate, glabrous; interstriae flat, sparsely, minutely punctate, unarmed, each puncture bearing a long, erect seta. Declivity gradually rounded, occupying ~1/3 of elytra, smooth, shiny, declivital face convex; striae not impressed, strial punctures larger, deeper than those of disc, each puncture bearing a semi-recumbent seta as long as two punctures, striae 1 parallel to suture; interstriae flat, interstriae 1 and 3 each with 4–5 and 5–7 respectively, subequal, uniformly spaced small denticles, interstriae 2 with a row of minute denticles, denticles on interstriae 2 much smaller than those of interstriae 1 or 3, interstrial setae moderately dense thick erect bristle-like, interstriae 1 with an additional sparse row of slightly shorter setae. Posterolateral margin of declivity costate, armed with two large denticles. ***Legs***: protibiae semi-circular with evenly rounded outer margin, broadest at apical 1/3; apical 1/2 of outer margin with seven large, socketed denticles, their length longer than basal width. Meso- and metatibiae flattened; outer margin evenly rounded with seven large, socketed denticles.

##### Etymology.

Portrayed by Carrie Henn, Newt (Rebecca Jordan) is the sole survivor of the Xenomorph infestation of the colony on LV-426 in ‘Aliens’ (1986). Noun in apposition.

##### Distribution.

Peru (Loreto, Madre de Dios).

##### Biology.

The species was collected from a 9.1 cm diameter branch of an unidentified tree.

**Figure 11.**
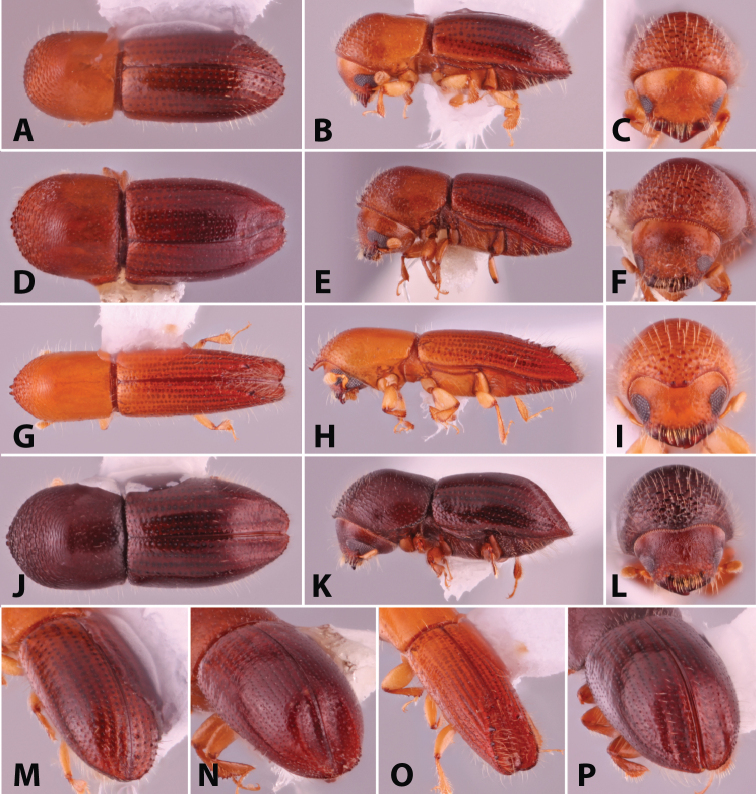
Dorsal, lateral, frontal and declivital view of *Coptoborus newt* holotype, 1.7 mm (**A–C, M**), *C. nudulus*, 2.3–2.4 mm (**D–F, N**), *C. obtusicornis*, 3.0–3.4 mm (**G–I, O**), *C. ochromactonus*, paratype, 2.5–2.6 mm (**J–L, P**). All photographs by SMS.

#### Coptoborus nudulus

Taxon classificationAnimaliaColeopteraCurculionidae

Wood, 2007

90DE82CF-A119-5B16-8F39-514B76B9388E

Figure 11D–F, N

Coptoborus nudulus Wood, 2007: 394.

##### Type material.

***Holotype*** (MEFEIS), not examined. ***Paratype*** (NMNH).

##### New records.

Ecuador: Napo Prov. [= Orellana Prov.], Res[erva]. Ethnica Waorani, 1 km S. Okone Gare Camp, Trans[ect]. Ent[omology]., 00°39'10"S, 076°26'W, 220 m, October 1995, T.L. Erwin et al. collectors, insecticidal fogging, terra firme forest, trans[ect] 2, sta[tion] 1, Erwin lot #1181 (ICB, 1; NMNH, 1); as previous except: sta[tion] 4, Erwin lot #1184 (ICB, 1; NMNH, 1); as previous except: trans[ect] 9, sta[tion] 10, Erwin lot #1260 (NMNH, 1); as previous except: January 1996, trans[ect] 2, sta[tion] 1, Erwin lot #1411 (ICB, 1; NMNH, 1); as previous except: October 1995, trans[ect] 2, sta[tion] 1, Erwin lot #1181; as previous except: October 1996, Erwin lot #1671 (ICB, 1; NMNH, 1); Tiputini Biodiversity Station, 00°37'55"S, 076°08'39"W, 220–250 m, June 1998, T.L. Erwin et al. collectors, insecticidal fogging, terra firme forest, trans[ect] 6, sta[tion] 2, Erwin lot #1851 (NMNH, 1).

##### Diagnosis.

2.3–2.4 mm (mean = 2.34 mm; n = 5), 2.56–2.67 × as long as wide. This species is distinguished by the elytral apex attenuate and weakly emarginate, declivity strongly sulcate along interstriae 2, declivital interstriae 1–3 unarmed, declivity glabrous, declivital interstriae 3 clearly elevated and costate, and declivity smooth, shiny.

##### Similar species.

*C. busoror*, *C. leeloo*, *C. ochromactonus*, *C. pilisoror*, *C. ripley*, *C. sororcula*, *C. spicatus*.

##### Distribution.

Brazil (Mato Grosso), Ecuador* (Orellana), Peru (Loreto).

##### Biology.

Specimens were collected by canopy fogging.

##### Remarks.

Wood (2007) incorrectly reported the holotype’s location as MZUSP. The holotype is in MEFEIS.

#### Coptoborus obtusicornis

Taxon classificationAnimaliaColeopteraCurculionidae

(Schedl, 1976) comb. nov.

7AB15033-32C3-5F75-BFC6-E841232CCD5B

Figure 11G–I, O

Sampsonius obtusicornis Schedl, 1976: 78.

##### Type material.

***Holotype*** (NHMW), examined.

##### New records.

Ecuador: Fco. Orellana P.N. Yasuní, 00°40'32"S, 76°21'19"W, 250 m, 19 Feb 2005, I. Rodríguez (PUCE, 1).

##### Diagnosis.

3.0–3.4 mm (mean = 3.22 mm; n = 5), 3.78–4.29 × as long as wide. This species is distinguished by the elytra attenuate, apex emarginate, elytra deeply excavated between interstriae 3, excavated area bearing granules or small denticles and anterior margin of pronotum with a pair of projecting serrations.

##### Similar species.

*C. vespatorius*.

##### Distribution.

Brazil (Maranhão, Mato Grosso, Paraná, São Paulo), Costa Rica (Heredia), Ecuador* (Orellana), Peru (Huánuco, Junín, Loreto, Madre de Dios).

##### Biology.

This species has only been collected from *Cecropia* (Urticaceae) (Smith et al. 2017).

##### Remarks.

This species shares remarkable morphological convergence with *Sampsonius* Eggers, 1935, especially with regard to the prontotum shape in dorsal view, (type b) conspicuously long and acuminate frontally. This pronotal shape has been assumed to be diagnositic for *Sampsonius* but with the exception of the pronotal form, *S. obtusicornis* and *Sampsonius* are quite different and this shape is due to convergence. We transfer this species to *Coptoborus* due to the following combination of characters (*Sampsonius* characters given first): protibia slender with greatly enlarged and prominent apical mucro, and outer margin and posterior face granulate vs. protibia distinctly triangular, apical mucro small, and posterior face unarmed; strongly concave lateral edge of pronotum vs. convex lateral margin; antennal club type 4 with sutures 1 and 2 strongly procurved vs. antennal club type 3 with sutures 1 and 2 transverse (Bright 1991; Petrov and Mandelshtam 2009; Smith 2017).

#### Coptoborus ochromactonus

Taxon classificationAnimaliaColeopteraCurculionidae

Smith & Cognato, 2014

E983825F-27E5-55C1-BD6F-3E1334B59AAC

Figure 11J–L, P

Coptoborus ochromactonus Smith & Cognato, 2014 (in Stilwell et al. 2014): 677.

##### Type material.

***Holotype*** (PUCE), ***paratypes*** (MSUC, 18), examined.

##### New records.

Ecuador: Cotopaxi Prov., Otonga, 79°0.197'W, 0°25.158'S, 1970 m, Á. Barragán (PUCE, 5). Guayas Prov., El Empalme, m5 14, 21.ii.2013, Y. Castro, ex cultivated balsa (MSUC, 1).

##### Diagnosis.

2.5–2.6 mm (mean = 2.56 mm; n = 5), 2.48–2.63 × as long as wide. This species is distinguished by the elytral apex attenuate and weakly emarginate, declivity distinctly sulcate along interstriae 2, declivital interstriae 2 unarmed, interstriae 1 and 3 armed, declivital striae 1 and 2 parallel on declivital face and widely spaced, and declivital striae 2 punctate. It is most similar to *C. busoror* and can be further distinguished by the smaller size 2.5–2.6 mm vs. 2.7 mm, and stouter body, 2.5–2.6 × as long as wide vs. 2.7 × as long as wide, stouter pronotum, 1.05–1.1 ×, vs. 1.2 × as long as wide, and distribution west of the Andes vs. east of the Andes.

##### Similar species.

*C. busoror*, *C. leeloo*, *C. nudulus*, *C. pilisoror*, *C. ripley*, *C. sororcula*, *C. spicatus*.

##### Distribution.

Ecuador (Cotopaxi, Guayas, Los Ríos, Santo Domingo de los Tsáchilas).

##### Biology.

This species has only been collected from balsa, *Ochroma pyrimidale* (Malvaceae), and is a serious pest of balsa in Ecuador. The biology of *C. ochromactonus* has been studied in detail (Stilwell et al. 2014; Castro et al. 2019; Martínez et al. 2020).

#### Coptoborus osbornae sp. nov.

Taxon classificationAnimaliaColeopteraCurculionidae

6EC3FA31-6808-5A25-A6FB-7F573A59D28B

http://zoobank.org/1003A9CA-76AA-4B29-9A4A-12D4BF59E4E5

Figure 12A–C, M

##### Type material.

***Holotype*,** female, Ecuador: Napo Prov. [= Orellana Prov.], Res[erva]. Ethnica Waorani, 1 km S. Okone Gare Camp, Trans[ect]. Ent[omology]., 00°39’10”S, 076°26’W, 220 m, October 1994, T.L. Erwin et al. collectors, insecticidal fogging, terra firme forest, trans[ect] 9, sta[tion] 3, Erwin lot #872 (ICB). ***Paratypes***, female, as holotype except: January 1996, trans[ect] 1, sta[tion] 1, Erwin lot #1401 (NMNH, 1); as holotype except: Tiputini Biodiversity Station, 00°37'55"S, 076°08'39"W, 220–250 m, February 1999, T.L. Erwin et al. collectors, insecticidal fogging, terra firme forest, trans[ect] 4, sta[tion] 4, Erwin lot #2038 (ICB, 1); as holotype except: Yasuni National Park, Estacíon Científica Yasuní, 00°39.675'S, 76°24.023'W, 11.ii.2018, R. Osborn, EC18-41, ex 6 cm dia. branch (MSUC, 3; PUCE, 1).

##### Diagnosis.

1.5–1.7 mm (mean = 1.6 mm; n = 5), 2.67–3.0 × as long as wide. This species is distinguished by the elytral apex broadly rounded and entire, posterolateral margin feebly carinate to striae 6, primarily visible between suture and striae 2, unequally serrate and appearing broken, declivity very steep, occupying poster quarter of elytra, and declivital interstrial setae as long as interstriae 1 width.

##### Similar species.

*C. murinus*.

##### Description

**(female).** 1.5–1.7 mm (mean = 1.6 mm; n = 5), 2.67–3.0 × as long as wide (***holotype*** 1.6 mm, 2.83 × as long as wide). Body brown, antennae and legs lighter. ***Head***: epistoma tuberculate. Frons subshiny, finely punctate, setose; each puncture bearing a long, erect hair-like seta. Eyes narrowly and deeply emarginate. Submentum large, triangular, deeply impressed. Antennal scape short and thick, much shorter than club. Pedicel shorter than funicle. Club circular, flat, type 3; segment 1 corneous, transverse on anterior face, occupying basal ~1/3; segment 2 narrow, transverse, corneous; segments 1 and 2 present on posterior face. ***Pronotum***: 1.0–1.2 × as long as wide. In dorsal view basic and parallel-sided, type 2, sides parallel in basal 3/5, rounded anteriorly, abundantly covered with long hair-like setae; anterior margin without serrations. In lateral view uniformly rounded without a clear summit, type 1. Anterior slope with densely spaced, broad fine asperities, becoming lower and more strongly transverse towards summit. Disc strongly shiny with sparse, minute punctures. Lateral margins obliquely costate. ***Elytra***: 1.6–1.7 × as long as wide, 1.7 × as long as pronotum. Scutellum small. Elytra round, parallel-sided in basal 80–88%, then broadly rounded to apex, apex entire. Disc shagreened, subshiny; strial punctures large, shallow, each bearing a recumbent seta the length of a puncture; interstriae flat, minutely, moderately punctate, unarmed, each puncture bearing a long semi-erect bristle-like seta. Declivity very steep, occupying ~1/4 of elytra, smooth, subshiny, declivital face weakly convex; striae 1 and 2 feebly impressed, strial punctures larger, deeper than those of disc, each puncture bearing a recumbent seta as long as 1.5 punctures; interstriae weakly convex, uniformly minutely granulate along their entire lengths, setae thick, erect, bristle-like, as long as interstriae 1 width. Posterolateral margin feebly carinate to striae 6, primarily visible between suture and striae 2, unequally serrate. ***Legs***: protibiae obliquely triangular, broadest at apical 1/4; apical 1/2 of outer margin with six large, socketed denticles, their length longer than basal width. Meso- and metatibiae flattened; outer margin evenly rounded with seven large, socketed denticles.

##### Etymology.

For Rachel Osborn, Ph.D. student of AIC, who was the first to culture the symbiotic fungi of this species. Noun in genitive.

##### Distribution.

Ecuador (Orellana).

##### Biology.

Specimens were collected by canopy fogging and from an unidentified 6 cm diameter branch.

**Figure 12.**
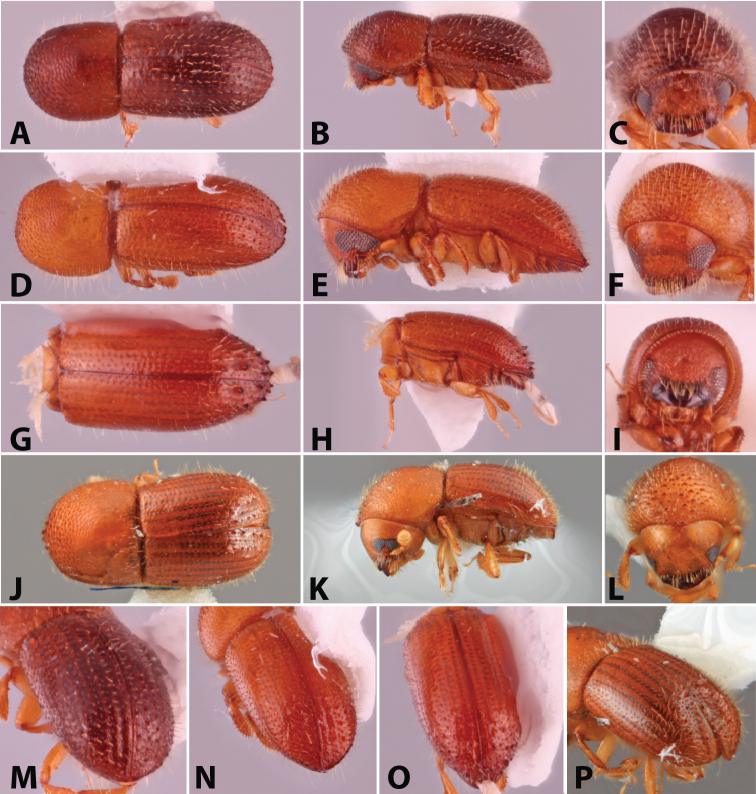
Dorsal, lateral, frontal and declivital view of *Coptoborus osbornae* holotype, 1.5–1.7 mm (**A–C, M**), *C. panosus* holotype, 2.4 mm (**D–F, N**), *C. papillicauda* holotype, 2.0 mm (**G–I, O**), *C. paurus* holotype, 1.7 mm (**J–L, P**). All photographs by SMS, except **J–L, P** copyright National Museum of Natural History, Smithsonian Institution, Washington, D.C., published by permission.

#### Coptoborus panosus sp. nov.

Taxon classificationAnimaliaColeopteraCurculionidae

68402C3B-E9FA-53E8-B6FB-E65B698BA5BC

http://zoobank.org/82A6E5D9-4386-4FB7-8A00-9E6EE6D47D65

Figure 12D–F, N

##### Type material.

***Holotype*,** female, French Guiana: Amazone Nature Lodge, 30 km SE Roura on Kaw Rd., 10–18-IV-2007. D.G. & J.E. Eger, 4°33.570'N, 052°12.433'W 300 m, MV light trap (NMNH).

##### Diagnosis.

2.4 mm (n = 1), 2.67 × as long as wide. This species is distinguished by the elytra attenuate, declivital interstriae 2 convex and granulate, posterolateral margin of elytra carinate from apex to interstriae 7, and declivital interstriae moderately covered with long, erect hair-like setae.

##### Similar species.

*C. vrataski*.

##### Description

**(female). *Holotype*** 2.4 mm, 2.67 × as long as wide. Body light brown, antennae and legs lighter. ***Head***: epistoma tuberculate. Frons dull, tuberculate, finely punctate, setose; each puncture bearing a long, erect hair-like seta. Eyes broadly and moderately emarginate. Submentum large, triangular, deeply impressed. Antennal scape regularly thick, much shorter than club. Pedicel shorter than funicle. Club longer than wide, flat, type 4; segment 1 corneous, procurved on anterior face, occupying basal ~1/3; segment 2 narrow, weakly procurved, corneous; segments 1 and 2 present on posterior face. ***Pronotum***: 0.9 × as long as wide. In dorsal view basic and parallel-sided, type 2, sides parallel in basal 3/4, rounded anteriorly, abundantly covered with long hair-like setae; anterior margin with four subequal serrations. In lateral view elongate, disc longer than anterior slope, type 7, summit prominent, on anterior 2/3. Anterior slope with densely spaced, broad fine asperities, becoming lower and more strongly transverse towards summit. Disc dull with sparse, minute punctures. Lateral margins obliquely costate. ***Elytra***: 1.7 × as long as wide, 1.9 × as long as pronotum. Scutellum minute. Elytra attenuate, parallel-sided in basal 2/3, then acutely rounded to apex, apex entire. Disc smooth, shiny; strial punctures moderate, deep, each bearing a recumbent seta the length of a puncture; interstriae flat, densely, minutely punctate, unarmed, each puncture bearing a long, erect hair-like seta. Declivity steep, occupying ~1/3 of elytra, smooth, shiny, declivital face weakly convex; striae not impressed, strial punctures larger, deeper than those of disc, each puncture bearing a semi-erect seta as long as two punctures; interstriae weakly convex, sparsely granulate, granules small, separated by the distance of three granules, interstriae moderately setose, setae long, erect hair-like, slightly longer than interstriae 1 width. Posterolateral margin apically produced, sharply carinate and serrate. ***Legs***: protibiae obliquely triangular, broadest at apical 1/5; apical 1/2 of outer margin with eight large, socketed denticles, their length longer than basal width. Meso- and metatibiae flattened; outer margin evenly rounded with nine large, socketed denticles.

##### Etymology.

L. *panosus* = like bread. In reference to the species shape and color which resemble a baguette. Adjective.

##### Distribution.

French Guiana.

##### Biology.

Unknown.

#### Coptoborus papillicauda sp. nov.

Taxon classificationAnimaliaColeopteraCurculionidae

5851C701-C979-5055-B1FD-3F7B7CB59591

http://zoobank.org/1944F4C9-A171-4000-95FD-B88CDE973A82

Figure 12G–I, O

##### Type material.

***Holotype*,** female, Suriname: Sipaliwini, 2.977312°N, 55.38500°W, 200 m, Camp 4 (low), Kasikasima, T. Larsen, 20–25.iii.2012, FIT, SR12-0320-TN1, 2012 CI-RAP survey (NZCS).

##### Diagnosis.

2.0 mm (n = 1), 3.33 × as long as wide. This species is distinguished by the elytral apex attenuate and entire, declivital interstriae 2 convex, declivital interstriae 1 and 3 denticulate and interstriae 2 unarmed, declivital subapical margin armed with three denticles, and declivital interstriae 1 denticles large, 1–2 × high as wide.

##### Similar species.

*C. chica*.

##### Description

**(female). *Holotype*** 2.0 mm, 3.33 × as long as wide. Body light brown, elytra darker, antennae and legs lighter. ***Head***: epistoma smooth. Frons subshiny, finely punctate, setose; each puncture bearing a long, erect hair-like seta. Eyes broadly and moderately emarginate. Submentum large, triangular, deeply impressed. Antennal scape short and thick, much shorter than club. Pedicel shorter than funicle. Club circular, flat, type 3; segment 1 corneous, weakly convex on anterior face, occupying basal ~2/5; segment 2 narrow, transverse corneous; segments 1 and 2 present on posterior face. ***Pronotum***: 1.3 × as long as wide. In dorsal view long and rounded frontally, type 7, sides parallel in basal 4/5, rounded anteriorly; anterior margin without serrations. In lateral view elongate, disc longer than anterior slope, type 7, summit prominent, on anterior 3/4. Anterior slope with densely spaced, broad fine asperities, becoming lower and more strongly transverse towards summit. Disc strongly shiny with sparse, minute punctures, some longer hair-like setae at margins. Lateral margins obliquely costate. ***Elytra***: 2.0 × as long as wide, 1.5 × as long as pronotum. Scutellum minute. Elytra attenuate, parallel-sided in basal 3/4, then acutely rounded to apex, apex entire. Disc smooth, shiny; striae minutely punctate, glabrous; interstriae flat, sparsely, minutely punctate, unarmed, each puncture bearing a long, erect seta. Declivity gradually rounded, occupying ~1/3 of elytra, smooth, shiny, declivital face weakly convex; striae very shallowly impressed, strial punctures larger, deeper than those of disc, glabrous; striae 1 irregular, slightly laterally broadened from base to declivital midpoint and then narrowing towards apex; interstriae flat, interstriae 1 and 3 each with three large denticles, interstriae 2 unarmed, those of interstriae 1 and 3 subequal, 1–2 × high as wide, interstriae with a sparse row of erect bristle-like setae. Posterolateral margin with interstriae 3 and 9 joining, forming a feebly carina armed with three large, acute denticles and continuing submarginally to apex. ***Legs***: protibiae obliquely triangular, broadest at apical 1/5; apical 1/2 of outer margin with six large, socketed denticles, their length longer than basal width. Meso- and metatibiae flattened; outer margin evenly rounded with eight and seven large, socketed denticles, respectively.

##### Etymology.

L. *papilla* = rounded protuberance of the body, *cauda* = tail. In reference to the appearance of papillae (granules) on the declivity. Noun in apposition.

##### Distribution.

Suriname.

##### Biology.

Unknown.

#### Coptoborus paurus

Taxon classificationAnimaliaColeopteraCurculionidae

(Wood, 2007) comb. nov.

AE2F190B-A616-5725-8C5F-B438E02130C4

Figure 12J–L, P

Theoborus paurus Wood, 2007: 388.

##### Type material.

***Holotype*** (NMNH), examined.

##### New records.

None.

##### Diagnosis.

1.7 mm, 2.1 × as long as wide (Wood 2007). This species is distinguished by the broadly rounded and entire elytral apex, posterolateral margin rounded, anterior margin of pronotum with two projecting serrations, declivital interstrial setae much longer than the combined width of striae 1 and interstriae 1, declivital interstriae 2 sulcate, and interstriae 1 granulate, interstriae 3 denticulate (those larger than interstriae 1 granules), interstriae 2 with a staggered row of minute obscure granules.

##### Similar species.

*C. doliolum*, *C. erwini*.

##### Distribution.

Costa Rica (Heredia).

##### Biology.

This species has only been collected from *Protium panamensis* (Burseraceae) (Wood 2007).

#### Coptoborus pilisoror sp. nov.

Taxon classificationAnimaliaColeopteraCurculionidae

34D4887F-F06F-5B20-8FD7-BB0B38A0E26A

http://zoobank.org/0C40DAB4-479A-4E84-B2A5-44A23F626A20

Figure 13A–C, M

##### Type material.

***Holotype*,** female, Ecuador: Napo Prov. [= Orellana Prov.], Tiputini Biodiversity Station, 00°37'55"S, 076°08'39"W, 220–250 m, February 1999, T.L. Erwin et al. collectors, insecticidal fogging, terra firme forest, trans[ect] 6, sta[tion] 4, Erwin lot #2053 (ICB). ***Paratypes***, female: as holotype (NMNH, 1); as holotype except: sta[tion] 2, Erwin lot #2051 (ICB, 1).

##### Diagnosis.

1.8 mm (mean = 1.8 mm; n = 3), 2.57 × as long as wide. This species is distinguished by the elytral apex attenuate and weakly emarginate, declivity moderately sulcate along interstriae 2, declivital interstriae 1–3 unarmed, and declivity densely covered in thick recumbent setae.

##### Similar species.

*C. busoror*, *C. leeloo*, *C. nudulus*, *C. ochromactonus*, *C. ripley*, *C. sororcula*, *C. spicatus*.

##### Description

**(female). *Holotype*** 1.8 mm, 2.57 × as long as wide. Body brown, antennae and legs lighter. ***Head***: epistoma smooth. Frons shiny, finely punctate, setose; each puncture bearing a long, erect hair-like seta. Eyes broadly and moderately emarginate. Submentum large, triangular, deeply impressed. Antennal scape short and thick, much shorter than club. Pedicel shorter than funicle. Club longer than wide, flat, type 3; segment 1 corneous, transverse on anterior face, occupying basal ~1/3; segment 2 narrow, transverse, corneous; segments 1 and 2 present on posterior face. ***Pronotum***: 1.0 × as long as wide. In dorsal view basic and parallel-sided, type 2, sides parallel in basal 3/5, rounded anteriorly; anterior margin with six serrations, median pair largest. In lateral view uniformly rounded without a clear summit, type 1. Anterior slope with densely spaced, broad coarse asperities, becoming lower and more strongly transverse towards summit. Disc reticulate, dull with sparse, minute punctures, some longer hair-like setae at margins. Lateral margins obliquely costate. ***Elytra***: 1.6 × as long as wide, 1.6 × as long as pronotum. Scutellum minute. Elytra attenuate, parallel-sided in basal 73%, then acutely tapered to apex, apex weakly emarginate. Disc smooth, subshiny; strial punctures moderate, shallow, glabrous; interstriae flat, sparsely, minutely punctate, unarmed, glabrous. Declivity gradual, strongly shagreened, dull, appearing bisulcate, occupying apical 2/5 of elytra; striae not impressed, striae 1 and 2 parallel, strial punctures deeper than those of disc, each puncture bearing a recumbent seta the length of two punctures; interstriae punctate, interstriae covered in 3–5 rows of confused recumbent stout setae, interstriae 2 feebly sulcate, unarmed; interstriae 1 and 3 feebly costate unarmed. Posterolateral margin with interstriae 3 and 9 joining, forming a costa and continuing submarginally to apex. ***Legs***: protibiae obliquely triangular, broadest at apical 1/4; apical 1/2 of outer margin with six large, socketed denticles, their length longer than basal width. Meso- and metatibiae flattened; outer margin evenly rounded with seven and five large, socketed denticles, respectively.

##### Etymology.

L. *pilosus* = hairy, *soror* = sister. In reference to the dense short setae of the declivity. Noun in apposition.

##### Distribution.

Ecuador (Orellana).

##### Biology.

Specimens were collected by canopy fogging.

**Figure 13.**
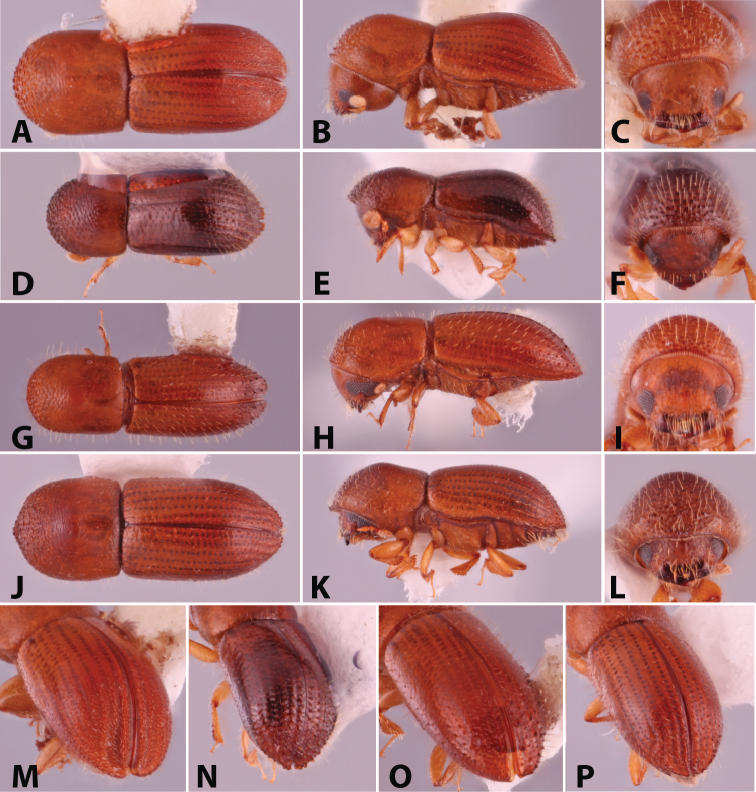
Dorsal, lateral, frontal and declivital view of *Coptoborus pilisoror* holotype, 1.8 mm (**A–C, M**), *C. pristis*, 1.6–2.1 mm (**D–F, N**), *C. pseudotenuis*, 1.9–2.1 mm (**G–I, O**), *C. puertoricensis*, 2.2–2.4 mm (**J–L, P**). All photographs by SMS.

#### Coptoborus pristis

Taxon classificationAnimaliaColeopteraCurculionidae

(Wood, 1974) comb. nov.

2899D614-0A6E-5AD5-B692-30EDA896D363

Figure 13D–F, N

Xyleborus pristis Wood, 1974: 32.
Theoborus pristis (Wood): Wood 1982: 773.

##### Type material.

***Holotype*** (NMNH), ***paratypes*** (NMNH, 2), examined.

##### New records.

Brazil: Bahia, Camacan, Serra Bonita Reserve, 15°23.429'S, 39°33.810'W, 700–100 m, 6–14.V.2013, AI Cognato, SM Smith, CAH Flechtmann, Brazil 2013-11b, ex fallen *Tibouchina* branch 1 cm diameter (MSUC, 4); as previous except: ex 25 cm dia. *Senna* bole (MSUC, 1). Ecuador: El Cotopaxi, La Mana, Yakusinchi Nature Reserve, 00°57.030'S, 79°08.717'W, 450–550 m, 12–14.v.2015, Cognato, Smith, Osborn, Martinez et al., sample EC 5, ex 3 cm dia. branch (PUCE, 1); as previous except: sample EC 6, ex 2 cm dia. branch with thin bark (MSUC, 1; PUCE, 1); as previous except: EC 21, ex small 7 mm dia. twigs (MSUC, 2); as previous except: EC 19, ex bole of small tree, 3 cm dia. (MSUC, 1). Los Ríos, Canton Valencia, Murucunba Nature Reserve, 00°38.544'S, 79°08.902'W, 731 m, 16.v.2015, Cognato, Smith, Osborn, Martinez et al., sample EC 47A, ex twigs and branches of ‘Canelo’ (MSUC, 1). Napo Prov. [= Orellana Prov.], Res[erva]. Ethnica Waorani, 1 km S. Okone Gare Camp, Trans[ect]. Ent[omology]., 00°39'10"S, 076°26'W, 220 m, July 1996, T.L. Erwin et al. collectors, insecticidal fogging, terra firme forest, trans[ect] 2, sta[tion] 9, Erwin lot #1539 (ICB, 1).

##### Diagnosis.

1.6–2.1 mm (mean = 1.78 mm; n = 5), 2.67–3.0 × as long as wide. This species is distinguished by the elytral apex broadly rounded and entire, posterolateral margin distinctly carinate to striae 6, carina unequally serrate and appearing broken, serrations on interstriae 1 and 2 subquadrate that are at least 2 × the size of other serrations.

##### Similar species.

*C. micarius*.

##### Distribution.

Brazil* (Bahia), Costa Rica (Cartago, Puntarenas), Ecuador* (El Cotopaxi, Orellana), Guatemala* (Zacapa), Panama (Panamá Oeste), Peru (Madre de Dios).

##### Biology.

This species is only known from *Senna* (Fabaceae) and *Miconia caudata* and *Tibouchina* (Melastomataceae). Wood (1982) reported that the tunnels were constructed in limbs and saplings of unidentified trees 10–15 cm in diameter. In addition, we report this species from twigs as small as 7 mm in diameter and a bole 25 cm in diameter. Specimens were also collected by canopy fogging.

#### Coptoborus pseudotenuis

Taxon classificationAnimaliaColeopteraCurculionidae

(Schedl, 1936) comb. nov.

AF17E316-1A35-593E-B914-E8C3654E2638

Figure 13G–I, O

Xyleborus pseudotenuis Schedl, 1936: 109.
Coptoborus pseudotenuis (Schedl): Wood 1982: 802.
Xyleborus pseudotenuis Schedl: Bright 2019: 297.
Xyleborus tenuis Schedl, 1948: 269: Synonymy: Wood 1976: 349.

##### Type material.

***Holotype****Xyleborus pseudotenuis* (NHMW), examined. ***Holotype****Xyleborus tenuis* (NHMW), examined.

##### New records.

Ecuador: Napo Prov. [= Orellana Prov.], Res[erva]. Ethnica Waorani, 1 km S. Okone Gare Camp, Trans[ect]. Ent[omology]., 00°39'10"S, 076°26'W, 220 m, July 1995, T.L. Erwin et al. collectors, insecticidal fogging, terra firme forest, trans[ect] 10, sta[tion] 10, Erwin lot #1130 (NMNH, 1); as previous except: trans[ect] 4, sta[tion] 3, Erwin lot #1093 (ICB, 1); as previous except: October 1995, trans[ect] 4, sta[tion] 4, Erwin lot #1204 (NMNH, 1); as previous except: trans[ect] 5, sta[tion] 6, Erwin lot #1216, (ICB, 1); as previous except: trans[ect] 8, sta[tion] 6, Erwin lot #1245 (ICB, 1); except: July.1996, trans[ect] 4, sta[tion] 3, Erwin lot #1553 (NMNH, 1); as previous except: Tiputini Biodiversity Station, 00°37'55"S, 076°08'39"W, 220–250 m, June. 1998, T.L. Erwin et al. collectors, insecticidal fogging, terra firme forest, trans[ect] 8, sta[tion] 9, Erwin lot #1878 (NMNH, 1); as previous except: October 1998, trans[ect] 9, sta[tion] 5, Erwin lot #1984 (ICB, 1). Peru: Madre de Dios Dept., Los Amigos Biological Station, 12°34.9S, 70°6.04W, Smith, Hulcr, 26.iv.–27.v.2008, sample Peru 14, *Cecropia* sp. trunk (MSUC, 4; MUSM, 2).

##### Diagnosis.

1.9–2.1 mm (mean = 2.0 mm; n = 5), 3.0–3.33 × as long as wide. This species is distinguished by the elytral apex attenuate and weakly emarginate, declivital interstriae 2 denticulate along the entire length, denticles on declivital interstriae 1 and 3 subequal, posterolateral margin of declivity with interstriae 3 and 9 joining, forming a carina and continuing submarginally to apex, declivital interstriae distinctly impressed, and anterior margin of pronotum without a row of serrations.

##### Similar species.

*C. artetenuis*, *C. exilis*.

##### Distribution.

Colombia (Valle de Cauca), Costa Rica (Limón), Dominica, Ecuador* (Orellana), Grenada, Guadeloupe, Martinique, Mexico (Campeche, San Luis Potosí, Tabasco, Veracruz), Panama (Panamá), Peru (Loreto, Madre de Dios), Saint Lucia, Saint Vincent and the Grenadines, Trinidad, United States (Florida), Venezuela (Aragua, Mérida).

##### Biology.

This species is polyphagous and has been collected from *Cordia* (Cordiaceae), *Hevea brasiliensis* (Euphorbiaceae), *Acacia* (Fabaceae), *Heliocarpus appendiculatus*, *Theobroma cacao* (Malvaceae), *Coffea* (Rubiaceae), *Cestrum* (Solanaceae), *Cecropia* (Urticaceae) (Wood and Bright 1992; Bright and Skidmore 1997; Wood 2007). Wood (2007) reported collecting the species from limbs and a log 5–20 cm in diameter. Specimens were also collected by canopy fogging.

##### Remarks.

Bright (2019) placed this species back in *Xyleborus* without discussion. This species clearly beongs in *Coptoborus* and shares the characters outlined in the generic diagnosis and has been shown to be correctly placed in *Coptoborus*, rather than *Xyleborus* in several molecular phylogenetic studies (e.g. Cognato et al. 2011; Gohli et al. 2017; Cognato et al. 2018).

#### Coptoborus puertoricensis

Taxon classificationAnimaliaColeopteraCurculionidae

(Bright & Torres, 2006) comb. nov.

F6DD7577-E4DB-5888-B2E3-A760367B2395

Figure 13J–L, P

Theoborus puertoricensis Bright & Torres, 2006: 414.
Coptoborus puertoricensis Bright, 2005 [sic]: Wood 2007: 398.
Theoborus puertoricensis Bright & Torres: Bright 2019: 275.

##### Type material.

***Holotype*** (CNCI), not examined.

##### New records.

None.

##### Diagnosis.

2.2–2.4 mm, 2.6–2.7 × as long as wide (Bright and Torres 2006). This species is distinguished by the elytral apex attenuate and weakly emarginate, declivital interstriae 2 convex, declivital interstriae 1–3 denticulate, denticles on interstriae 2 minute, distinctly smaller than those of interstriae 1 or 3, posterolateral margin of declivity with interstriae 3 and 9 joining, forming a carina and continuing submarginally to apex and declivity shagreened and dull.

##### Similar species.

*C. solitariformis*.

##### Distribution.

Dominican Republic, Puerto Rico.

##### Biology.

This species has been extensively collected via trapping and has been recorded from petioles of *Cecropia schreberiana* (Urticaceae) (Bright 2019).

##### Remarks.

The treatment of this species was based on the examination of a specimen authoratively identified by Bright from San Germán, Puerto Rico (MSUC) (see Bright 2019).

#### Coptoborus ricini

Taxon classificationAnimaliaColeopteraCurculionidae

(Eggers, 1932) comb. nov.

691E7D78-D952-5AC3-AEB7-C196A5871298

Figure 14A–C, M

Xyleborus ricini Eggers, 1932: 298.
Theoborus ricini (Eggers): Wood and Bright 1992: 661.
Xyleborus solitariceps Schedl, 1954: 45. Synonymy: Wood 1989: 176.

##### Type material.

***Holotype****Xyleborus ricini* (NMNH), examined. ***Holotype****Xyleborus solitariceps* (NHMW), examined.

##### New records.

Bolivia: Santa Cruz Dist., Potrerillos del Guenda, Perserva Natural, 17°40'S, 63°27'W, 370 m 17–22-OCT-2007, J & F Romero, ex: MV/BL (CSCA, 1); as previous except: Cline & Wappes (CSCA, 1). Peru: Madre de Dios Dept., Los Amigos Biological Station, 12°34.9S, 70°6.04W, Smith, Hulcr, 26.iv.–2.v.2008, sample Peru 40b, 4 cm diameter twig (MSUC, 7); as previous except: sample Peru 40a, 1 cm diameter twig (MSUC, 3).

##### Diagnosis.

2.3–2.5 mm (mean = 2.4 mm; n = 5), 2.3–2.67 × as long as wide. This species is distinguished by the elytral apex broadly rounded and entire, posterolateral margin continuously and smoothly carinate to striae 6 and not extended posteriad, and declivity broadly and shallowly impressed between interstriae 3.

##### Similar species.

*C. coartatus*.

##### Distribution.

Bolivia* (Santa Cruz), Brazil (Bahia, Paraná), Colombia (Cauca, Santander, Tolima), Costa Rica (Limón, Puntarenas), Dominican Republic, Honduras (Francisco Morazán), Jamaica, Mexico (Campeche, Tabasco, Veracruz), Peru* (Madre de Dios), Puerto Rico, United States (Florida), Venezuela (Miranda). Introduced to Africa (Cameroon, Côte d’Ivoire, Democratic Republic of the Congo, Equatorial Guinea, Ghana, São Tomé and Príncipe, Uganda, Zaire).

##### Biology.

This species has been recorded from many hosts in the Neotropics including: *Terminalia* sp. (Combretaceae), *Hevea brasiliensis*, *Ricinus communis* (Euphorbiaceae), *Albizia gummifera*, *Dioclea megacarpa*, *Tetrapleura tetrapetra* (Fabaceae), *Theobroma cacao* (Malvaceae), *Swietenia* sp. (Meliaceae), *Maesa rufescens* (Primulaceae), *Citrus aurantifolia* (Rutaceae) (Wood and Bright 1992). African hosts are listed in Schedl (1963: 289). Wood reported collecting the species from dying branches 3–7 cm in diameter as well as large boring in large limbs and stumps (Wood 1982, 2007) but it has also been collected in twigs as small as 1 cm in diameter.

**Figure 14.**
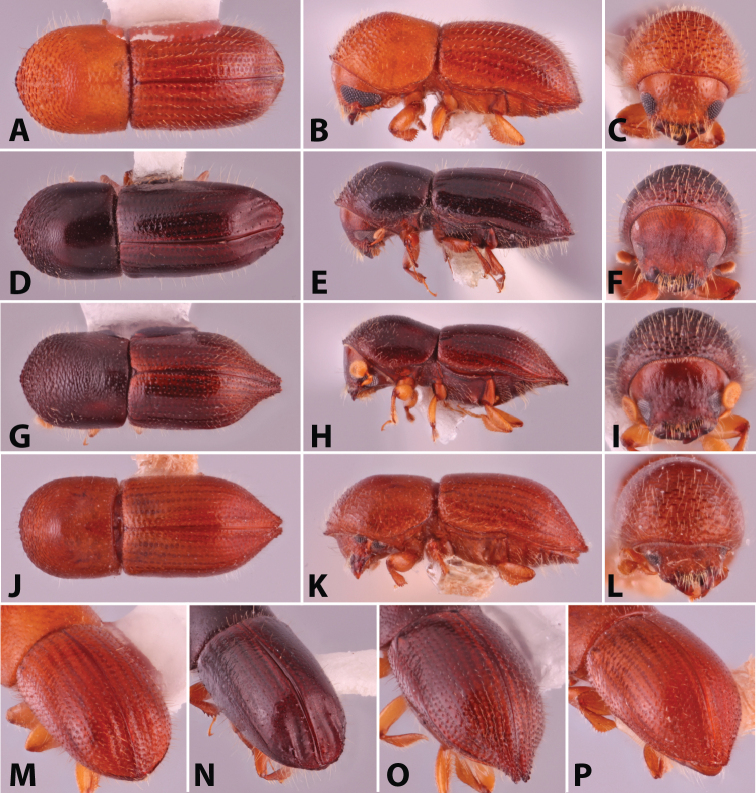
Dorsal, lateral, frontal and declivital view of *Coptoborus ricini*, 2.3–2.5 mm (**A–C, M**), *C. ripley* holotype, 3.5 mm (**D–F, N**), *C. sagitticauda* holotype, 2.3 mm (**G–I, O**), *C. sarahconnor* holotype, 2.3 mm (**J–L, P**). All photographs by SMS.

#### Coptoborus ripley sp. nov.

Taxon classificationAnimaliaColeopteraCurculionidae

98A64369-4F81-534B-B534-9E14AD4A1844

http://zoobank.org/C18AB0BB-FAED-4C67-A33D-A469E045C2DF

Figure 14D–F, N

##### Type material.

***Holotype*,** female, Ecuador: Napo Prov. [= Orellana Prov.], Res[erva]. Ethnica Waorani, 1 km S. Okone Gare Camp, Trans[ect]. Ent[omology]., 00°39'10"S, 076°26'W, 220 m, July 1996, T.L. Erwin et al. collectors, insecticidal fogging, terra firme forest, trans[ect] 3, sta[tion] 6, Erwin lot #1546 (ICB).

##### Diagnosis.

3.5 mm (n = 1), 2.69 × as long as wide. This species is distinguished by the elytral apex attenuate and weakly emarginate, declivity distinctly sulcate along interstriae 2, declivital interstriae 2 unarmed and impunctate, interstriae 1 and 3 armed, declivital striae 1 and 2 parallel on declivital face and widely spaced, and large size.

##### Similar species.

*C. busoror*, *C. leeloo*, *C. nudulus*, *C. ochromactonus*, *C. pilisoror*, *C. sororcula*, *C. spicatus*.

##### Description

**(female). *Holotype*** 3.5 mm, 2.69 × as long as wide. Body dark brown, antennae and legs lighter. ***Head***: epistoma smooth. Frons subshiny, finely punctate, setose; each puncture bearing a long, erect hair-like seta. Eyes broadly and moderately emarginate. Submentum large, triangular, slightly impressed. Antennal scape regularly thick, as long as club. Pedicel shorter than funicle. Club circular, flat, type 3; segment 1 corneous, transverse on anterior face, occupying basal ~1/3; segment 2 narrow, transverse, corneous; segments 1 and 2 present on posterior face. ***Pronotum***: 1.2 × as long as wide. In dorsal view long and rounded frontally, type 7, sides parallel in basal 3/5, rounded anteriorly; anterior margin with two projecting serrations. In lateral view elongate, disc longer than anterior slope, type 7, summit prominent, on anterior 3/5. Anterior slope with densely spaced, broad fine asperities, becoming lower and more strongly transverse towards summit. Disc reticulate, dull with sparse, minute punctures, some longer hair-like setae at margins. Lateral margins obliquely costate. ***Elytra***: 1.5 × as long as wide, 1.3 × as long as pronotum. Scutellum minute. Elytra attenuate, parallel-sided in basal 70%, then acutely tapered to apex, apex strongly emarginate. Disc smooth, shiny; strial punctures moderate, shallow, glabrous; interstriae flat, sparsely, minutely punctate, unarmed, each puncture bearing a long semi-erect hair-like seta. Declivity gradual, smooth, shiny, appearing bisulcate, occupying apical 2/5 of elytra; striae not impressed, striae 1 and 2 parallel, strial punctures much larger and shallower than those of disc; interstriae impunctate, interstriae 2 strongly sulcate, unarmed; interstriae 1 and 3 strongly costate with five and three granules respectively, each granule bearing a long, erect hair-like seta. Posterolateral margin with interstriae 3 and 9 joining, forming a granulate acute carina and continuing submarginally to apex. ***Legs***: protibiae obliquely triangular, broadest at apical 1/4; apical 1/2 of outer margin with six large, socketed denticles, their length longer than basal width. Meso- and metatibiae flattened; outer margin evenly rounded with seven large, socketed denticles.

##### Etymology.

Portrayed by Sigourney Weaver, Ellen Ripley is the heroine in the movies ‘Alien’ (1979), ‘Aliens’ (1986), ‘Alien 3’ (1992), and ‘Alien: Resurrection’ (1997). This species is glabrous and reminiscent of Ripley’s shaved head in ‘Alien 3’. Noun in apposition.

##### Distribution.

Ecuador (Orellana).

##### Biology.

The holotype was collected by canopy fogging.

#### Coptoborus sagitticauda sp. nov.

Taxon classificationAnimaliaColeopteraCurculionidae

B0E76B98-9800-5DBC-A25B-B77B3DECD8BA

http://zoobank.org/F8CABA24-9049-446C-B217-AE7914466D83

Figure 14G–I, O

##### Type material.

***Holotype*,** female, Guyana, Iwokrama Forest, 4°40.486'N, 58°41.028'W, 4–9.iii.2007, Cognato, Hulcr, Smith, Dole, McCall, GUY 17 (MSUC). ***Paratypes***, female, as holotype (MSUC, 3); as holotype except: GUY 15 (NHMUK, 2; NMNH, 3); as holotype except: GUY 19 (MSUC, 4; NHMUK, 1).

##### Diagnosis.

2.3 mm (mean = 2.3 mm; n = 5), 2.88 × as long as wide. This species is distinguished by the elytral apex strongly acuminate, declivital interstriae 2 granulate near apex, declivity with a costa extending from apex to interstriae 8 and pronotum 1.25 × as long as wide. It is most similar to *C. attenuatus* from which it can be distinguished by the larger size, 2.3 mm, vs. 2.0 mm and more elongate form, 2.88 × as long as wide vs. 2.5 × as long as wide).

##### Similar species.

*C. attenuatus*, *C. bellus*, *C. katniss*, *C. sarahconnor*, *C. sicula*, *C. yar*.

##### Description

**(female). *Holotype*** 2.3 mm, 2.88 × as long as wide. Body dark brown, antennae and legs lighter. ***Head***: epistoma smooth. Frons strongly shiny, finely punctate, setose; each puncture bearing a long, erect hair-like seta. Eyes broadly and moderately emarginate. Submentum large, triangular, deeply impressed. Antennal scape regularly thick, much shorter than club. Pedicel shorter than funicle. Club circular, flat, type 4; segment 1 corneous, narrow, acutely procurved on anterior face, occupying basal ~1/2; segment 2 narrow, procurved, corneous; segments 1 and 2 present on posterior face. ***Pronotum***: 1.3 × as long as wide. In dorsal view long and rounded frontally, type 7, sides parallel in basal 5/7, rounded anteriorly; anterior margin with two projecting serrations. In lateral view elongate, disc longer than anterior slope, type 7, summit prominent, on anterior 3/5. Anterior slope with densely spaced, broad fine asperities, becoming lower and more strongly transverse towards summit. Disc reticulate, dull, finely asperate, minutely punctate on basal quarter and lateral areas, punctures, some longer hair-like setae at margins. Lateral margins obliquely costate. ***Elytra***: 1.6 × as long as wide, 1.3 × as long as pronotum. Scutellum small. Elytra attenuate, parallel-sided in basal 62%, then acutely narrowed to acuminate apex. Disc smooth, shiny; strial punctures moderate, shallow, each bearing a recumbent hair-like seta the length of 2–3 punctures; interstriae flat, minutely, densely punctate, unarmed, glabrous. Declivity gradually rounded, occupying ~2/5 of elytra, smooth, shiny, declivital face weakly convex; striae not impressed, strial punctures larger, deeper than those of disc, each puncture bearing a semi-erect hair-like seta as long as three punctures; interstriae flat, uniformly denticulate, denticles distinct, small, interstriae 2–7 each with a row of erect setae as described for striae; interstriae 1 with two rows of setae. Posterolateral margin with a serrate costa from interstriae 8 to apex. ***Legs***: protibiae semi-circular with evenly rounded outer margin, broadest at apical 1/3; apical 1/2 of outer margin with six large, socketed denticles, their length longer than basal width. Meso- and metatibiae flattened; outer margin evenly rounded with seven large, socketed denticles.

##### Etymology.

L. *sagitta* = arrow, *cauda* = tail. Noun in apposition.

##### Distribution.

Guyana.

##### Biology.

Unknown.

#### Coptoborus sarahconnor sp. nov.

Taxon classificationAnimaliaColeopteraCurculionidae

511678B9-94E2-59B1-A11E-817E075962C6

http://zoobank.org/D2BB1B61-5415-489A-AF46-B7106C12A1BB

Figure 14J–L, P

##### Type material.

***Holotype*,** female, Brazil: [Pará], Santarém, Acc. No. 2966 (CNCI).

##### Diagnosis.

2.3 mm (n = 1), 2.88 × as long as wide. This species is distinguished by the elytral apex strongly acuminate, declivital interstriae unarmed along its entire length, antennal club with two sutures on posterior face, elytral discal interstriae punctate, and posterolateral margin of declivity with a very short carina extending from apex to interstriae 2.

##### Similar species.

*C. attenuatus*, *C. bellus*, *C. katniss*, *C. sagitticauda*, *C. sicula*, *C. yar*.

##### Description

**(female). *Holotype*** 2.3 mm, 2.88 × as long as wide. Body brown, antennae and legs lighter. ***Head***: epistoma tuberculate. Frons subshiny, finely punctate, setose; each puncture bearing a long, erect hair-like seta. Eyes broadly and moderately emarginate. Submentum large, triangular, deeply impressed. Antennal scape regularly thick, as long as club. Pedicel as long as funicle. Club circular, flat, type 3; segment 1 corneous, transverse on anterior face, occupying basal ~1/3; segment 2 narrow, transverse, corneous; segments 1 and 2 present on posterior face. ***Pronotum***: 1.3 × as long as wide. In dorsal view long and rounded frontally, type 7, sides parallel in basal 2/3, rounded anteriorly; anterior margin with two projecting serrations. In lateral view elongate, disc longer than anterior slope, type 7, summit prominent, on anterior 2/3. Anterior slope with densely spaced, broad fine asperities, becoming lower and more strongly transverse towards summit. Disc reticulate, dull with sparse, minute punctures, some longer hair-like setae at margins. Lateral margins carinate on basal third. ***Elytra***: 1.6 × as long as wide, 1.3 × as long as pronotum. Scutellum minute. Elytra attenuate, parallel-sided in basal 62%, then acutely narrowed to acuminate apex. Disc smooth, shiny; strial punctures large, shallow, each bearing a recumbent hair-like seta the length of three punctures; interstriae flat, minutely, densely punctate, unarmed, each puncture bearing a long semi-recumbent seta. Declivity gradually rounded, occupying ~2/5 of elytra, shagreened, dull, declivital face weakly convex; striae not impressed, strial punctures larger, deeper than those of disc, each puncture bearing a recumbent hair-like setae as long as two punctures; interstriae flat, nearly devoid of denticles except interstriae 3 coarsely serrate on acuminate projection, interstriae with a row of short erect hair-like setae. Posterolateral margin with a very short carina extending from apex to interstriae 2. ***Legs***: protibiae distinctly triangular, broadest at apical 1/5; apical 1/2 of outer margin with six large, socketed denticles, their length longer than basal width. Meso- and metatibiae flattened; outer margin evenly rounded with seven large, socketed denticles.

##### Etymology.

Portrayed by Linda Hamilton, Sarah Connor is a heroine in ‘The Terminator’ movie and television franchise (1984–2019). The vermiculate elytral declivity gives the species a rough appearance like the character it recognizes. Noun in apposition.

##### Distribution.

Brazil (Pará).

##### Biology.

Unknown.

#### Coptoborus schulzi

Taxon classificationAnimaliaColeopteraCurculionidae

Wood, 2007

6BA63975-CE4F-5A32-AB79-32B38D20D08D

Figure 15A–C, M

Coptoborus schulzi Wood, 2007: 394.

##### Type material.

***Holotype*** (NMNH), examined.

##### New records.

Ecuador: Napo Prov. [= Orellana Prov.], Res[erva]. Ethnica Waorani, 1 km S. Okone Gare Camp, Trans[ect]. Ent[omology]., 00°39'10"S, 076°26'W, 220 m, i.1996, T.L. Erwin et al. collectors, insecticidal fogging, terra firme forest, trans[ect] 7, sta[tion] 7, Erwin lot #1467 (NMNH, 1).

##### Diagnosis.

2.3 mm (n = 1), 2.56 × as long as wide. This species is distinguished by the elytral apex attenuate and weakly emarginate, declivital interstriae 2 convex, declivital interstriae 1–3 denticulate, posterolateral margin of declivity with interstriae 3 and 9 joining, forming a carina and continuing submarginally to apex, stout form, declivital interstriae 1 with a confused row of erect scale-like setae, and posterior ~40% of elytra acutely tapered to apex.

##### Similar species.

*C. barbicauda*, *C. bettysmithae*, *C. capillisoror*, *C. hansen*, *C. subtilis*, *C. trinity*, *C. uhura*.

##### Distribution.

Ecuador*(Orellana), Suriname.

##### Biology.

Unknown. A specimen was collected by canopy fogging.

**Figure 15.**
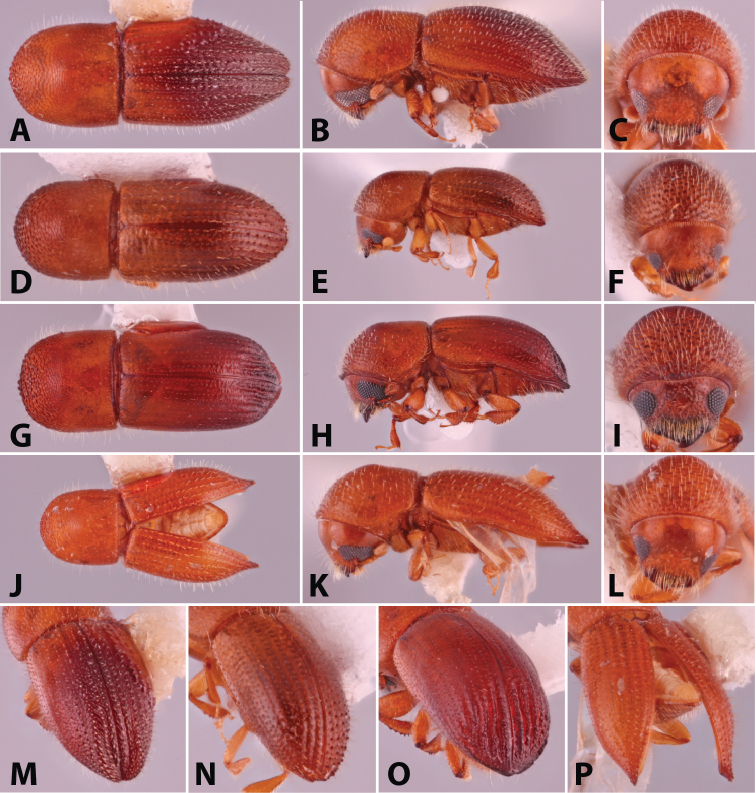
Dorsal, lateral, frontal and declivital view of *Coptoborus schulzi*, 2.3 mm (**A–C, M**), *C. scully* paratype, 1.7–2.0 mm (**D–F, N**), *C. semicostatus* 2.8–3.1 mm (**G–I, O**), *C. sicula* holotype, 2.1 mm (**J–L, P**). All photographs by SMS.

#### Coptoborus scully sp. nov.

Taxon classificationAnimaliaColeopteraCurculionidae

CA59D587-8662-5850-967D-4452AC863A9C

http://zoobank.org/F6BFCD3C-14AD-4B97-A385-DD33E624065F

Figure 15D–F, N

##### Type material.

***Holotype*,** female, Ecuador: Napo Prov. [= Orellana Prov.], Res[erva]. Ethnica Waorani, 1 km S. Okone Gare Camp, Trans[ect]. Ent[omology]., 00°39'10"S, 076°26'W, 220 m, January 2006, T.L. Erwin et al. collectors, insecticidal fogging, terra firme forest, trans[ect] 3, sta[tion] 1, Erwin lot #3120 (ICB). ***Paratypes***, female, as holotype except: trans[ect] 6, sta[tion] 1, Erwin lot #1221 (ICB, 1); as holotype except: July 1995, trans[ect] 4, sta[tion] 3, Erwin lot #1093 (NMNH, 1); as holotype except: January 1996, trans[ect] 6, sta[tion] 3, Erwin lot #1453 (ICB, 1); as holotype except October 1996, trans[ect] 6, sta[tion] 3, Erwin lot #1713 (ICB, 1); as holotype except: Tiputini Biodiversity Station, 00°37'55"S, 076°08'39"W, 220–250 m, February 1999, T.L. Erwin et al. collectors, insecticidal fogging, terra firme forest, trans[ect] 2, sta[tion] 6, Erwin lot #2015 (NMNH, 1); as holotype except: trans[ect] 5, sta[tion] 4, Erwin lot #2043 (MSUC, 1).

##### Diagnosis.

1.7–2.0 mm (mean = 1.84 mm; n = 5), 2.83–3.17 × as long as wide. declivital interstriae 2 denticulate along entire length, denticles as numerous and as large as those of interstriae 1, posterolateral margin of declivity costate, armed with three large denticles, and declivital slope steep.

##### Similar species.

*C. amplissimus*, *C. catulus*, *C. incomptus*, *C. newt*.

##### Description

**(female).** 1.7–2.0 mm (mean = 1.84 mm; n = 5), 2.83–3.17 × as long as wide (***holotype*** 2.0 mm, 3.08 × as long as wide). Body brown, antennae and legs lighter. ***Head***: epistoma tuberculate. Frons dull, finely punctate, setose; each puncture bearing a long, erect hair-like seta. Eyes narrowly and moderately emarginate. Submentum large, triangular, slightly impressed. Antennal scape short and thick, shorter than club. Pedicel shorter than funicle. Club circular, flat, type 3; segment 1 corneous, transverse on anterior face, occupying basal ~1/4; segment 2 narrow, transverse, corneous; segments 1 and 2 present on posterior face. ***Pronotum***: 1.2 × as long as wide. In dorsal view long and rounded frontally, type 7, sides parallel in basal 2/3, rounded anteriorly; anterior margin without serrations. In lateral view elongate, disc longer than anterior slope, type 7, summit prominent, on anterior 5/7. Anterior slope with densely spaced, broad fine asperities, becoming lower and more strongly transverse towards summit. Disc strongly shiny with sparse, minute punctures, some longer hair-like setae at margins. Lateral margins obliquely costate. ***Elytra***: 1.7–1.9 × as long as wide, 1.5 × as long as pronotum. Scutellum minute. Elytra attenuate, parallel-sided in basal 64–70%, then acutely rounded to apex, apex weakly emarginate. Disc smooth, shiny; striae minutely punctate, each puncture bearing a recumbent seta the length of a puncture; interstriae flat, sparsely, minutely punctate, unarmed, each puncture bearing a long, erect bristle-like seta. Declivity gradually rounded, occupying ~2/5 of elytra, smooth, shiny, declivital face convex; striae not impressed, strial punctures larger, deeper than those of disc, each puncture bearing a semi-recumbent seta as long as four punctures, striae 1 parallel to suture; interstriae flat, interstriae 1 and 3 each with seven and five respectively, uniformly spaced large denticles, interstriae 2 with seven denticles, denticles on interstriae 1–3 subequal, interstrial setae moderately dense erect bristle-like, interstriae 1 with an additional row of slightly shorter setae. Posterolateral margin of declivity costate, armed with three large serrations. ***Legs***: protibiae obliquely triangular, broadest at apical 1/3; apical 1/2 of outer margin with six large, socketed denticles, their length longer than basal width. Meso- and metatibiae flattened; outer margin evenly rounded with seven large, socketed denticles.

##### Etymology.

Portrayed by Gillian Anderson, Dana Scully is the heroine in the ‘X-Files’ television series (1993–2002, 2016) and movie (2008). We believe in the ‘Scully Effect’ (https://seejane.org/research-informs-empowers/the-scully-effect-i-want-to-believe-in-stem/) and hope future female scientists, real and fictional, continue to inspire children and young adults to pursue STEM careers. Noun in apposition.

##### Distribution.

Ecuador (Orellana).

##### Biology.

Specimens were collected by canopy fogging.

#### Coptoborus semicostatus

Taxon classificationAnimaliaColeopteraCurculionidae

(Schedl, 1948) comb. nov.

D138272A-56AC-55FD-B4B9-7C8E4218DFA9

Figure 15G–I, O

Xyleborus semicostatus Schedl, 1948: 268.
Dryocoetoides semicostatus (Schedl): Wood and Bright 1992: 658.

##### Type material.

***Holotype*** (NHMW), examined.

##### New records.

Bolivia: Santa Cruz Dist., Portrerillo del Guenda, Preserva Natural, 17°40'S, 63°27'W, 370 m, 12–13.x.2007, A.R. Cline & J.E. Wappes, ex BL/MV (CSCA, 2).

##### Diagnosis.

2.8–3.1 mm (mean = 3.0 mm; n = 3), 2.6–2.7 × as long as wide. This species is distinguished by the declivital interstriae raised into vermiculate ridges as high as 2 × strial width, declivity subshiny and smaller size, 3.1 mm.

##### Similar species.

*C. starbuck*, *Dryocoetoides* spp.

##### Distribution.

Bolivia* (Santa Cruz), Brazil (Mato Grosso do Sul).

##### Biology.

Unknown.

##### Remarks.

The species was undoubtedly placed in *Dryocoetoides* by Wood and Bright (1992) because of its costate interstriae, a usually diagnostic character for *Dryocoetoides* combined with an inflated and granulate posterior face of the protibia. However, the only previously known specimen, the holotype, is point mounted with an excessive amount of glue making it impossible to properly see the protibia. The protibiae are clearly visible on the Bolivian specimens and the posterior face is flat and unarmed. This species is therefore transferred to *Coptoborus*.

#### Coptoborus sicula sp. nov.

Taxon classificationAnimaliaColeopteraCurculionidae

B4016018-654F-5266-95E9-971B14C49141

http://zoobank.org/AB3EED1B-C3F1-4AF9-987D-22BF29E93479

Figure 15J–L, P

##### Type material.

***Holotype*,** female, Ecuador: Napo Prov. [= Orellana Prov.], Res[erva]. Ethnica Waorani, 1 km S. Okone Gare Camp, Trans[ect]. Ent[omology]., 00°39'10"S, 076°26'W, 220 m, July1996, T.L. Erwin et al. collectors, insecticidal fogging, terra firme forest, trans[ect] 1, sta[tion] 1, Erwin lot #1521 (ICB). ***Paratype***, female, as holotype except: Tiputini Biodiversity Station, 00°37'55"S, 076°08'39"W, 220–250 m, June 1998, T.L. Erwin et al. collectors, insecticidal fogging, terra firme forest, trans[ect] 6, sta[tion] 2, Erwin lot #1851 (NMNH, 1).

##### Diagnosis.

2.1 mm (n = 1), 3.0 × as long as wide. This species is distinguished by the elytral apex strongly acuminate, declivital interstriae 2 granulate near apex, declivity rounded, without a costa on posterolateral margin, and elytral discal interstriae 2 with one row of uniseriate punctures.

##### Similar species.

*C. attenuatus*, *C. bellus*, *C. katniss*, *C. sagitticauda*, *C. sarahconnor*, *C. yar*.

##### Description

**(female). *Holotype*** 2.1 mm, 3.0 × as long as wide. Body, antennae, and legs light brown. **Head**: epistoma smooth. Frons shiny, finely punctate, setose; each puncture bearing a long, erect hair-like seta. Eyes narrowly and moderately emarginate. Submentum narrow, triangular, deeply impressed. Antennal scape short and thick, as long as club. Pedicel shorter than funicle. Club circular, flat, type 3; segment 1 corneous, transverse on anterior face, occupying basal ~1/3; segment 2 narrow, transverse, corneous; segments 1 and 2 present on posterior face. **Pronotum**: 1.0 × as long as wide. In dorsal view basic and parallel-sided, type 2, sides parallel in basal 5/7, rounded anteriorly; anterior margin without serrations. In lateral view tall, type 2, disc flat, summit pronounced, at midpoint. Anterior slope with densely spaced, broad fine asperities, becoming lower and more strongly transverse towards summit. Disc strongly shiny with sparse, minute punctures, some longer hair-like setae at margins. Lateral margins obliquely costate. **Elytra**: 2.0 × as long as wide, 2.0 × as long as pronotum. Scutellum minute. Elytra attenuate, parallel-sided in basal 71%, then acutely narrowed to acuminate apex. Disc smooth, shiny; strial punctures large, shallow, glabrous; interstriae flat, minutely, densely punctate, unarmed, each puncture bearing a long semi-erect bristle-like seta. Declivity gradually rounded, occupying ~2/5 of elytra, smooth, shiny, declivital face strongly convex; striae not impressed, strial punctures larger, deeper than those of disc, glabrous; interstriae flat, uniformly granules, granules distinct, small, spaced by at least four diameters of a granule, interstriae with a row of moderately long bristle-like erect setae, as long as the width of interstriae 2.

Posterolateral margin rounded. **Legs**: protibiae obliquely triangular, broadest at apical 1/3; apical 1/2 of outer margin with seven large, socketed denticles, their length longer than basal width. Meso- and metatibiae flattened; outer margin evenly rounded with seven large, socketed denticles.

##### Etymology.

L. *sica* = dagger, -*ulus* = little. Noun in apposition.

##### Distribution.

Ecuador (Orellana).

##### Biology.

Specimens were collected by canopy fogging.

#### Coptoborus silviasilasi

Taxon classificationAnimaliaColeopteraCurculionidae

Atkinson, 2018

B963A777-E644-5F26-B37A-1DED593584C5

Figure 16A–C, L

Coptoborus silviasilasi Atkinson, 2018: 345.

##### Type material.

***Paratypes*** (MSUC, 2), examined.

##### New records.

None.

##### Diagnosis.

3.0 mm (mean = 3.0 mm; n = 2), 2.5 × as long as wide. This species is distinguished by the declivity broadly and deeply sulcate between interstriae 3, declivital interstriae 1 with a large digitate projection, its length ~2 × basal diameter, and a large digitate projection at the middle of the declivity on interstriae 3, and dark brown to black color.

##### Similar species.

None.

##### Distribution.

Mexico (Oaxaca).

##### Biology.

Atkinson (2018) reported collecting the species from an unidentified branch 2–5 cm in diameter in a coffee plantation.

**Figure 16.**
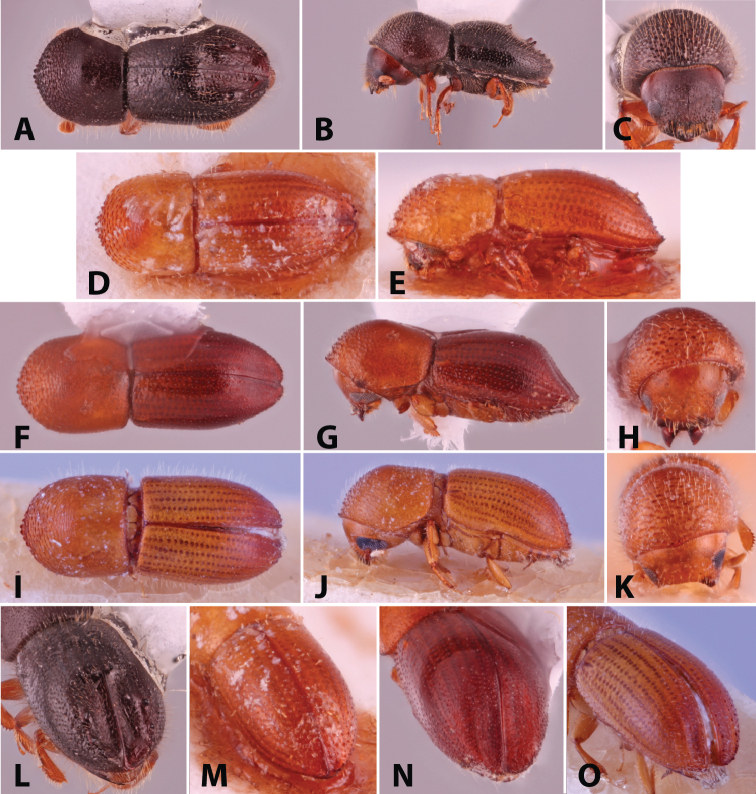
Dorsal, lateral, frontal and declivital view of *Coptoborus silviasilasi* paratype, 3.0 mm (**A–C, L**), *C. solitariformis* lectotype, 1.9 mm (**D, E, M**), *C. sororcula* holotype, 2.2 mm (**F–H, N**), *C. spicatus* paratype, 2.2 mm (**I–K, O**). All photographs by SMS.

#### Coptoborus solitariformis

Taxon classificationAnimaliaColeopteraCurculionidae

(Schedl, 1976)

1BC9692E-6174-5BE1-A1D6-5FBCD9E84404

Figure 16D, E, M

Xyleborus solitariformis Schedl, 1976: 77.
Dryocoetoides solitariformis (Schedl): Wood and Bright 1992: 658.
Coptoborus solitariformis (Schedl): Wood 2007: 396.

##### Type material.

***Lectotype*** (NHMW), examined.

##### New records.

None.

##### Diagnosis.

1.9 mm (n = 1), 2.38 × as long as wide. This species is distinguished by the elytral apex attenuate and weakly emarginate, declivital interstriae 2 convex, declivital interstriae 1–3 denticulate, denticles on interstriae 2 distinct, as large as those of interstriae 1 or 3, posterolateral margin of declivity with interstriae 3 and 9 joining, forming a carina and continuing submarginally to apex and declivity smooth and shiny.

##### Similar species.

*C. puertoricensis*.

##### Distribution.

Brazil (Mato Grosso).

##### Biology.

Unknown.

#### Coptoborus sororcula sp. nov.

Taxon classificationAnimaliaColeopteraCurculionidae

5297FAA9-912B-55F2-9536-4AA3BAB7D71D

http://zoobank.org/795152C7-C702-4EEB-A6F3-6258E2437270

Figure 16F–H, N

##### Type material.

***Holotype*,** female, Peru: Madre de Dios Dept., Los Amigos Biological Station, CM2, 12.4492°S, 70.2517°W, Smith, Hulcr, 17–18.v.2008, sample Peru 75, 3 cm diameter twig (MUSM).

##### Diagnosis.

2.2 mm (n = 1), 2.75 × as long as wide. This species is distinguished by the elytral apex attenuate and weakly emarginate, declivity feebly sulcate along interstriae 2, declivital interstriae 1–3 unarmed, declivity glabrous, declivital interstriae 3 feebly elevated, and declivity shagreened, dull.

##### Similar species.

*C. busoror*, *C. leeloo*, *C. nudulus*, *C. ochromactonus*, *C. pilisoror*, *C. ripley*, *C. spicatus*.

##### Description

**(female). *Holotype*** 2.2 mm, 2.75 × as long as wide. Body brown, elytra darker, antennae and legs lighter. ***Head***: epistoma smooth. Frons dull, finely punctate, setose; each puncture bearing a long, erect hair-like seta. Eyes narrowly and moderately emarginate. Antennal scape short and thick. ***Pronotum***: 1.1 × as long as wide. In dorsal view basic and parallel-sided, type 2, sides parallel in basal 5/7, rounded anteriorly; anterior margin with four serrations, median pair largest. In lateral view elongate, disc longer than anterior slope, type 7, summit prominent, on anterior 3/5. Anterior slope with densely spaced, broad coarse asperities, becoming lower and more strongly transverse towards summit. Disc reticulate, dull with sparse, minute punctures, some longer hair-like setae at margins. Lateral margins carinate on basal third. ***Elytra***: 1.6 × as long as wide, 1.6 × as long as pronotum. Scutellum minute. Elytra attenuate, parallel-sided in basal 62%, then acutely tapered to apex, apex weakly emarginate. Disc smooth, dull; strial punctures moderate, shallow, glabrous; interstriae flat, punctures dense and strongly confused, unarmed, glabrous. Declivity gradual, shagreened, dull, glabrous, appearing bisulcate, occupying apical 2/5 of elytra; striae not impressed, striae 1 and 2 parallel, strial punctures much larger and shallower than those of disc; interstriae impunctate, interstriae 2 feebly sulcate, unarmed; interstriae 1 and 3 feebly costate unarmed. Posterolateral margin with interstriae 3 and 9 joining, forming a serrate acute carina and continuing submarginally to apex. ***Legs***: Mesotibiae flattened; outer margin evenly rounded with six large, socketed denticles.

##### Etymology.

L. *soror* = sister, -*culus* = little. Noun in apposition.

##### Distribution.

Peru (Madre de Dios).

##### Biology.

The species was collected from a 3 cm diameter twig of an unidentified host.

#### Coptoborus spicatus

Taxon classificationAnimaliaColeopteraCurculionidae

Wood, 2007

D58F4F16-712F-57DD-AF74-4EA5DB4729F8

Figure 16I–K, O

Coptoborus spicatus Wood, 2007: 394.

##### Type material.

***Holotype*,*paratype*** (NMNH), examined.

##### New records.

None.

##### Diagnosis.

2.2 mm (n = 1), 2.44 × as long as wide. This species is distinguished by the elytral apex attenuate and weakly emarginate, declivity feebly sulcate along interstriae 2, declivital interstriae 2 unarmed, interstriae 1 and 3 armed by more six and four denticles, respectively, and declivity nearly glabrous.

##### Similar species.

*C. busoror*, *C. leeloo*, *C. nudulus*, *C. ochromactonus*, *C. pilisoror*, *C. ripley*, *C. sororcula*.

##### Distribution.

Suriname.

##### Biology.

Unknown.

#### Coptoborus starbuck sp. nov.

Taxon classificationAnimaliaColeopteraCurculionidae

57FA56DE-2766-5524-9B3D-FB68DE77DB47

http://zoobank.org/25229317-BC97-4CA7-9354-AC5EA1C063C0

Figure 17A–C, M

##### Type material.

***Holotype*,** female, Ecuador: Napo Prov. [= Orellana Prov.], Estación Cientifica Yasuní, 00°40'28"S, 76°38'50"W, 215 m, IX.5–10.1999, E.G. Riley, UV light, TAMU-ENTO X1305773 (TAMU). ***Paratype***, female, Ecuador: [Sucumbíos Prov.], Limoncocha, 0°23'S, 76°38'W, 300 m, 31.iii.1974, H.P. Stockwell (TAMU, 1).

##### Diagnosis.

3.3–3.6 mm (mean = 3.45 mm; n = 2), 2.54–2.57 × as long as wide. This species is distinguished by the declivital interstriae raised into vermiculate ridges as high as 4 × strial width, declivity shagreened and larger size, 3.6 mm.

##### Similar species.

*C. semicostatus*, *Dryocoetoides* spp.

##### Description

**(female).** 3.3–3.6 mm (mean = 3.45 mm; n = 2), 2.54–2.57 × as long as wide (***holotype*** 3.6 mm, 2.57 × as long as wide). Body, antennae and legs light brown, elytral declivity dark brown. ***Head***: epistoma tuberculate. Frons strongly shiny, finely punctate, setose; each puncture bearing a long, erect hair-like seta. Eyes broadly and moderately emarginate. Submentum large, triangular, deeply impressed. Antennal scape regularly thick, as long as club. Pedicel shorter than funicle. Club circular, flat, type 3; segment 1 corneous, subconvex on anterior face, occupying basal ~1/4; segment 2 broad, subconvex, corneous; segments 1 and 2 present on posterior face. ***Pronotum***: 1.0 × as long as wide. In dorsal view basic and parallel-sided, type 2, sides parallel in basal 2/3, rounded anteriorly; anterior margin with eight projecting serrations, median pair largest. In lateral view elongate, disc longer than anterior slope, type 7, summit prominent, on anterior 2/3. Anterior slope with densely spaced, broad coarse asperities, becoming lower and more strongly transverse towards summit. Disc reticulate, dull with sparse, minute punctures, some longer hair-like setae at margins. Lateral margins obliquely costate. ***Elytra***: 1.5 × as long as wide, 1.5 × as long as pronotum. Scutellum small. Elytra attenuate, parallel-sided in basal 80–81%, then acutely rounded to apex, apex entire. Disc shagreened, shiny; striae minutely punctate, glabrous; interstriae flat, minutely, sparsely punctate, unarmed, each puncture bearing a short recumbent hair-like seta. Declivity gradually rounded, occupying ~2/5 of elytra, shagreened, dull, declivital face convex; striae deeply impressed, strial punctures as large and deeper than those of disc, glabrous; interstriae raised into vermiculate ridges as high as 4 × strial width, each bearing short fine recumbent seta. Posterolateral margin apically produced, acutely carinate. ***Legs***: protibiae obliquely triangular, broadest at apical 1/4; apical 1/2 of outer margin with eight large, socketed denticles, their length longer than basal width. Meso- and metatibiae flattened; outer margin evenly rounded with ten and nine large, socketed denticles, respectively.

##### Etymology.

Portrayed by Katee Sackhoff, Kara ‘Starbuck’ Thrace is a heroine in the ‘Battlestar Galactica’ television series (2003–2009). The vermiculate elytral declivity gives the species a tough persona like the character it recognizes. Noun in apposition.

##### Distribution.

Ecuador (Orellana, Sucumbíos).

##### Biology.

Unknown.

**Figure 17.**
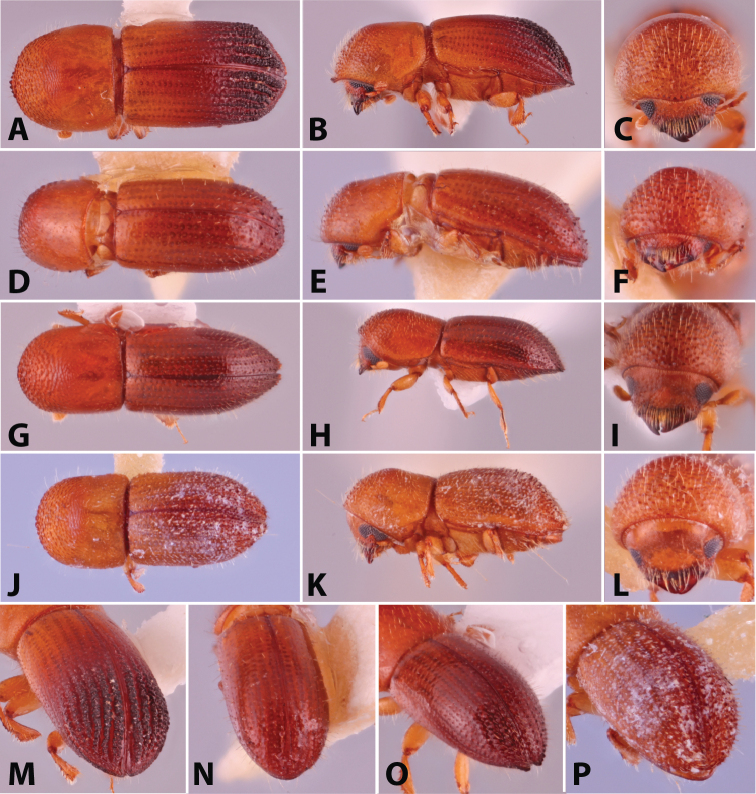
Dorsal, lateral, frontal and declivital view of *Coptoborus starbuck* holotype, 3.3–3.6 mm (**A–C, M**), *C. subtilis* holotype, 2.0 mm (**D–F, N**), *C. tolimanus*, 2.0–2.2 mm (**G–I, O**), *C. trinity* holotype, 2.0 mm (**J–L, P**). All photographs by SMS.

#### Coptoborus subtilis

Taxon classificationAnimaliaColeopteraCurculionidae

(Schedl, 1970)

64793E56-D13E-59E9-9F9D-B6AC74104542

Figure 17D–F, N

Xyleborus subtilis Schedl, 1970: 96.
Coptoborus subtilis (Schedl): Wood 2007: 395.

##### Type material.

***Holotype*** (NHMW), examined.

##### New records.

None.

##### Diagnosis.

2.0 mm (n = 1), 2.86 × as long as wide. This species is distinguished by the elytral apex attenuate and entire and not produced, declivity interstriae 2 feebly sulcate, declivital interstriae 1–3 denticulate, denticles on interstriae 1 and 3 very large and distinct, posterolateral margin of declivity with interstriae 3 and 9 joining, forming a carina and continuing submarginally to apex, stout form, and declivital interstriae setae sparse and bristle-like.

##### Similar species.

*C. barbicauda*, *C. bettysmithae*, *C. capillisoror*, *C. hansen*, *C. schulzi*, *C. trinity*, *C. uhura*.

##### Distribution.

Brazil (Santa Catarina).

##### Biology.

Unknown.

#### Coptoborus tolimanus

Taxon classificationAnimaliaColeopteraCurculionidae

(Eggers, 1928)

E0A7C25B-F151-55BA-A7C6-5F4BA4F63820

Figure 17G–I, O

Xyleborus tolimanus Eggers, 1928: 97
Coptoborus tolimanus (Eggers): Wood and Bright 1992: 664.

##### Type material.

***Lectotype*** (NMNH), examined.

##### New records.

Ecuador: Napo Prov. [= Orellana Prov.], Res[erva]. Ethnica Waorani, 1 km S. Okone Gare Camp, Trans[ect]. Ent[omology]., 00°39'10"S, 076°26'W, 220 m, July 1995, T.L. Erwin et al. collectors, insecticidal fogging, terra firme forest, trans[ect] 4, sta[tion] 8, Erwin lot #1098 (ICB, 1). French Guiana: Crique Alma Maripasoula, 2°14'2.47"N, 54°27'0.19"W, 12–20-VIII-2015, FIT with blue LED, E Poirier, P-H Dalens, F. Robin, Expedition “Our Planet Reviewed” Mitarka French Guiana 2015, MNHN/PNI & SEAG APA 973-1 (MSUC, 9); as previous except: pink LED (MSUC, 2). Panama: Panamá, Cd. Panamá, 13-IX-2007, I1WP (UTIC, 1); Canal Zone, Albrook Forest Site, Fort Clayton, May 16–17, 1968, Hutton (UAAM, 1).

##### Diagnosis.

2.0–2.2 mm (mean = 2.08 mm; n = 5), 2.86–3.38 × as long as wide. This species is distinguished by the elytral apex attenuate and strongly emarginate, declivity convex, declivital interstriae 2 denticulate, elytral apex with interstriae 3 and 9 joining, forming a crenulate carina that continues submarginally to apex, declivital interstriae 3 with fewer than ten denticles, elytral apices acute, declivital striae not impressed, elytral apex crenulations large and coarse, declivital slope steep, occupying 50% of elytra. It is most similar to *C. inornatus* but has more elongate elytra 1.7–2.0 × as long as wide vs. 1.6 × as long as wide, and larger size 2.0–2.2 mm vs. 1.8 mm.

##### Similar species.

*C. furiosa*, *C. inornatus*, *C. janeway*, *C. martinezae*, *C. vasquez*.

##### Distribution.

Brazil (Bahia, Espírito Santo, Mato Grosso, São Paulo), Colombia (Huila, Santander, Tolima, Valle de Cauca), Costa Rica (Cartago, Limón, Puntarenas), Ecuador* (Orellana), French Guiana*, Mexico (Hidalgo, Oaxaca, Tabasco, Veracruz), Panama (Coclé, Panamá*), Venezuela (Barinas).

##### Biology.

This species has been recorded from diverse hosts including: *Guatteria* (Annonaceae), *Protium* (Burseraceae), *Cordia* (Cordiaceae), *Inga* (Fabaceae), *Bombacopsis quinata*, *Heliocarpus appendiculatus*, *Theobroma cacao* (Malvaceae) (Wood and Bright 1992; Bright and Skidmore 1997; Wood 2007) and *Cecropia* (Urticaceae). Wood reported collecting specimens from limbs and branches 5–15 cm in diameter (Wood 1982, 2007). Specimens were also collected by canopy fogging.

##### Remarks.

Smith et al. (2017) reported this species from Peru (Madre de Dios). However this record represents a misidentification. The record is actually part of the type series of *Coptoborus janeway* from sample Peru 83b.

#### Coptoborus trinity sp. nov.

Taxon classificationAnimaliaColeopteraCurculionidae

05EAF2B0-4E45-5679-8EAC-AA2AF47760AF

http://zoobank.org/6104CE21-AF54-4E9E-A889-D26E1E07966A

Figure 17J–L, P

##### Type material.

***Holotype*,** female, Brazil: Mato Grosso, Sinop, x.1976, M. Alvarenga (CNCI).

##### Diagnosis.

2.0 mm (n = 1), 2.5 × as long as wide. This species is distinguished by the elytral apex attenuate and entire, declivital interstriae 2 convex, declivital interstriae 1–3 densely and coarsely denticulate, denticles large and very closely spaced, posterolateral margin of declivity with interstriae 3 and 9 joining, forming a carina and continuing submarginally to apex, convex declivity, and stout form.

##### Similar species.

*C. barbicauda*, *C. bettysmithae*, *C. capillisoror*, *C. hansen*, *C. schulzi*, *C. subtilis*, *C. uhura*.

##### Description

**(female). *Holotype*** 2.0 mm, 2.5 × as long as wide. Body, antennae and legs light brown. ***Head***: epistoma smooth. Frons shiny, finely punctate, setose; each puncture bearing a long, erect hair-like seta. Eyes broadly and moderately emarginate. Submentum narrow, triangular, deeply impressed. Antennal scape short and thick, much shorter than club. Pedicel shorter than funicle. Club circular, flat, type 4; segment 1 corneous, transverse on anterior face, occupying basal ~1/4; segment 2 narrow, corneous; segments 1 and 2 present on posterior face. ***Pronotum***: 1.0 × as long as wide. In dorsal view basic and parallel-sided, type 2, sides parallel in basal 2/3, rounded anteriorly; anterior margin with four subequal serrations. In lateral view tall, type 2, disc flat, summit pronounced, at midpoint. Anterior slope with densely spaced, broad fine asperities, becoming lower and more strongly transverse towards summit. Disc dull with sparse, minute punctures, some longer hair-like setae at margins. Lateral margins obliquely costate. ***Elytra***: 1.5 × as long as wide, 1.5 × as long as pronotum. Scutellum minute. Elytra attenuate, parallel-sided in basal 58%, then acutely rounded to apex, apex entire. Disc shagreened, subshiny; strial punctures moderate, shallow, glabrous; interstriae flat, densely, minutely punctate, unarmed, each puncture bearing a semi-erect spatulate seta. Declivity steeply rounded, occupying ~1/3 of elytra, shagreened, shiny, declivital face weakly convex; striae 1 and 2 distinctly impressed, strial punctures larger, deeper than those of disc, each puncture bearing a trifid recumbent seta as long as two punctures; interstriae flat, interstriae 2 and 3 densely uniseriate denticulate along their entire lengths, denticles spaced by one width of a denticle, interstriae 1 denticles confused, biseriate, setae erect, scale-like, as long as interstriae 1 width, becoming bristle-like and much longer on apical 1/4; interstriae 1 with an additional row of slightly shorter erect scale-like setae. Posterolateral margin with interstriae 3 and 9 joining, forming a granulate carina and continuing submarginally to apex. ***Legs***: protibiae obliquely triangular, broadest at apical 1/4; apical 1/2 of outer margin with six large, socketed denticles, their length longer than basal width. Meso- and metatibiae flattened; outer margin evenly rounded with seven large, socketed denticles.

##### Etymology.

Portrayed by Carrie-Anne Moss, Trinity is the heroine in the movies ‘The Matrix’ (1999), ‘The Matrix Reloaded’ (2003) and ‘The Matrix Revolutions’ (2003). Three types of setae (trifid, scale-like and bristle-like) help diagnose this species. Noun in apposition.

##### Distribution.

Brazil (Mato Grosso).

##### Biology.

Unknown.

#### Coptoborus tristiculus

Taxon classificationAnimaliaColeopteraCurculionidae

(Wood, 1975) comb. nov.

26E1DEF1-271A-5D06-AB73-7AE4A224AC5D

Figure 18A–C, M

Xyleborus tristiculus Wood, 1975b: 401.

##### Type material.

***Holotype*** (NHMUK), examined.

##### New records.

Ecuador: Napo Prov. [= Orellana Prov.], Res[erva]. Ethnica Waorani, 1 km S. Okone Gare Camp, Trans[ect]. Ent[omology]., 00°39'10"S, 076°26'W, 220 m, October 1996, T.L. Erwin et al. collectors, insecticidal fogging, terra firme forest, trans[ect] 7, sta[tion] 8, Erwin lot #1728 (ICB, 1); as previous except: October 1995, trans[ect] 2, sta[tion] 10, Erwin lot #1190 (ICB, 1); as previous except: January 1996, trans[ect] 7, sta[tion] 7, Erwin lot #1467 (NMNH, 1).

##### Diagnosis.

2.2–2.3 mm (mean = 2.23 mm; n = 2), 2.2–2.3 × as long as wide. This species is distinguished by the elytral apex broadly rounded and entire, posterolateral margin continuously and smoothly carinate to striae 6 and not extended posteriad, declivital interstrial setae fine, hair-like, longer than the width of interstriae 2 and abundantly covering declivity, declivital interstriae granulate, granules large and distinct, declivital striae 1 and 2 distinctly impressed.

##### Similar species.

*C. brigman*, *C. leia*, *Euwallacea perbrevis*.

##### Distribution.

Brazil (Mato Grosso), Ecuador* (Orellana).

##### Biology.

The type series were collected by Roger Beaver in Brazil. The type series have field notebook codes C47, D-35, Nos. 170, 172, No. E-18 on their locality labels (Wood 1975b). These specimens were collected from two small cut trees of *Protium* (Burseraceae) with stem diameters between 3.5–4.5 cm. Two gallery systems were investigated and branched rather irregularly in the transverse plane of the wood, penetrating ~2 cm deep, and were without enlargements or brood chambers (R.A. Beaver, pers. comm., 11 November 2020). Specimens were also collected by canopy fogging.

##### Remarks.

This species was omitted from Wood (2007). In his description Wood (1975b) allied this species to *Xyleborus molestulus* which he later placed in *Theoborus* (Wood and Bright 1992: 661) but which has recently been recognized as a synonym of *Euwallacea perbrevis* (Schedl, 1951) (Smith et al. 2020). This species shares the diagnostic characteristics of *Coptoborus* and is here transferred.

**Figure 18.**
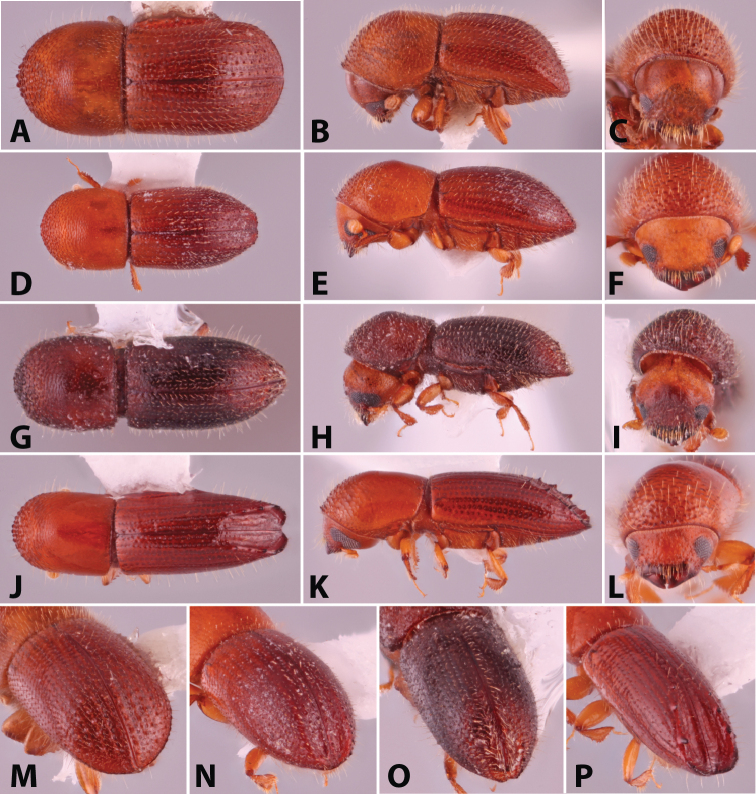
Dorsal, lateral, frontal and declivital view of *Coptoborus tristiculus* 2.2–2.3 mm (**A–C, M**), *C. uhura* holotype, 2.0 mm (**D–F, N**), *C. vasquez* holotype, 2.4 mm (**G–I, O**), *C. vespatorius*, 2.55–2.8 mm (**J–L, P**). All photographs by SMS.

#### Coptoborus uhura sp. nov.

Taxon classificationAnimaliaColeopteraCurculionidae

3580599C-A986-551F-AC88-69F3E67BC49A

http://zoobank.org/B1E841A8-C80A-46D0-BF7C-A80039ECB1CC

Figure 18D–F, N

##### Type material.

***Holotype*,** female, Peru: Madre de Dios Dept., Los Amigos Biological Station, 12°34.9S, 70°6.04W, Smith, Hulcr, 26.iv.–2.v.2008, sample Peru 6, branch (MUSM).

##### Diagnosis.

2.0 mm (n = 1), 2.55 × as long as wide. This species is distinguished by the elytral apex attenuate and entire and not produced, declivital interstriae 2 flattened, declivital interstriae 1–3 denticulate, posterolateral margin of declivity with interstriae 3 and 9 joining, forming a carina and continuing submarginally to apex, stout form, declivity weakly sulcate, denticles on interstriae 3 distinct, their height equal to interstriae width, and declivital interstriae setae uniseriate, sparse, bristle-like.

##### Similar species.

*C. barbicauda*, *C. bettysmithae*, *C. capillisoror*, *C. hansen*, *C. schulzi*, *C. subtilis*, *C. trinity*.

##### Description

**(female). *Holotype*** 2.0 mm (n = 1), 2.55 × as long as wide. Body, antennae and legs light brown, elytra ferruginous. ***Head***: epistoma smooth. Frons subshiny, finely punctate, setose; each puncture bearing a long, erect hair-like seta. Eyes broadly and moderately emarginate. Submentum large, triangular, deeply impressed. Antennal scape regularly thick, as long as club. Pedicel shorter than funicle. Club longer than wide, flat, type 3; segment 1 corneous, transverse on anterior face, occupying basal ~1/5; segment 2 broad, subconvex, corneous; segments 1 and 2 present on posterior face. ***Pronotum***: 1.0 × as long as wide. In dorsal view basic and parallel-sided, type 2, sides parallel in basal 5/7, rounded anteriorly; anterior margin without serrations. In lateral view tall, type 2, disc flat, summit pronounced, at midpoint. Anterior slope with densely spaced, narrow fine asperities, becoming lower and more strongly transverse towards summit. Disc strongly shiny with sparse, minute punctures, some longer hair-like setae at margins. Lateral margins obliquely costate. ***Elytra***: 1.6 × as long as wide, 1.5 × as long as pronotum. Scutellum minute. Elytra attenuate, parallel-sided in basal 65%, then acutely tapered to apex, apex entire. Disc smooth, subshiny; strial punctures moderate, shallow, glabrous; interstriae flat, sparsely, minutely punctate, unarmed, each puncture bearing a long, erect seta. Declivity gradually rounded, occupying ~1/2 of elytra, shagreened, dull, declivital face weakly sulcate; striae 1 deeply impressed, strial punctures larger, deeper than those of disc, each puncture bearing a semi-recumbent hair-like seta as long as a puncture; interstriae flat, sparsely and inconsistently denticulate, interstriae 1 and 3 denticles uniseriate, spaced by at least four widths of a denticle, interstriae 3 denticles distinct, their height equal to interstriae width, interstriae 2 with denticles only on basal third, setae sparse, uniseriate, short, erect, bristle-like, as long as interstriae 1 width. Posterolateral margin with interstriae 3 and 9 joining, forming a granulate carina and continuing submarginally to apex. ***Legs***: protibiae obliquely triangular, broadest at apical 1/4; apical 1/2 of outer margin with seven large, socketed denticles, their length longer than basal width. Meso- and metatibiae flattened; outer margin evenly rounded with seven large, socketed denticles.

##### Etymology.

Portrayed by Nichelle Nichols and Zoe Saldana, Uhura is a heroine in the ‘Star Trek’ television and movie franchise (1966–present). This species is reddish and reminiscent of the uniform Uhura wore on the original ‘Star Trek’ television show. Noun in apposition.

##### Distribution.

Peru (Madre de Dios).

##### Biology.

The species was collected from an unidentified branch.

#### Coptoborus vasquez sp. nov.

Taxon classificationAnimaliaColeopteraCurculionidae

84B588DE-410C-5CCE-BD88-6F37A97B76CB

http://zoobank.org/31AD601E-8D10-4A03-B2DB-DB2AD2E0A73F

Figure 18G–I, O

##### Type material.

***Holotype*,** female, Panama: Cd. [Ciudad] Panamá, 17-VIII-2002, E2PP (NMNH).

##### Diagnosis.

2.4 mm (n = 1), 3.0 × as long as wide. This species is distinguished by the elytral apex attenuate and weakly emarginate, declivity feebly sulcate along interstriae 2, declivital interstriae 2 unarmed, interstriae 1 and 3 armed by two large denticles, and declivity densely setose.

##### Similar species.

*C. furiosa*, *C. inornatus*, *C. janeway*, *C. martinezae*, *C. tolimanus*.

##### Description

**(female). *Holotype*** 2.4 mm, 3.0 × as long as wide. Body dark brown, antennae and legs lighter. ***Head***: epistoma tuberculate. Frons dull, finely punctate, setose; each puncture bearing a long, erect hair-like seta. Eyes emarginate. Submentum narrow, triangular, deeply impressed. Antennal scape regularly thick, as long as club. Pedicel shorter than funicle. Club circular, flat, type 4; segment 1 corneous, transverse on anterior face, occupying basal ~1/4; segment 2 narrow, corneous; segments 1 and 2 present on posterior face. ***Pronotum***: 1.0 × as long as wide. In dorsal view basic and parallel-sided, type 2, sides parallel in basal 2/3, rounded anteriorly, abundantly covered with long hair-like setae; anterior margin without serrations. In lateral view tall, type 2, disc flat, summit pronounced, at midpoint. Anterior slope with densely spaced, broad coarse asperities, becoming lower and more strongly transverse towards summit. Disc reticulate, dull with sparse, minute punctures. Lateral margins obliquely costate. ***Elytra***: 2.0 × as long as wide, 2.0 × as long as pronotum. Scutellum minute. Elytra attenuate, parallel-sided in basal 63%, then acutely tapered to apex, apex entire. Disc shagreened, dull; strial punctures small, deep, each bearing a recumbent seta the length of two punctures; interstriae flat, minutely, moderately punctate, unarmed, each puncture bearing a long semi-erect bristle-like seta. Declivity gradual, smooth, shiny, appearing bisulcate, occupying apical 2/5 of elytra; striae not impressed, striae 1 slightly laterally broadened from base to declivital midpoint and then narrowing towards apex, strial punctures larger and shallower than those of disc, each puncture bearing a semi-erect seta as long as four punctures; interstriae impunctate, interstriae 2 feebly sulcate, unarmed; interstriae 1 and 3 feebly each with two large denticles, interstrial setae dense, erect, thick, bristle-like, uniseriate, interstriae 1 with one additional row of shorter erect hair-like setae. Posterolateral margin with interstriae 3 and 9 joining, forming a granulate costa and continuing submarginally to apex. ***Legs***: protibiae obliquely triangular, broadest at apical 1/4; apical 1/2 of outer margin with six large, socketed denticles, their length longer than basal width. Meso- and metatibiae flattened; outer margin evenly rounded with eight large, socketed denticles.

##### Etymology.

Portrayed by Jenette Goldstein, Private Vasquez is a heroine in the movie ‘Aliens’ (1986). Noun in apposition.

##### Distribution.

Panama (Panamá).

##### Biology.

Unknown.

#### Coptoborus vespatorius

Taxon classificationAnimaliaColeopteraCurculionidae

(Schedl, 1931)

65F98E90-44C5-5006-8BB3-471635B6C54E

Figure 18J–L, P

Xyleborus emarginatus Hopkins, 1915: 53. Preoccupied by Eichhoff 1878.
Xyleborus vespatorius Schedl, 1931: 342 (new name for X. emarginatus Hopkins nec Eichhoff 1878).
Coptoborus vespatorius (Schedl): Wood and Bright 1992: 665.
Xyleborus corniculatus Schedl, 1948: 275. Synonymy: Wood 1972: 200.
Xyleborus corniculatulus Schedl, 1948: 275. Synonymy: Wood 1972: 200.

##### Type material.

***Holotype****Xyleborus emarginatus* (NMNH), examined. ***Holotype****Xyleborus corniculatus* (NHMW), examined. ***Holotype****Xyleborus corniculatulus* (NHMW), examined.

##### New records.

Ecuador: Los Ríos, Canton La Clementina, Samama Nature Reserve, 01°38.852'S, 79°19.867'W, 381–430 m, 13–15.V.2015, Cognato, Smith, Osborn, Martinez et al., EC13, ex 4 cm dia. hanging liana (MSUC, 7; PUCE, 3). Napo Prov. [= Orellana Prov.], Res[erva]. Ethnica Waorani, 1 km S. Okone Gare Camp, Trans[ect]. Ent[omology]., 00°39'10"S, 076°26'W, 220 m, January 1996, T.L. Erwin et al. collectors, insecticidal fogging, terra firme forest, trans[ect] 5, sta[tion] 4, Erwin lot #1444 (ICB, 1); as previous except: P.N. Yasuní, 00°40'32"S, 76°21'19W, 250 m, 19 Feb 2005, I. Rodríguez (PUCE, 1); as previous except: Tiputini Biodiversity Station, 00°37'55"S, 076°08'39"W, 220–250 m, June 1998, T.L. Erwin et al. collectors, insecticidal fogging, terra firme forest, trans[ect] 3, sta[tion] 9, Erwin lot #1828 (ICB, 1). Pichincha, Quito, Parque Metropolitano, 00°11'22"S, 78°29'38"W, 2810 m, 15 Apr 2006, A. Argot (PUCE, 1). French Guiana: Crique Alma Maripasoula, 2°14'2.47"N, 54°27'0.19W, 12–20-VIII-2015, FIT with pink LED, E Poirier, P-H Dalens, F. Robin, Expedition “Our Planet Reviewed” Mitarka French Guiana 2015, MNHN/PNI & SEAG APA 973-1 (MSUC, 1). Guyana: Iwokrama Forest, Turtle Mountain, 4°44.081'N, 58°42.830'W, 4–9.iii.2007, Cognato, Hulcr, Smith, Dole, McCall, GUY 39 (MSUC, 9).

##### Diagnosis.

2.55–2.8 mm (mean = 2.67 mm; n = 5), 3.19–3.50 × as long as wide. This species is distinguished by the elytra attenuate, apex emarginate, elytra deeply excavated between interstriae 3, excavated area unarmed and anterior margin of pronotum without serrations.

##### Similar species.

*C. obtusicornis*.

##### Distribution.

Argentina (Misiones), Brazil (Bahia, Espírito Santo, São Paulo), Colombia (Valle de Cauca), Costa Rica (Cartago, Limón), Ecuador* (Los Ríos, Orellana, Pichincha), French Guiana*, Grenada, Guyana*, Mexico (Oaxaca, Veracruz), Peru (Loreto, Madre de Dios), Saint Lucia, Venezuela (Aragua, Miranda).

##### Biology.

This species has been recorded from *Magifera indica* (Anacardiaceae), *Hevea brasiliensis* (Euphorbiaceae), *Inga* (Fabaceae), *Theobroma cacao* (Malvaceae), and *Cestrum* (Solanaceae) (Wood and Bright 1992; Bright and Skidmore 1997; Wood 2007). Wood (1982) reported collecting specimens from dying and recently cut limbs and boles 10–30 cm in diameter and that the gallery comprised a simple entrance tunnel that was expanded by the brood into a small tabular cavity that followed the grain of the wood. The species has also been recorded from a cut hanging liana 4 cm in diameter and by canopy fogging.

#### Coptoborus villosulus

Taxon classificationAnimaliaColeopteraCurculionidae

(Blandford, 1898) comb. nov.

A24EA293-03E5-503C-9AA6-EE013FD4BA68

Figure 19A–C, J

Xyleborus villosulus Blandford, 1898: 204.
Theoborus villosulus (Blandford): Wood and Bright 1992: 662.
Theoborus theobromae Hopkins, 1915: 57. syn. nov.
Xyleborus pseudococcotrypes Eggers, 1941: 105. Synonymy: Wood 1962: 79.
Xyleborus coccotrypoides Eggers, 1943: 388. Synonymy: Wood 1976: 349.
Xyleborus villosus Schedl, 1948: 270. Synonymy: Wood 1976: 34.
Xyleborus hirtellus Schedl, 1948: 271. Synonymy: Schedl 1952: 163.

##### Type material.

***Holotype****Xyleborus villosulus* (NHMUK), examined. ***Holotype****Theoborus theobromae* (NMNH), examiened. ***Lectotype****Xyleborus hirtellus* (NHMW).

***Holotype****Xyleborus pseudococcotrypes* (MNHN) not examined. ***Holotype****Xyleborus coccotrypoides* (MNHN), not examined. ***Syntypes****Xyleborus villosus* (NHMW), not examined. **New records.** Brazil: Bahia, Camacan, Serra Bonita Reserve, 15°23.429'S, 39°33.810'W, 700–100 m, 6–14.V.2013, AI Cognato, SM Smith, CAH Flechtmann (MSUC, 7). Ecuador: El Cotopaxi, La Mana, Yakusinchi Nature Reserve, 00°57.030'S, 79°08.717'W, 3–16.iii.2017, R Osborn, C Bateman, & M Martinez, ex galleries (MSUC, 1). Napo Prov. [= Orellana Prov.], Res[erva]. Ethnica Waorani, 1 km S. Okone Gare Camp, Trans[ect]. Ent[omology]., 00°39'10"S, 076°26'W, 220 m, October 1995, T.L. Erwin et al. collectors, insecticidal fogging, terra firme forest, trans[ect] 7, sta[tion] 1, Erwin lot #1581 (ICB, 1); as previous except: Tiputini Biodiversity Station, 00°37'55"S, 076°08'39"W, 220–250 m, February 1999, T.L. Erwin et al. collectors, insecticidal fogging, terra firme forest, trans[ect] 2, sta[tion] 1, Erwin lot #2010 (ICB, 1; NMNH, 1); as previous except: trans[ect] 5, sta[tion] 8, Erwin lot #2047 (ICB, 1); as previous except: June 1998, trans[ect] 7, sta[tion] 2, Erwin lot #1861 (NMNH, 1). Panama: Verguas Pr., 8 km W. Sante Fe, Cerro Tute, el 300 ft, 08°30'26"N, 81°6'49"W, 24-vii-1999, J.B. Woolley 99/053 (TAMU, 1).

##### Diagnosis.

1.7–2.2 mm (mean = 1.94 mm; n = 5), 2.43–2.57 × as long as wide. This species is distinguished by the elytral apex broadly rounded and entire, posterolateral margins of elytra rounded, declivity convex, interstriae never impressed, and discal interstrial punctures confused.

##### Similar species.

None.

##### Distribution.

Argentina (Tucumán), Bahamas, Barbados, Bolivia (Cochabamba), Brazil (Bahia*, Mato Grosso, Paraná, Santa Catarina, São Paulo), Colombia (Cundinamarca, Huila, Valle de Cauca), Costa Rica (Cartago, Limón, Puntarenas), Cuba, Dominica, Dominican Republic, Ecuador* (El Cotopaxi, Orellana), French Guiana (Cayenne), Grenada, Guadeloupe, Guatemala, Martinique, Mexico (Chiapas, Tabasco, Veracruz), Montserrat, Netherlands Antilles, Panama (Panamá, Veraguas*), Peru (Cusco, Junín, Madre de Dios), Saint Lucia, Saint Vincent and the Grenadines, Venezuela (Aragua, Barinas, Bolívar, Mérida).

##### Biology.

This species has been recorded from diverse hosts including: *Alexa imperatricis*, *Erythrina costaricensis*, *Inga* (Fabaceae), *Ochroma*, *Theobroma cacao* (Malvaceae), *Miconia* (Melastomataceae), *Guarea* (Meliaceae), *Ficus* (Moraceae), *Pinus elliotti* (Pinaceae), *Piper* sp., *Piper tucumanum* (Piperaceae), *Coffea* (Rubiaceae), *Cestrum* (Solanaceae), *Cecropia* (Urticaceae) (Wood and Bright 1992; Bright and Skidmore 1997; Wood 2007; Córdoba and Atkinson 2018). Wood (1982) reported collecting specimens from unthrifty, cut, or broken branches ~2–5 cm in diameter. Specimens were also collected by canopy fogging.

##### Remarks.

Wood (1982, 2007) considered *T. theobromae* and *T. villosulus* to be closely related and separated based only on minute differences in pronotal puncture size, granule density and body size, 1.8 vs. 2.3 mm, respectively. Wood also considered these species to have overlapping ranges with *T. theobromae* in the West Indies and Costa Rica to Colombia and Venezuela and *T. villosulus* occurring from Guatemala to Bolivia and Brazil. Specimens used as part of this study were found to be continuous in size from 1.7–2.2 mm and the punctures and granules to form a continuous spectrum of variation as well. Extensive COI and CAD sampling from many of the populations given above had differences of < 10% and < 2%, respectively (Cognato et al. 2020b), supporting the recognition of a single species, *T. villosulus* (Cognato, unpublished).

**Figure 19.**
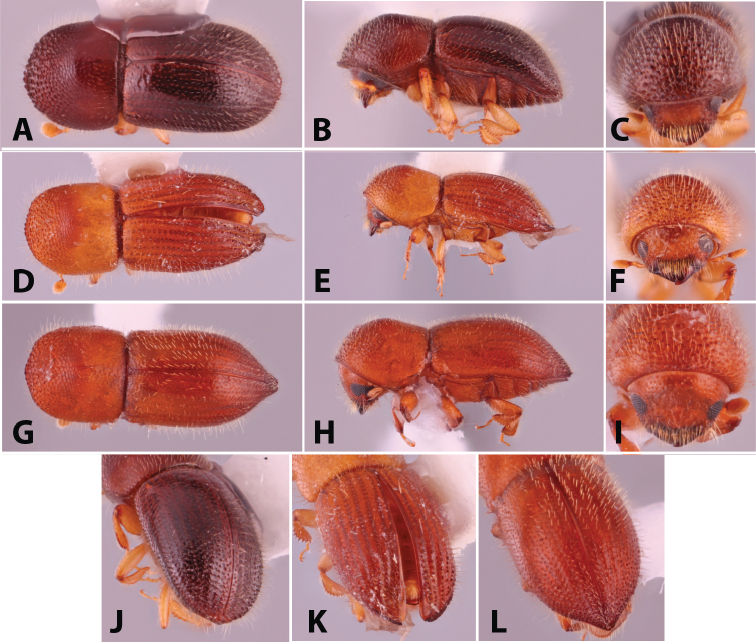
Dorsal, lateral, frontal and declivital view of *Coptoborus villosulus*, 1.7–2.2 mm (**A–C, J**), *C. vrataski* holotype, 3.2 mm (**D–F, K**), *C. yar* holotype, 2.8–2.9 mm (**G–I, L**). All photographs by SMS.

#### Coptoborus vrataski sp. nov.

Taxon classificationAnimaliaColeopteraCurculionidae

4D717AAB-2380-5376-A387-20D0B6D5BDB8

http://zoobank.org/7B45F884-B3BA-47D0-B6BE-DB3D66ABA3BC

Figure 19D–F, K

##### Type material.

***Holotype*,** female, Brazil: Rondônia, 62 km SW Ariquemes, nr Fzda. Rancho Grande, 3–15-XII-1996, JE Eger, black light trap (FSCA).

##### Diagnosis.

3.2 mm (n = 1), 2.67 × as long as wide. This species is distinguished by the elytra attenuate, elytra deeply sulcate along interstriae 2, interstriae 2 densely granulate, posterolateral margin of elytra carinate from apex to interstriae 7, and declivital interstriae densely covered with long thick erect scale-like setae.

##### Similar species.

*C. panosus*.

##### Description

**(female). *Holotype*** 3.2 mm, 2.67 × as long as wide. Body dark brown, antennae and legs lighter. ***Head***: epistoma tuberculate. Frons dull, finely punctate, setose; each puncture bearing a long, erect hair-like seta. Eyes broadly and moderately emarginate. Submentum narrow, triangular, deeply impressed. Antennal scape regularly thick, as long as club. Pedicel shorter than funicle. Club longer than wide, flat, type 4; segment 1 corneous, convex on anterior face, occupying basal ~1/4; segment 2 narrow, subconvex, corneous segments 1 and 2 present on posterior face. ***Pronotum***: 1.0 × as long as wide. In dorsal view basic and parallel-sided, type 2, sides parallel in basal 2/3, rounded anteriorly, abundantly covered with long hair-like setae; anterior margin with four serrations, median pair larger. In lateral view tall, type 2, disc flat, summit pronounced, at midpoint. Anterior slope with densely spaced, narrow coarse asperities, becoming lower and more strongly transverse towards summit. Disc strongly shiny with sparse, minute punctures, some longer hair-like setae at margins. Lateral margins obliquely costate. ***Elytra***: 1.7 × as long as wide, 1.7 × as long as pronotum. Scutellum small. Elytra attenuate, parallel-sided in basal 70%, then acutely rounded to apex, apex entire. Disc shagreened, dull; striae minutely punctate, each puncture bearing a recumbent hair-like seta the length of three punctures; interstriae flat, minutely, densely punctate, unarmed, each puncture bearing a long, erect hair-like seta. Declivity steep, occupying ~1/3 of elytra, shagreened, shiny, declivital face weakly convex; striae 1 and 2 deeply impressed, striae 3 weakly impressed, strial punctures larger, deeper than those of disc, each puncture bearing a semi-erect seta as long as three punctures; interstriae 2 deeply sulcate; interstriae densely granulate, granules large, separated by the distance of one granule, interstriae densely setose, setae long thick erect scale-like, twice as long as interstriae 1 width. Posterolateral margin apically produced, sharply carinate. ***Legs***: protibiae obliquely triangular, broadest at apical 1/4; apical 1/2 of outer margin with six large, socketed denticles, their length longer than basal width. Meso- and metatibiae flattened; outer margin evenly rounded with eight large, socketed denticles.

##### Etymology.

Portrayed by Emily Blunt, Sergeant Rita Vrataski, the ‘Angel of Verdun’ is the heroine in the movie ‘Edge of Tomorrow’ (2014). The granulate elytral gives the species an armored appearance reminiscent of the character’s combat jacket. Noun in apposition.

##### Distribution.

Brazil (Rondônia).

##### Biology.

Unknown.

#### Coptoborus yar sp. nov.

Taxon classificationAnimaliaColeopteraCurculionidae

8B4E565D-41E3-576C-A481-41B23574D452

http://zoobank.org/744A214D-D468-42CA-B8AF-80DEBA8D6F44

Figure 19G–I, L

##### Type material.

***Holotype*,** female, Ecuador: Fco. Orellana, P.N. Yasuní, 00°40'32"S, 76°21'19"W, 250 m, 19.ii.2005, I. Rodríguez (PUCE). ***Paratype***, female, as holotype except: Tiputini Biodiversity Station, 00°38.189'S, 76°08.965'W, 223 m, 3–9.VI.2011, S.M. Smith (MSUC, 1).

##### Diagnosis.

2.8–2.9 mm (mean = 2.85 mm; n = 2), 2.8–2.9 × as long as wide. This species is distinguished by the elytral apex strongly acuminate, declivital interstriae 2 granulate near apex, declivity rounded, with a very short carina on posterolateral margin extending from apex to interstriae 2, and elytral discal interstriae 2 with two rows of confused punctures.

##### Similar species.

*C. attenuatus*, *C. bellus*, *C. katniss*, *C. sagitticauda*, *C. sarahconnor*, *C. sicula*.

##### Description

**(female).** 2.8–2.9 mm (mean = 2.85 mm; n = 2), 2.8–2.9 × as long as wide (***holotype*** 2.8 mm, 2.8 × as long as wide). Body, antennae and legs light brown. **Head**: epistoma tuberculate. Frons dull, finely punctate, setose; each puncture bearing a long, erect hair-like seta. Eyes narrowly and moderately emarginate. Submentum large, triangular, deeply impressed. Antennal scape regularly thick, shorter than club. Pedicel shorter than funicle. Club longer than wide, flat, type 4; segment 1 corneous, transverse on anterior face, occupying basal ~1/4; segment 2 narrow, subconvex, corneous; segments 1 and 2 present on posterior face. **Pronotum**: 1.0–1.2 × as long as wide. In dorsal view long and rounded frontally, type 7, sides parallel in basal 3/4, rounded anteriorly, abundantly covered with long hair-like setae; anterior margin with/out serrations. In lateral view elongate, disc longer than anterior slope, type 7, summit prominent, on anterior 3/5. Anterior slope with densely spaced, broad fine asperities, becoming lower and more strongly transverse towards summit. Disc strongly shiny with sparse, minute punctures, some longer hair-like setae at margins. Lateral margins obliquely costate. **Elytra**: 1.8 × as long as wide, 1.8 × as long as pronotum. Scutellum minute. Elytra attenuate, parallel-sided in basal 2/3, then acutely narrowed to acuminate apex. Disc smooth, shiny; strial punctures large, shallow, glabrous; interstriae flat, minutely, densely punctate, unarmed, interstriae 2 with two rows of confused punctures, each puncture bearing a long semi-recumbent seta. Declivity gradually rounded, occupying ~2/5 of elytra, smooth, shiny, declivital face strongly convex; striae not impressed, strial punctures larger, deeper than those of disc, each puncture bearing a semi-recumbent seta as long as two punctures; interstriae flat, minutely granulate, granules becoming denser towards apex, interstriae 1–7 each with a row of moderately long thick erect setae, as long as the width of interstriae 1; interstriae 1 with an additional row of slightly shorter erect hair-like setae. Posterolateral margin with a very short carina extending from apex to interstriae 2. **Legs**: protibiae obliquely triangular, broadest at apical 1/4; apical 1/2 of outer margin with six large, socketed denticles, their length longer than basal width. Meso- and metatibiae flattened; outer margin evenly rounded with nine and ten large, socketed denticles, respectively.

##### Etymology.

Portrayed by Denise Crosby, Tasha Yar is a heroine in the first season of ‘Star Trek: The Next Generation’ (1987). Noun in apposition.

##### Distribution.

Ecuador (Orellana).

##### Biology.

Unknown.

## Discussion

This review of *Coptoborus* is the first to synonymize *Theoborus* based on morphological similarities of generic diagnostic characters and on a molecular phylogeny in which the species of the genera were not reciprocally monophyletic (Cognato et al. 2011; Cognato, unpublished). The genus now contains 77 species and we described 52% of the fauna (40 spp.). This suggests that the genus is very diverse and many undescribed species await discovery. For comparison, Smith et al. (2020) and Cognato et al. (2020a) only found 66 new species among 315 species in 34 genera in Southeast Asia. Most of the *Coptoborus* diversity has been found in Ecuador – 45% compared to Brazil (31%), Peru (29%), Panama (16%), Costa Rica (16%), Venezuela (10%), and Colombia (8%). Potentially, these differences may represent true variation in the diversity of these species and not a reflection of differences in collecting effort. Targeted scolytine collecting by Stephen Wood from fallen trees and branches for over several weeks occurred in Costa Rica, Venezuela, and Colombia but yielded only a small proportion of *Coptoborus* spp. (Wood 1982, 2007; Bright 2010). However, 32% of *Coptoborus* species (60% undescribed) were fogged from the Ecuadorian canopy. Thus differences in diversity among countries may reflect differences in collecting methods (canopy fogging vs. excising specimens from wood). Other scolytine genera fogged from the Ecuadorian canopy are similarly diverse, for example, 84% of *Scolytodes* Ferrari, 1867 (Hexacolini) and 23% of *Camptocerus* Dejean, 1821 (Scolytini) were undescribed (Jordal and Smith 2020; Smith and Cognato 2010).

Most *Coptoborus* (40 of 77) species were originally described from single specimens, 52%. With the exception of a few common species such as, *C. pseudotenuis*, *C. ricini*, *C. villosulus*, and *C. vespatorius*, specimens of most species are infrequently collected and 29 of 77 (37%) species are still only known from their holotypes. We do not doubt the validity of species we described based on singletons. The gaps of morphological differences between singletons and similar species were consistent with differences observed for species described from a series of specimens. These morphological differences, often minute, associate with large genetic differences that exceed the threshold for species recognition (Cognato et al. 2020b; Cognato unpublished).

The generic limits of South American xyleborines are clearly in need of further review. Most genera have received only cursory review, without the aid of a phylogeny, since their description (e.g., Wood 1986, 2007). The original descriptions and subsequent reviews do not consider the range of morphological variation among species for each genus. For example, previous taxonomists have relied on the diagnostic characters of original descriptions of *Coptoborus* and *Theoborus* to maintain their distinction. As detailed in the introduction, this was primarily based on body shape (Wood 2007; Bright 2019). Our review of all species in both genera demonstrated that these characters were not consistently associated with each other or monophyletic groups. Thus, we synonymized *Theoborus* with *Coptoborus* and described additional diagnostic characters. In another example, we discovered three characters that were inconsistent with the generic concept of *Sampsonius* and suggested a greater affinity of *S. obtusicornis* with *Coptoborus*. Based on our survey of Neotropical xyleborine specimens for this study, more species wait for correct generic placement or description as new genera. For example, several recent descriptions of new genera have removed some species from *Xyleborus* (Smith 2017; Atkinson et al. 2018) however *Xyleborus* remains a polyphyletic group. Total revision of Neotropical xyleborines will be best realized in the context of a molecular phylogeny and review of type specimens.

## Supplementary Material

XML Treatment for Coptoborus

XML Treatment for Coptoborus amazonicus

XML Treatment for Coptoborus amplissimus

XML Treatment for Coptoborus artetenuis

XML Treatment for Coptoborus asperatus

XML Treatment for Coptoborus atlanticus

XML Treatment for Coptoborus attenuatus

XML Treatment for Coptoborus barbicauda

XML Treatment for Coptoborus bellus

XML Treatment for Coptoborus bettysmithae

XML Treatment for Coptoborus brevicauda

XML Treatment for Coptoborus brigman

XML Treatment for Coptoborus busoror

XML Treatment for Coptoborus capillisoror

XML Treatment for Coptoborus carumbensis

XML Treatment for Coptoborus catulus

XML Treatment for Coptoborus chica

XML Treatment for Coptoborus coartatus

XML Treatment for Coptoborus cracens

XML Treatment for Coptoborus crassisororcula

XML Treatment for Coptoborus crinitulus

XML Treatment for Coptoborus cuneatus

XML Treatment for Coptoborus doliolum

XML Treatment for Coptoborus erwini

XML Treatment for Coptoborus exilis

XML Treatment for Coptoborus exutus

XML Treatment for Coptoborus furiosa

XML Treatment for Coptoborus galacatosae

XML Treatment for Coptoborus gentilis

XML Treatment for Coptoborus gracilens

XML Treatment for Coptoborus hansen

XML Treatment for Coptoborus incomptus

XML Treatment for Coptoborus incultus

XML Treatment for Coptoborus inornatus

XML Treatment for Coptoborus janeway

XML Treatment for Coptoborus katniss

XML Treatment for Coptoborus leeloo

XML Treatment for Coptoborus leia

XML Treatment for Coptoborus leporinus

XML Treatment for Coptoborus magnus

XML Treatment for Coptoborus martinezae

XML Treatment for Coptoborus micarius

XML Treatment for Coptoborus murinus

XML Treatment for Coptoborus newt

XML Treatment for Coptoborus nudulus

XML Treatment for Coptoborus obtusicornis

XML Treatment for Coptoborus ochromactonus

XML Treatment for Coptoborus osbornae

XML Treatment for Coptoborus panosus

XML Treatment for Coptoborus papillicauda

XML Treatment for Coptoborus paurus

XML Treatment for Coptoborus pilisoror

XML Treatment for Coptoborus pristis

XML Treatment for Coptoborus pseudotenuis

XML Treatment for Coptoborus puertoricensis

XML Treatment for Coptoborus ricini

XML Treatment for Coptoborus ripley

XML Treatment for Coptoborus sagitticauda

XML Treatment for Coptoborus sarahconnor

XML Treatment for Coptoborus schulzi

XML Treatment for Coptoborus scully

XML Treatment for Coptoborus semicostatus

XML Treatment for Coptoborus sicula

XML Treatment for Coptoborus silviasilasi

XML Treatment for Coptoborus solitariformis

XML Treatment for Coptoborus sororcula

XML Treatment for Coptoborus spicatus

XML Treatment for Coptoborus starbuck

XML Treatment for Coptoborus subtilis

XML Treatment for Coptoborus tolimanus

XML Treatment for Coptoborus trinity

XML Treatment for Coptoborus tristiculus

XML Treatment for Coptoborus uhura

XML Treatment for Coptoborus vasquez

XML Treatment for Coptoborus vespatorius

XML Treatment for Coptoborus villosulus

XML Treatment for Coptoborus vrataski

XML Treatment for Coptoborus yar
